# Synthesis and solvodynamic diameter measurements of closely related mannodendrimers for the study of multivalent carbohydrate–protein interactions

**DOI:** 10.3762/bjoc.10.157

**Published:** 2014-07-04

**Authors:** Yoann M Chabre, Alex Papadopoulos, Alexandre A Arnold, René Roy

**Affiliations:** 1 Pharmaqam, Department of Chemistry, Université du Québec à Montréal, P. O. Box 8888, Succ. Centre-ville, Montréal, Québec, Canada H3C 3P8

**Keywords:** carbohydrates, click chemistry, dendrimers, glycodendrimers, lectins, multivalent glycosystems

## Abstract

This paper describes the synthesis of three closely related families of mannopyranoside-containing dendrimers for the purpose of studying subtle structural parameters involved in the measurements of multivalent carbohydrate–protein binding interactions. Toward this goal, two trimers **5** and **9**, three 9-mers **12**, **17**, **21**, and one 27-mer **23**, varying by the number of atoms separating the anomeric and the core carbons, were synthesized using azide–alkyne cycloaddition (CuAAc). Compound **23** was prepared by an efficient convergent strategy. The sugar precursors consisted of either a 2-azidoethyl (**3**) or a prop-2-ynyl α-D-mannopyranoside (**7**) derivative. The solvodynamic diameters of 9-mer **12**, **17**, and **21** were determined by pulsed-field-gradient-stimulated echo (PFG-STE) NMR experiments and were found to be 3.0, 2.5, and 3.4 nm, respectively.

## Introduction

Multivalent carbohydrate–protein interactions are at the forefront of a wide range of biological events which have triggered a plethora of versatile synthetic methods for the design of potent inhibitors and glycomimetics [1–4]. Among the diverse strategies leading to efficient ligands, glycopolymers [1,5–7], glycodendrimers [7–14], and sugar rods [15–16] have retained most attention. An additional approach that has gained keen interest resides in the modifications of both the aglycon [17–19] and substituent residues [20–22] of the targeted sugar moieties through extensive studies of quantitative structure–activity relationships (QSARs). In most of the studies related to aglycon modifications, it was concluded that aromatic glycosides possessed improved binding properties due to the ubiquitous presence of aromatic amino acids in the cognate binding sites [23–25]. This is also supported by the recent findings that the sugar backbones themselves also possess a hydrophobic side that orients the sugars in appropriate aromatic amino acid rich pockets [26–28].

Unfortunately, due to the inherent complexity of studying multivalent binding interactions, researchers have used experimental conditions that often biased the intrinsic phenomena under investigations [29]. For instance, when evaluating thermodynamic parameters by isothermal calorimetry (ITC), scientists used either truncated versions of for instance, tetrameric lectins such as ConA, or diluted conditions to avoid precipitation of the complexes [30–31]. Alternatively, the application of surface plasmon resonance (SPR) also creates artificial situations not sufficiently related to the natural cellular events, thus requiring complex mathematical algorithms [32]. Most solid-phase immunoassays (ELLA, ELISA) also fall under the same criticism by providing unusually high (or too close) sugar densities. Also important and in spite of the two decades of glycodendrimer chemistry [7], there is still no general rule to allow predicting which structural parameters would be optimal for the binding interactions.

In order to gain more insight into this direction, we designed herein three families of closely related mannopyranoside clusters (glycodendrimers) aimed at evaluating their relative binding abilities against the hometetrameric leguminous lectin ConA from *Canavalia ensiformis* by inhibition of haemagglutination and by turbidimetry. The latter would allow us to measure relative kinetic factors involved in the cross-linking lattice formation using soluble partners.

## Results and Discussion

**Scheme 1:**
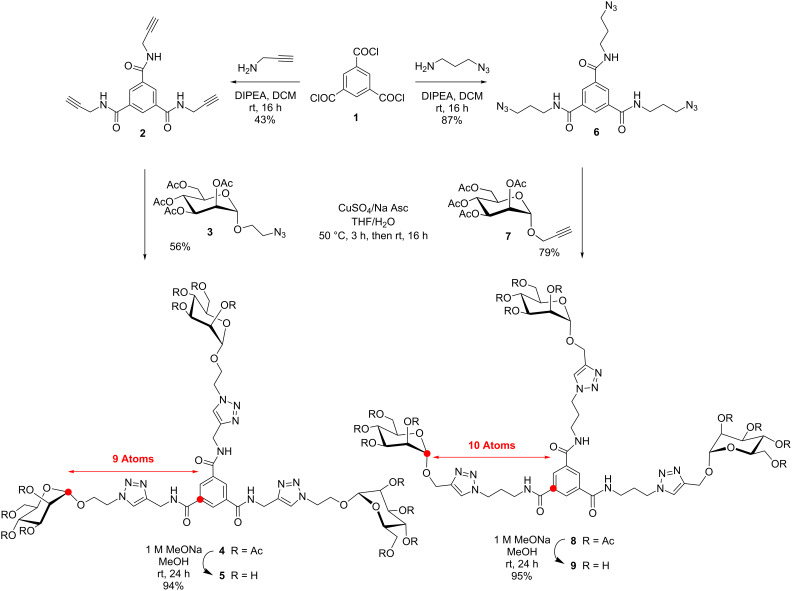
Synthesis of mannosylated trimers **5** and **9** using trimesic acid core transformed into propargylated (**2**) and azidopropylated (**6**) scaffolds and then coupled by “click chemistry” with either 2-azidoethyl (**3**) or propargyl (**7**) mannopyranosides.

**Scheme 2:**
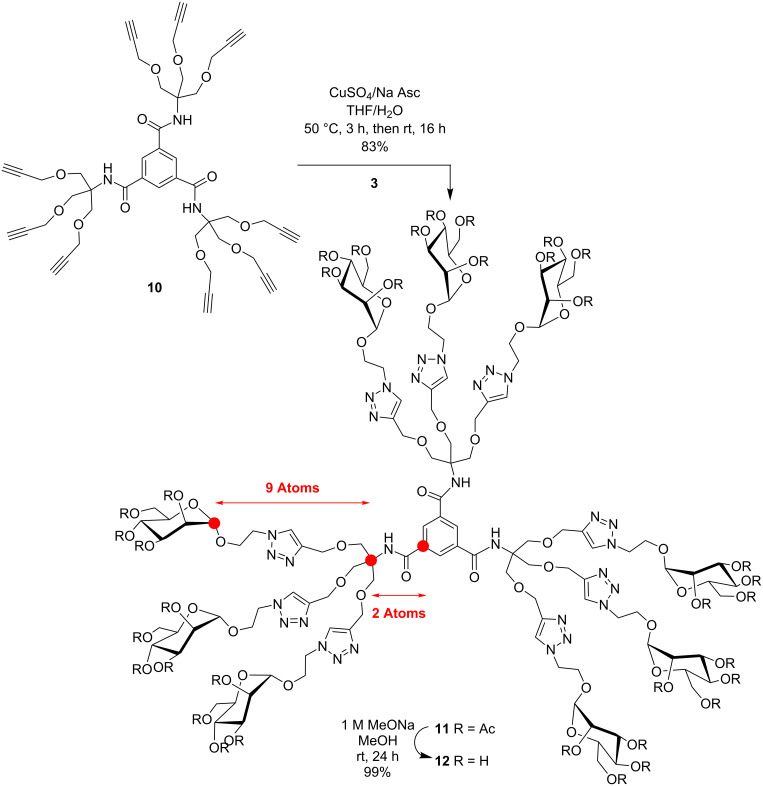
Divergent CuAAc “click reaction” between propargylated core **10** and azide **3** to afford 9-mer **12**.

**Scheme 3:**
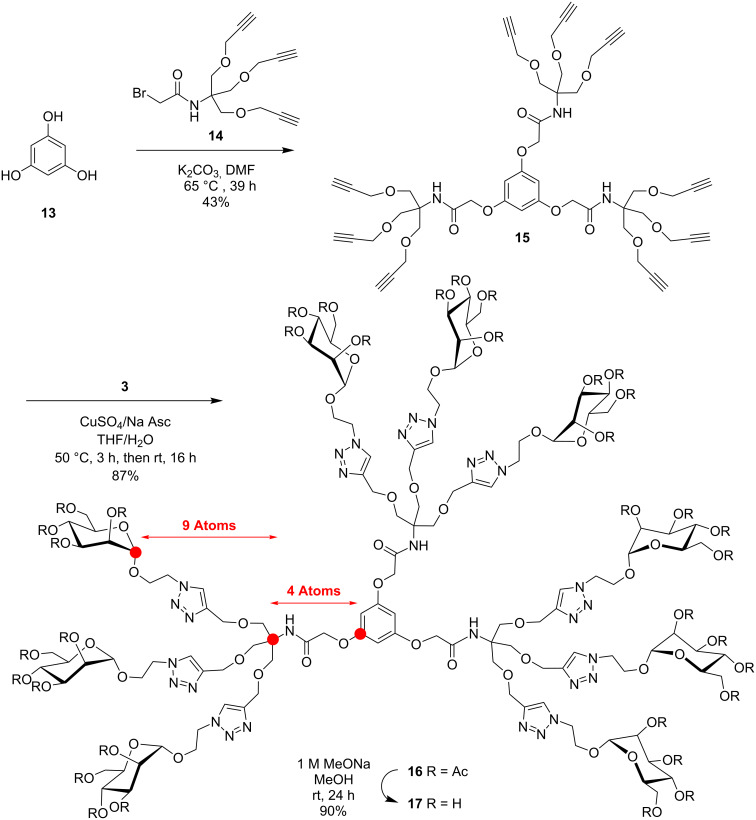
Divergent CuAAc synthesis of “extended” 9-mer **17** using phloroglucinol (**13**) as core, bromoacylated TRIS as linker and mannopyranosylazide **3**.

**Scheme 4:**
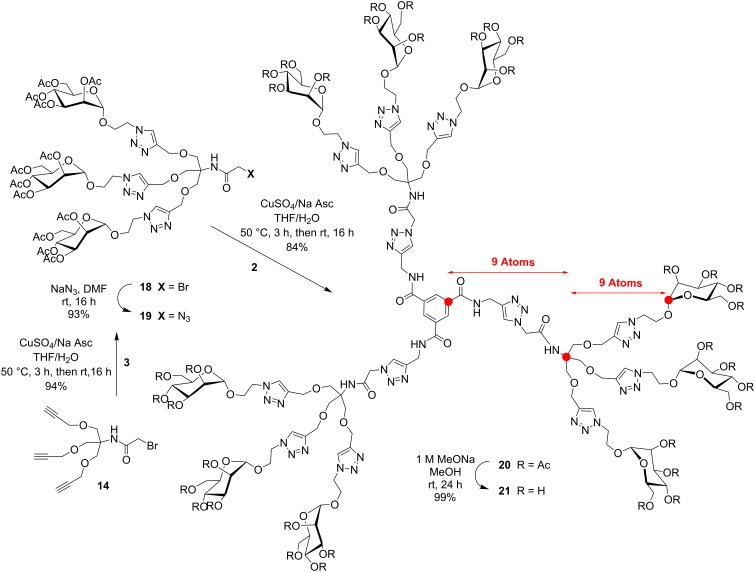
Convergent synthesis of further “extended” 9-mer **21** using mannosylated bromoacyl dendron **18** transformed into azide **19** followed by CuAAc coupling to tripropargylated core **2**.

**Scheme 5:**
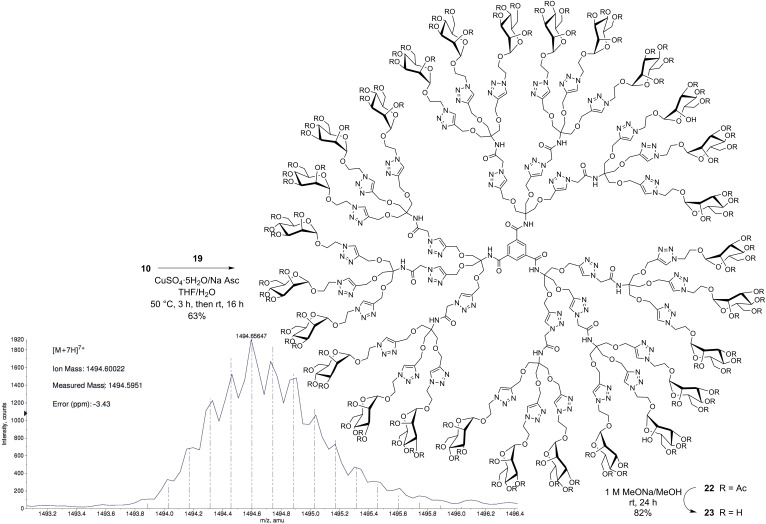
Convergent assembly of 27-mer **23** using key propargylated scaffold precursors **10** and mannosylated azidodendron **19**. Insert: Zoom section of HRMS (^+^TOF) spectrum for deprotected G(1)-mannodendrimer **23** illustrating observed and theoretical isotopic distributions for [M + 7H]^7+^ adduct.

In order to critically evaluate the subtle structural parameters imparted by glycodendrimers in deciphering their relative thermodynamic and kinetic abilities towards multivalent lectins, we designed three families of closely related mannopyranoside dendrimers. Scheme 1 describes the preparation of trimers **5** and **9** built around benzene-1,3,5-tricarboxamide (BTA or trimesamide core) having respectively nine and ten atoms between the anomeric and the benzene carbon, hence differing by a distance of only ~1.5 Å. Schemes 2–4 illustrate the syntheses of 9-mers **12** and **21** using the same trimesic acid core, together with a phloroglucinol template to initiate the synthesis of homologue **17**, but incorporating 2-amino-2-hydroxymethylpropane-1,3-diol as a branching unit (TRIS) at the G(1) level. Thus, compounds **12**, **17**, and **21** differ by having nine atoms between the anomeric carbon and the focal quaternary carbon of TRIS followed by two, four, and nine atoms to reach the benzene carbon, respectively (~4, 6, and 12 Å). Finally, the synthesis of a 27-mer mannosylated dendrimer **23** is shown in Scheme 5.

The synthesis of **5** was accomplished starting from commercial trimesic acid chloride **1** which was readily transformed into known tripropargyl amide derivative **2** [33] using propargylamine according to Scheme 1. Amide **2** was conjugated to peracetylated 2-azidoethyl α-D-mannopyranoside **3** [34] under classical copper-catalyzed dipolar cycloaddition (CuAAc) to afford **4** in 56% yield. Structure **4** was readily characterized by the absence of acetylenic protons at δ 3.16 ppm, the appearance of identical triazole protons (3H) at δ 7.74 ppm relative to the anomeric signal (3H) at δ 4.81 ppm and corresponding HRMS data. Zemplén deprotection (NaOMe, MeOH) afforded **5** in 94% yield. Synthesis of the related homolog **9**, prepared in 74% overall yield from known **6** [17] by an analogous click chemistry, is also described in Scheme 1. To this end, trichloride **1** was treated as above with 3-azido-1-propanamine to provide **6** in 87% yield. Azide–alkyne cycloaddition of **6** with prop-2-ynyl α-D-mannopyranoside **7** [35] gave **8** (79%) which was de-*O*-acetylated under Zemplén conditions (NaOMe, MeOH, 95%) to give **9**.

The syntheses of 9-mers **12**, **17** and **21** are illustrated in Schemes 2–5 and follow a conceptually identical strategy to the one described above for trimers **5** and **9**. Toward this goal, propargylated 9-mer scaffold **10** [17] was treated under the same CuAAc conditions with azide **3** to provide peracetylated **11** in 83% yield which upon Zemplén de-*O*-acetylation gave **12** in essentially quantitative yield (Scheme 2). Complete spectral characterization (^1^H, ^13^C NMR and HRMS) concluded for the aforementioned structure having twelve atoms in the linking arm (see Supporting Information File 1).

Analogously, the extended 9-mer glycodendrimer **17**, possessing fourteen atoms between the anomeric carbon and the benzene carbon, was prepared according to Scheme 3. Thus, phloroglucinol (**13**) was carefully *O*-alkylated with the previously synthesized bromoacetylated TRIS derivative **14** [36] using K_2_CO_3_ in DMF to provide **15** in 43% yield. Again, the structural integrity of **15** was fully assessed by the simplicity of its ^1^H NMR symmetrical patterns that showed the characteristic singlets for the three amide protons at δ 6.85 ppm, relative to the three benzene protons (δ 6.17 ppm) and the six *O*-acyl protons at δ 4.36 ppm (core) compared with the peripheral acetylenic methylenes (18H), inner methylene of TRIS (18H), and the terminal alkyne protons (9H) at δ 4.16, 3.87, and 2.48 ppm, respectively.

Toward the last and further extended 9-mer **21**, a convergent strategy was rather adopted (Scheme 4). This strategy has the clear advantages of providing an easier purification process from partially substituted end-products together with a better assessment of complete surface group modifications. Hence, known **14** [36] was first cycloadded to mannosylazide **3** under the above CuAAc conditions. The “click reaction” proceeded exceptionally efficiently and provided bromoacylated dendron precursor **18** in 94% yield. Substitution of the bromide by azide also proceeded uneventfully (NaN_3_, DMF, rt, 16 h) to afford intermediate glycodendron **19** in 93% yield. Finally, coupling of the propargylated core **2** with azidodendron **19** under the typical CuAAc conditions gave peracetylated intermediate **20** which was readily deprotected to give 9-mer **21** in 84% overall yield. All spectral characteristics concurred to the expected structural integrity of **21** (see Supporting Information File 1).

Finally, a 27-mer mannosylated G(1)-dendrimer **23** was similarly prepared using an accelerated convergent strategy (Scheme 5). This time, the nonapropargylated scaffold **10** was “clicked” under CuAAc with trimeric azidodendron **19** to give **22** in an acceptable yield of 63% after silica gel column chromatography, corresponding to an excellent 95% yield per individual dendron’s incorporation. The complete disappearance of propargylic signals in the ^1^H NMR spectrum supported complete conversion. Note that working with peracetylated sugar precursors allows less tedious purification practices as opposed to working with unprotected sugars which often necessitate purification by cumbersome dialysis followed by HPLC treatment. Here again, the complete structural integrity of the final product can be readily confirmed from its characteristic spectral identification. Ultimately, dendrimer **23** was deprotected under the usual Zemplén conditions in 82% yield. Once again, all the relative integrations for each proton presented on the surface were in perfect agreement with those of the middle and internal regions. Interestingly, high resolution mass spectrometry (^+^TOF technique) resulted in the formation of multicharged adducts that matched the expected theoretical patterns, especially the one corresponding to [M + 7H]^7+^, as illustrated in Scheme 5 (insert).

### NMR diffusion studies

**Figure 1:**
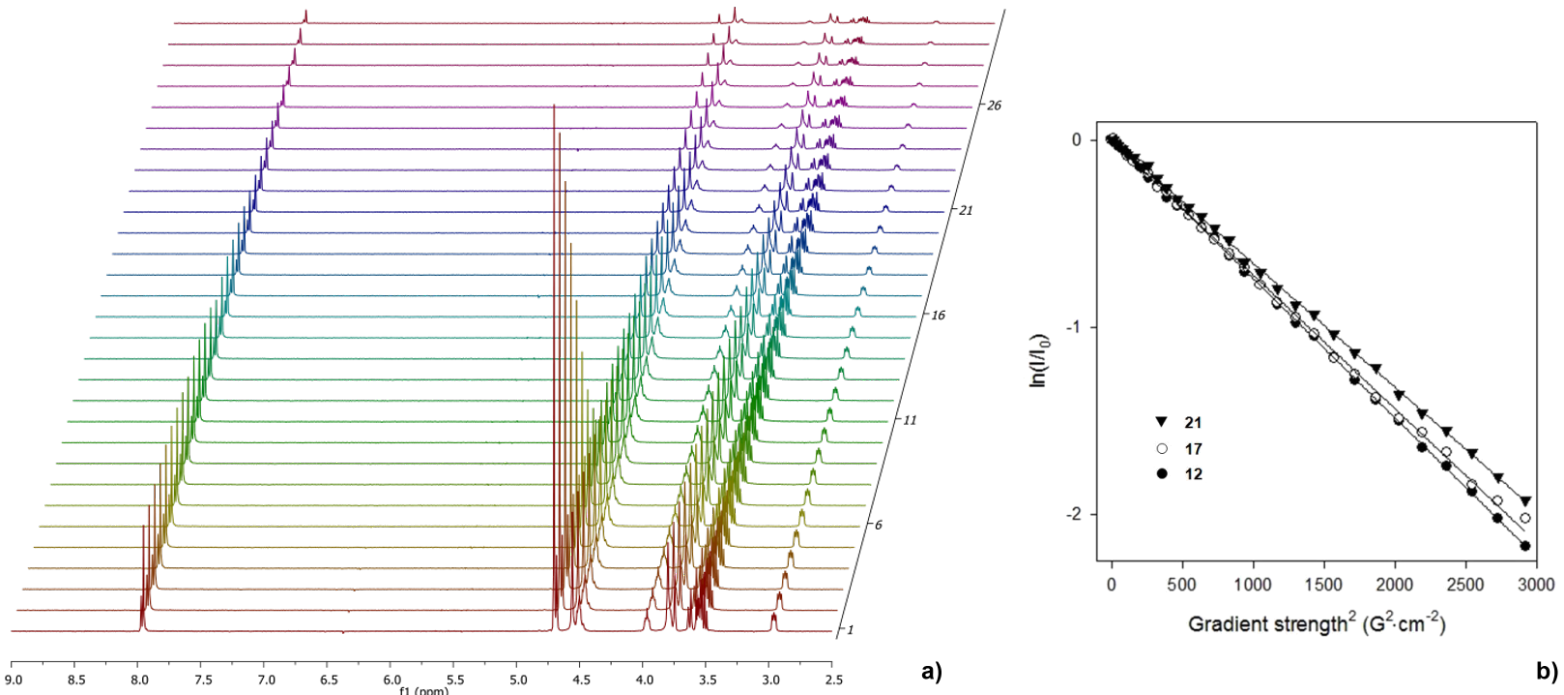
a) Decay of ^1^H signal for the nonavalent mannosylated compound **12** in D_2_O during the PFGSTE experiment. The gradient strength is increased linearly between 1.8 and 54.2 G·cm^−1^; b) characteristic echo decays of the H-5 resonances (δ = 2.98 ppm) as a function of squared gradient strength located in **12** (full circles) and **21** (full triangles) with δ = 4 ms and Δ = 50 ms (Δ = 40 ms for **17** (circles)). Notably, such linear behavior was also obtained for the decay of the signal intensities of other protons located either in internal regions of the conjugates on aromatic or branching sections, or in the peripheral saccharidic belt (results not shown).

To accurately estimate the various structural factors involved in the intricate binding interactions between our synthetic multimeric mannosides and ConA, we determined their relative diffusivity measurements by NMR spectroscopy. In fact, diffusion NMR spectroscopy has recently become a method of choice to access information about sizes and shapes of macromolecular species by measuring their diffusion coefficients in a given solvent [17,37]. The size of nonavalent compounds **12**, **17**, and **21**, and more particularly their solvodynamic radii, was thus estimated with the help of pulsed-field-gradient stimulated echo (PFG-STE) NMR experiments using bipolar pulse pairs-longitudinal-eddy-current delay (BPP-LED) in D_2_O at 25 °C. Stimulated echoes were used since they avoid signal attenuation due to transverse relaxation while bipolar gradient pulses reduce gradient artefacts [38]. The diffusion rates (*D*) were calculated from the decay of the signal intensity of the common H-5 proton (δ = 2.98 ppm) located on each epitope with increasing field gradient strength (Figure 1a). In all cases, monoexponential behavior was observed (Figure 1b), which was manifested as a linear decay of the logarithm of the signal intensity as a function of the gradient strength. This behavior is consistent with a spherical and unimolecular character of the glycodendrimers, confirming the absence of aggregation phenomena in aqueous solution under the working concentrations. The corresponding solvodynamic diameters (*d*_s_ = 2 × *r*_s_) can be calculated using the Stokes–Einstein equation and the viscosity of pure D_2_O (Table 1).

**Table 1:** Determination of diffusion data and solvodynamic diameters of nonavalent conjugates **12**, **17**, and **21** by diffusion NMR experiments.

Entry	Compound	*D* [× 10^−10^ m^2^s^−1^]^a,b,c^	Solvodynamic diameter [*d*_s_, nm]^d^

1	**12**	1.33	3.0 (2.9)
2	**17**	1.62	2.5 (2.3)
3	**21**	1.17	3.4 (2.9)

^a^See general procedures and Supporting Information File 1 for extraction of the diffusion rate and calibration of the gradient strength. *D* was determined from the decay of the H-5 resonance (δ = 2.98 ppm). ^b^Viscosity of D_2_O at 25 °C: *η*D_2_O = 1.097 × 10^−3^ Pa s. ^c^The error associated with the measurement was estimated from repeated calculations of the diffusion coefficients to be below 10%. ^d^Results in parentheses correspond to the average value calculated from the decays of 4 or 5 different proton signals.

As expected, nonavalent conjugates **12**, **17**, and **21** presented solvodynamic diameters in the range of roughly 3 nm when considering the decay of distinctive and common H-5 signals. These values remained consistent with similar congeners described earlier and harboring different epitopes [17]. The variation of the complexity of anchoring functionalities in the middle region with the incorporation of amide functions and triazole groups is responsible for a diameter enhancement for **21** when compared with **12**, as expected. On the other hand, rather counter-intuitive tendencies were observed since the apparently slightly extended structure **17** was measured as the smallest molecule of the family in water. A specific spatial arrangement of the dendrons that emanate from 1,3,5-*O*-alkylations on the aromatic core in **17**, compared to the one generated in BTAs-centered structures **12** and **21**, could explain this observation. Also, these discrepancies might result from the general amphiphilic behavior of this kind of macromolecules [39]. In fact, these glycoclusters shared common structural factors with hydrophilic peripheral moieties and an aromatic central core but the introduction of distinct functionalized linkers may change the overall hydrophobic/hydrophilic balances of the structures. As such, they could engage supplementary intramolecular hydrogen bonding or hydrophobic interactions that could mediate their three-dimensional arrangement in aqueous media. Moreover, it is also reported that the relative spatial distribution of the branches around the C=O-centered BTAs strongly depends on the nature of the substituents [40]. This hypothesis can partly explain the discrepancy observed for the calculated diameter of **21** (Table 1, entry 3). In fact, diffusion data for **21** ranged from 1.61 × 10^−10^ m^2^s^−1^ for central C*H*_ar_ to 1.17 × 10^−10^ m^2^s^−1^ for H-5, indicating a heterogeneity in diffusivity depending on the proton location within the same molecule. As a consequence, the calculated *d*_s_ value based on the utilization of an average value of diffusion data (*D*) extracted from signal decays of distinct protons located at different levels in the molecule differ from that obtained with the decay of peripheral H-5 signal only. This heterogeneity was less pronounced for **17** and absent for **12** that presented consistent values of *D* ranging from 1.51 to 1.33 × 10^−10^ m^2^s^−1^ for protons in the core or the periphery. Interestingly, calculation of the extended conformation (MM2, Chem3D) of the linkers in **12**, **17**, and **21** showed lengths of 14.8, 17.1, and 21.8 Å, respectively.

## Conclusion

The syntheses of three related families of mannosylated glycoclusters and glycodendrimers were efficiently accomplished around a benzene core and using the CuAAc methods now routinely used in this field [9,41–42]. The targeted compounds were based on trimesic acid scaffold which is known to properly expose the surface sugar groups to tetrameric lectins such as ConA [43] and the LecA lectin from *Pseudomonas aeruginosa* [17]. With these closely related families of mannosylated dendrimers in hand, together with their known relative size in solution, we are now well positioned to evaluate their binding behavior against their cognate proteins and this work will be published in due course [44].

The study of subtle structural variations and the nature of anchoring functions, as observed in diffusivity experiments, could represent a first step towards rational interpretation to explain the differential kinetic behavior within a closely related family of glycoclusters.

## Experimental

### General remarks

All reactions in organic medium were performed in standard oven-dried glassware under an inert atmosphere of nitrogen using freshly distilled solvents. CH_2_Cl_2_ was distilled from CaH_2_ and DMF from ninhydrin, and kept over molecular sieves. Solvents and reagents were deoxygenated when necessary by purging with nitrogen. Water used for lyophilization of final dendrimers was nanopure grade, purified through Barnstead NANOPure II Filter with Barnstead MegOhm-CM Sybron meter. All reagents were used as supplied without prior purification unless otherwise stated, and obtained from Sigma-Aldrich Chemical Co. Ltd. Reactions were monitored by analytical thin-layer chromatography using silica gel 60 F254 precoated plates (E. Merck) and compounds were visualized by 254 nm light, a mixture of iodine/silica gel and/or mixture of ceric ammonium molybdate solution (100 mL H_2_SO_4_, 900 mL H_2_O, 25 g (NH_4_)_6_Mo_7_O_24_H_2_O, 10 g Ce(SO_4_)_2_) and subsequent development by gentle warming with a heat-gun. Purifications were performed by flash column chromatography using silica gel from Silicycle (60 Å, 40–63 µm) with the indicated eluent.

### NMR, IR, and MS spectroscopy

^1^H NMR and ^13^C NMR spectra were recorded at 300 or 600 MHz and 75 or 150 MHz, respectively, on a Bruker spectrometer (300 MHz) and Varian spectrometer (600 MHz). All NMR spectra were measured at 25 °C in indicated deuterated solvents. Proton and carbon chemical shifts (δ) are reported in ppm and coupling constants (*J*) are reported in Hertz (Hz). The resonance multiplicity in the ^1^H NMR spectra are described as “s” (singlet), “d” (doublet), “t” (triplet), and “m” (multiplet) and broad resonances are indicated by “br”. Residual protic solvent of CDCl_3_ (^1^H, δ 7.27 ppm; ^13^C, δ 77.0 ppm (central resonance of the triplet)), D_2_O (^1^H, δ 4.79 ppm and 30.89 ppm for CH_3_ of acetone for ^13^C spectra of de-*O*-acetylated compounds), MeOD (^1^H, δ 3.31 ppm and ^13^C, δ 49.0 ppm). 2D Homonuclear correlation ^1^H-^1^H COSY together with 2D heteronuclear correlation ^1^H-^13^C HSQC experiments were used to confirm NMR peak assignments.

Fourier transform infrared (FTIR) spectra were obtained with Thermo-scientific, Nicolet model 6700 equipped with ATR. The absorptions are given in wavenumbers (cm^−1^). The intensity of the bands is described as s (strong), m (medium) or w (weak). Melting points were measured on a Electrothermal MEL-TEMP apparatus and are uncorrected.

Accurate mass measurements (HRMS) were performed on a LC–MSD–ToF instrument from Agilent Technologies in positive electrospray mode. Low-resolution mass spectra were performed on the same apparatus or on a LCQ Advantage ion trap instrument from Thermo Fisher Scientific in positive electrospray mode (Mass Spectrometry Laboratory (Université de Montréal), or Plateforme analytique pour molécules organiques (Université du Québec à Montréal), Québec, Canada). Either protonated molecular ions [M + *n*H]*^n^*^+^ or adducts [M + *n*X]*^n^*^+^ (X = Na, K, NH_4_) were used for empirical formula confirmation.

NMR diffusion measurements were performed at 25 °C on a Varian Inova Unity 600 spectrometer (Varian, Walnut Creek, CA, USA) operating at a frequency of 599.95 MHz for ^1^H using a 5 mm broadband *z*-gradient temperature-regulated probe. The temperature was calibrated with 1,2-ethanediol according to a standard procedure [38]. The diffusion experiment employed a bipolar pulse-field gradients stimulated echo sequence as proposed by Wu et al [45]. The gradient pulse duration δ was 4 ms and the diffusion times (Δ) were 40 to 50 ms to ensure that the echo intensities were attenuated by at least 80%. A complete attenuation curve was obtained by measuring 30 gradient strengths, which were linearly incremented between 1.8 and 54.2 Gcm^−1^. Hard 90° ^1^H pulses of 15 μs were used and 36 k data points were recorded with 16 scans acquired for each gradient’s strength. A recycle delay of 3.0 s was used. The gradient strength was calibrated by back calculation of the coil constant from diffusion experiments on H_2_O traces in D_2_O (*D* = 1.90 × 10^−9^ m^2^ s^−1^) [46].

Diffusion rates were extracted from the slope of the straight lines obtained by plotting ln(*I*) against the gradient-pulse power squared according to the following equation: ln(*I*) *= −D*γ^2^*G*^2^δ^2^(Δ − δ/3 − τ/2) *+* ln*(I*_0_*)* where *I* is the relative intensity of a chosen resonance (*I* = *I*_0_exp*−*[*D*γ^2^*G*^2^δ^2^(Δ − δ/3 − τ/2)]), *G* = gradient strength (*T*/m), γ = proton gyromagnetic ratio, *D* = diffusion rate (m^2^ s^−1^), δ = gradient duration, Δ = diffusion delay, and τ = pulse length for bipolar pulses. All diffusion spectra were processed in Mat NMR [47].

### Glycodendrimer synthesis

#### Procedure A: multiple CuAAc couplings on polypropargylated cores

To a solution of polypropargylated core (1.00 equiv) and complementary azido synthon (1.25 equiv/propargyl) in a THF/H_2_O mixture (1:1) were added sodium ascorbate (0.30 equiv/propargyl) and CuSO_4_·5H_2_O (0.30 equiv/propargyl). The reaction mixture was stirred at 50 °C for 3 h then at room temperature for an additional 16 h period. Ethyl acetate (10 mL) was added and the resulting solution was poured in a separatory funnel containing 25 mL of EtOAc and 30 mL of a saturated aqueous solution of NH_4_Cl. Organics were washed with (2 × 25 mL) of saturated NH_4_Cl_aq_, water (2 × 20 mL) and brine (1 × 10 mL). The organic phase was then dried over MgSO_4_ and concentrated under reduced pressure. Column chromatography on silica (DCM/MeOH 100:0 to 90:10) afforded the desired glycocluster.

#### Procedure B: Zemplén de-*O*-acetylation procedure for insoluble hydroxylated derivatives

The acetylated compound was dissolved in anhydrous MeOH and a solution of sodium methoxide (1 M in MeOH, 5 µL every 20 min until precipitation) was added. An additional 100 µL was then injected and the heterogeneous reaction mixture was stirred at room temperature for 24 h. The solvent was then removed with a Pasteur pipette and a mixture of anhydrous MeOH/DCM (4:1, 5 mL) was added to the residual white foam. A vigorous agitation is maintained for an additional 15 min period. After removal of the solvents with a Pasteur pipette, the residue was dissolved in H_2_O (3 mL), and the pH was adjusted to 7 by the addition of ion-exchange resin (Amberlite IR 120 H^+^). After filtration, the solvent was removed under vacuum with a rotary evaporator and lyophilized to yield the fully deprotected glycocluster.

**Synthesis of peracetylated trivalent derivative 8:** To a solution of triazido core **6** (50.0 mg, 109 μmol, 1.00 equiv) and mannoside **7** (158 mg, 409 μmol, 3.75 equiv) in a THF/H_2_O mixture (1:1, 6 mL) were added sodium ascorbate (19.4 mg, 98.1 μmol, 0.90 equiv) and CuSO_4_·5H_2_O (24.5 mg, 98.1 μmol, 0.90 equiv). The reaction mixture was stirred at 50 °C for 3 h then at room temperature for an additional 16 h period. Ethyl acetate (10 mL) was added and the resulting solution was poured in a separatory funnel containing 35 mL of EtOAc and 30 mL of a saturated aqueous solution of NH_4_Cl. Organics were washed with (2 × 25 mL) of saturated NH_4_Cl_aq_, water (2 × 20 mL) and brine (1 × 10 mL). The organic phase was then dried over MgSO_4_ and concentrated under reduced pressure. Column chromatography on silica (DCM/MeOH 98:2 to 94:6) afforded the desired compound **8** (138 mg, 86.0 μmol, 79%) as a viscous oil. *R*_f_ 0.34 (95:5 DCM/MeOH); ^1^H NMR (600 MHz, CDCl_3_) δ (ppm) 8.27 (s, 3H, C*H*_ar_), 7.79 (s, 3H, C*H*_triazole_), 7.72 (t, *J* = 5.3 Hz, 3H, N*H*), 5.29–5.19 (m, 9H, *H*_2_, *H*_3_, *H*_4_), 4.92 (s_app_, 3H, *H*_1_), 4.77–4.62 (2 × d, *J* = 12.4 Hz, 6H, OC*H*_2_), 4.54 (t, *J* = 6.4 Hz, 6H, N_triazole_C*H*_2_), 4.28 (dd, *J* = 12.4 Hz, *J* = 5.4 Hz, 3H, *H*_6b_), 4.11–4.03 (m, 6H, *H*_5_ + *H*_6a_), 3.55 (m, 6H, NHC*H*_2_), 2.28 (m, 6H, CH_2_C*H*_2_CH_2_), 2.12, 2.10, 2.02, 1.96 (4s, 36H, COC*H*_3_); ^13^C{^1^H} NMR (150 MHz, CDCl_3_) δ (ppm) 170.8, 170.1, 170.0, 169.7 (*C*OCH_3_), 166.1 (*C*ONH), 143.5 (*C*_triazole_), 134.9 (*C*_arom_), 128.5 (*C*H_arom_), 123.9 (*C*H_triazole_), 96.7 (*C*_1_), 69.3 (*C*_2_), 69.0 (*C*_3_), 68.7 (*C*_5_), 65.9 (*C*_6_), 62.3 (*C*_4_), 60.7 (O*C*H_2_), 48.3 (*C*H_2_N_triazole_), 37.5 (NH*C*H_2_), 29.9 (CH_2_*C*H_2_CH_2_), 20.9, 20.8, 20.7, 20.7 (CO*C*H_3_); MS (^+^TOF-MS, *m*/*z*): [M + H]^+^ calculated for C_69_H_90_N_12_O_33_, 1615.6; found, 1615.6.

**Synthesis of nonapropargylated core 15:** To a solution of phloroglucinol (**13**, 10.0 mg, 79.3 μmol, 1.00 equiv) in anhydrous DMF (3 mL) was added under nitrogen anhydrous K_2_CO_3_ (previously heated at 250 °C under vaccum, 39.5 mg, 285 μmol, 3.60 equiv). After 10 min of vigorous stirring, tripropargylated synthon **14** (93.0 mg, 285 μmol, 3.60 equiv) was added into the solution under inert atmosphere and the reaction mixture was allowed to stir at 65 °C for 39 h. In the end, the dark-brown heterogeneous reaction was poured in 30 mL of EtOAc and organics were washed with a saturated aqueous solution of NH_4_Cl (2 × 30 mL) then water (2 × 20 mL) and brine (10 mL). The organic phase was then dried over MgSO_4_ and concentrated under reduced pressure. Column chromatography on silica (EtOAc/hexane 40:60 to 50:50) afforded the desired compound **15** (32.0 mg, 33.8 μmol, 43%) as a colorless oil. *R*_f_ 0.27 (1:1 EtOAc/hexane); ^1^H NMR (300 MHz, CDCl_3_) δ (ppm) 6.85 (s, 3H, N*H*), 6.17 (s, 3H, C*H*_ar_), 4.36 (s, 6H, OC*H*_2_CONH), 4.16 (m, 18H, OC*H*_2_C≡CH), 3.87 (br s, 18H, HNC_q_C*H*_2_O), 2.48 (m, 9H, OCH_2_C≡C*H*); ^13^C{^1^H} NMR (75 MHz, CDCl_3_) δ (ppm) 167.3 (*C*ONH), 159.0 (*C*_ar_OCH_2_), 95.8 (*C*H_ar_), 79.4 (OCH_2_*C*≡CH), 74.9 (OCH_2_C≡*C*H), 68.3 (HNC_q_*C*H_2_O), 67.5 (O*C*H_2_CONH), 59.2 (*C*_q_), 58.6 (O*C*H_2_C≡CH); HRMS (^+^TOF-HRMS, *m*/*z*): [M + H]^+^ calculated for C_51_H_57_N_3_O_15_, 952.3862; found, 952.3843 (Δ = −2.10 ppm); [M + Na]^+^: calculated for 974.3682; found, 974.3662 (Δ = −2.05 ppm).

**Synthesis of bromoacylated dendron 18:** To a solution of tripropargylated synthon **14** (140.0 mg, 393.0 μmol, 1.00 equiv) and mannoside **3** (616 mg, 1.48 mmol, 3.75 equiv) in a THF/H_2_O mixture (1:1, 6 mL) were added sodium ascorbate (70.0 mg, 354 μmol, 0.90 equiv) and CuSO_4_·5H_2_O (88.4 mg, 354 μmol, 0.90 equiv). The reaction mixture was stirred at 50 °C for 3 h then at room temperature for an additional 16 h period. Ethyl acetate (20 mL) was added and the resulting solution was poured in a separatory funnel containing 40 mL of EtOAc and 30 mL of a saturated aqueous solution of NH_4_Cl. Organics were washed with 2 × 35 mL of saturated NH_4_Cl_aq_, water (2 × 30 mL) and brine (20 mL). The organic phase was then dried over MgSO_4_ and concentrated under reduced pressure. Column chromatography on silica (DCM/MeOH 99:1 to 96:4) afforded the desired compound **18** (594 mg, 369.4 μmol, 94%) as a white solid. *R*_f_ 0.47 (94:6 DCM/MeOH); mp 68–72 °C (not corrected); ^1^H NMR (300 MHz, CDCl_3_) δ (ppm) 7.68 (br s, 3H, C*H*_triazole_), 6.89 (br s, 1H, N*H*), 5.24–5.18 (m, 9H, *H*_2_, *H*_3_, *H*_4_), 4.80 (d, *J* = 1.3 Hz, 1H, *H*_1_), 4.61–4.58 (br s, 12H, OC*H*_2_C_triazole_ + N_triazole_C*H*_2_), 4.17–4.00 (m, 11H, OC*H*_2_CH_2_ + *H*_6a_ + BrC*H*_2_CONH), 3.94–3.78 (m, 9H, *H*_6b_ + NHC_q_C*H*_2_O), 3.60 (m, 3H, *H*_5_), 2.12, 2.08, 2.03, 1.98 (4s, 36H, COC*H*_3_); ^13^C{^1^H} NMR (75 MHz, CDCl_3_) δ (ppm) 170.5, 169.9, 169.9, 169.5, (*C*OCH_3_), 165.6 (*C*ONH), 145.0 (*C*_triazole_), 123.7 (*C*H_triazole_), 97.4 (*C*_1_), 69.1 (*C*_2_), 68.9 (*C*_3_), 68.8 (*C*_5_), 68.4 (NHC_q_*C*H_2_O), 66.2 (*C*_6_), 65.6 (*C*_4_), 64.6 (O*C*H_2_C_triazole_), 62.1 (O*C*H_2_CH_2_), 60.2 (*C*_q_), 49.6 (*C*H_2_N_triazole_), 29.7 (*C*H_2_Br), 20.8, 20.7, 20.6, 20.6 (CO*C*H_3_); IR (cm^−1^): 2956, 2937, 2361, 2337, 1751, 1734, 1540, 1370, 1218, 1045, 759; HRMS (^+^TOF-HRMS, *m*/*z*): [M + 2H]^2+^ calculated for C_63_H_87_BrN_10_O_34_, 804.2358; found, 804.2356 (Δ = −0.18 ppm); [M + H] ^+^ calculated for 1607.4642, found: 1607.4620 (Δ = −1.36 ppm); [M + Na]^+^ calculated for 1629.4462; found, 1629.4448 (Δ = −0.84 ppm).

**Synthesis of azidoacylated dendron 19:** To a stirring solution of brominated trivalent dendron **18** (121.0 mg, 75.2 μmol, 1.00 equiv) in dry DMF (1.5 mL) was added under a nitrogen atmosphere sodium azide (7.3 mg, 112 μmol, 1.50 equiv). After stirring overnight at room temperature, the solvent was removed under vaccum. Ethyl acetate (20 mL) was added and the resulting solution was poured in a separatory funnel containing 20 mL of EtOAc and 30 mL of a saturated aqueous solution of NH_4_Cl. Organics were washed with 2 × 30 mL of saturated NH_4_Cl_aq_, water (2 × 30 mL) and brine (20 mL). The organic phase was then dried over MgSO_4_ and concentrated under reduced pressure to furnish the desired compound **19** (110 mg, 69.9 μmol, 93%) as a white solid. *R*_f_ 0.47 (94:6 DCM/MeOH); mp 62–65 °C (not corrected); ^1^H NMR (300 MHz, CDCl_3_) δ (ppm) 7.68 (br s, 3H, C*H*_triazole_), 6.69 (br s, 1H, N*H*), 5.27–5.18 (m, 9H, *H*_2_, *H*_3_, *H*_4_), 4.80 (d, *J* = 1.3 Hz, 1H, *H*_1_), 4.61–4.58 (br s, 12H, OC*H*_2_C_triazole_ + N_triazole_C*H*_2_), 4.23–4.00 (m, 11H, OC*H*_2_CH_2_ + *H*_6a_ + N_3_C*H*_2_CONH), 3.90–3.81 (m, 9H, *H*_6b_ + NHC_q_C*H*_2_O), 3.60 (m, 3H, *H*_5_), 2.12, 2.08, 2.03, 1.98 (4s, 36H, COC*H*_3_); ^13^C{^1^H} NMR (75 MHz, CDCl_3_) δ (ppm) 170.4, 169.9, 169.8, 169.5, (*C*OCH_3_), 166.7 (*C*ONH), 144.9 (*C*_triazole_), 123.7 (*C*H_triazole_), 97.4 (*C*_1_), 69.0 (*C*_2_), 68.8 (*C*_3_), 68.8 (*C*_5_), 68.4 (NHC_q_*C*H_2_O), 66.1 (*C*_6_), 65.6 (*C*_4_), 64.5 (O*C*H_2_C_triazole_), 62.1 (O*C*H_2_CH_2_), 59.9 (*C*_q_), 52.5 (*C*H_2_N_3_), 49.5 (*C*H_2_N_triazole_), 20.7, 20.7, 20.6, 20.6 (CO*C*H_3_); IR (cm^−1^): 2934, 2361, 2338, 2107 (N_3_), 1751, 1734, 1540, 1373, 1218, 1045, 761; HRMS (^+^TOF-HRMS, *m*/*z*): [M + H]^+^ calculated for C_63_H_87_N_13_O_34_, 1570.5551; found, 1570.5543 (Δ = −0.51 ppm); [M + Na]^+^ calculated for 1592.5371; found, 1592.5366 (Δ = −0.31 ppm).

**Synthesis of peracetylated 27-mer derivative 22:** To a solution of nonapropargylated core **10** (4.6 mg, 5.38 μmol, 1.00 equiv) and trimannosylated dendron **19** (95.0 mg, 60.5 μmol, 11.25 equiv) in a THF/H_2_O mixture (1:1, 3 mL) were added sodium ascorbate (2.9 mg, 15 μmol, 2.70 equiv) and CuSO_4_·5H_2_O (3.6 mg, 15 μmol, 0.90 equiv). The reaction mixture was stirred at 50 °C for 3 h then at room temperature for an additional 16 h period. Ethyl acetate (10 mL) was added and the resulting solution was poured in a separatory funnel containing 25 mL of EtOAc and 30 mL of a saturated aqueous solution of NH_4_Cl. Organics were washed with 2 × 25 mL of saturated NH_4_Cl_aq_, water (2 × 20 mL) and brine (10 mL). The organic phase was then dried over MgSO_4_ and concentrated under reduced pressure. Column chromatography on silica (DCM/MeOH 98:2 to 90:10) afforded the desired compound **22** (50.0 mg, 3.33 μmol, 63%) as a yellowish oil. *R*_f_ 0.72 (90:10 DCM/MeOH); ^1^H NMR (600 MHz, CDCl_3_) δ (ppm) 8.27 (m, 3H, C*H*_ar_), 7.79 (s, 9H, C*H*_int-triazole_), 7.75 (s, 27H, C*H*_ext-triazole_), 7.34–7.31 (m, 12H, N*H*), 5.23–5.18 (m, 81H, *H*_2_, *H*_3_, *H*_4_), 5.05 (br s, 18H, N_triazole_C*H*_2_CONH), 4.81 (s_app_, 27H, *H*_1_), 4.62–4.53 (m, 126H, OC*H*_2_C_triazole_ + N_triazole_C*H*_2_), 4.20–3.64 (m, 207H, OC*H*_2_ + *H*_6_ + NHC_q_C*H*_2_O + *H*_5_), 2.11, 2.08, 2.01, 1.96 (4s, 324H, COC*H*_3_); ^13^C{^1^H} NMR (150 MHz, CDCl_3_) δ (ppm) 170.6, 170.5, 170.0, 169.9, 169.9, 169.7, 169.6 (*C*OCH_3_), 168.4 (*C*ONH), 165.4 (*C*ONH), 144.9 + 144.8 (*C*_ext-triazole_), 144.5 (*C*_int-triazole_), 135.6 (*C*_arom_), 128.6 (*C*H_arom_), 124.9 (*C*H_int-triazole_), 124.0 (*C*H_ext-triazole_), 97.5 (*C*_1_), 69.1 (*C*_2_), 69.0 (*C*_3_), 68.7 (*C*_5_), 68.4 (NHC_q_*C*H_2_O), 66.2 (*C*_6_), 65.6 (*C*_4_), 64.5 (O*C*H_2_C_triazole_), 62.1 (O*C*H_2_), 60.4 (*C*_q_), 52.4 (N_triazole_C*H*_2_CONH), 49.5 (*C*H_2_N_triazole_), 20.8, 20.8, 20.7, 20.7 (CO*C*H_3_); MS (^+^TOF-MS, *m*/*z*): [M + H]^+^ calculated for C_615_H_834_N_120_O_318_, 14995.8; found, 14995.9.

**Synthesis of de-*O*-acetylated 27-mer derivative 23:** Acetylated compound **22** (30.0 mg, 2.00 μmol) was dissolved in anhydrous MeOH (3 mL) and a solution of sodium methoxide (1 M in MeOH, 5 µL every 20 min until precipitation) was added. An additional 100 µL was then injected and the heterogeneous reaction mixture was stirred at room temperature for 24 h. The solvent was then removed with a Pasteur pipette and a mixture of anhydrous MeOH/DCM (4:1, 5 mL) was added to the residual white foam. A vigorous agitation is maintained for an additional 15 min period. After removal of the solvent with a Pasteur pipette, the residue was dissolved in 3 mL of H_2_O, and the pH was adjusted to 7 with addition of ion-exchange resin (Amberlite IR 120 H^+^). After filtration, the solvent was removed under vacuum with a rotary evaporator and lyophilized to yield the fully deprotected 27-mer **23** as a white solid (17.0 mg, 1.63 μmol) in 82% yield. ^1^H NMR (600 MHz, D_2_O) δ (ppm) 8.06 (m, 3H, C*H*_ar_), 7.97 (s, 27H, C*H*_ext-triazole_), 7.96 (s, 9H, C*H*_int-triazole_), 5.14 (br s, 18H, N_triazole_C*H*_2_CONH), 4.75 (s, 27H, *H*_1_), 4.59−4.51 (m, 126H, OC*H*_2_C_triazole_ + N_triazole_C*H*_2_), 4.05–4.03 (m, 27H, OCH*H*CH_2_N), 3.83–3.80 (m, 72H, OC*H*HCH_2_N + *H*_2_ + NHC_q_C*H*_2_O_int_), 3.71–3.57 (m, 162H, NHC_q_C*H*_2_O_ext_ + *H*_6_ + *H*_4_ + *H*_3_), 3.01 (m, 27H, *H*_5_); ^13^C{^1^H} NMR (150 MHz, D_2_O) δ (ppm) 168.8 (*C*ONH_int_), 167.5 (*C*ONH_ext_), 144.7 (*C*_ext-triazole_), 144.6 (*C*_int-triazole_), 135.7 (*C*_arom_), 129.7 (*C*H_arom_), 127.0 (*C*H_int-triazole_), 126.1 (*C*H_ext-triazole_), 100.2 (*C*_1_), 73.5 (*C*_5_), 71.1 (*C*_3_), 70.6 (*C*_2_), 68.2 (NHC_q_*C*H_2_O), 68.0 (NHC_q_*C*H_2_O), 67.0 (O*C*H_2_CH_2_N_triazole_), 66.1 (*C*_4_), 64.2 (O*C*H_2_C_triazole_), 61.3 (*C*_6_), 60.9 (*C*_q_), 52.9 (N_triazole_C*H*_2_CONH), 50.7 (*C*H_2_N_triazole_), 35.7 (OCHN*C*H_2_C_triazole_); HRMS (^+^TOF-HRMS, *m*/*z*): [M + 7H]^7+^ calculated for C_399_H_204_N_120_O_210_, 1494.6002; found, 1494.5951 (Δ = −3.43 ppm).

## Supporting Information

File 1 Experimental procedures, characterization data, NMR, IR and mass spectra and NMR diffusion experiments.
