# Supplementary material for: Synthesis and solvodynamic diameter measurements of closely related mannodendrimers for the study of multivalent carbohydrate–protein interactions
Source: Beilstein J Org Chem. 2014 Jul 4;10:1524–35. doi: 10.3762/bjoc.10.157 (PMC4144466; doi:10.3762/bjoc.10.157)
Supplement: File 1 — Experimental procedures, characterization data, NMR, IR and mass spectra and NMR diffusion experiments. [file Beilstein_J_Org_Chem-10-1524-s001.pdf]

Supporting Information

for

Synthesis and solvodynamic diameter measurements of closely related mannodendrimers for the study of multivalent carbohydrate–protein interactions

Yoann M. Chabre, Alex Papadopoulos, Alexandre A. Arnold and René Roy*

Address: Pharmaqam, Department of Chemistry, Université du Québec à Montréal, P. O. Box 8888, Succ. Centre-ville, Montréal, Québec, Canada H3C 3P8

Email: René Roy* - roy.rene@uqam.ca

*Corresponding author

Experimental procedures, characterization data, NMR, IR and mass spectra and NMR diffusion experiments

Contents

1. Experimental procedures and data S2
2. NMR, IR, and mass spectra S11
3. NMR diffusion experiments S49

Experimental procedures and data

All reactions in organic medium were performed in standard oven-dried glassware under an inert atmosphere of nitrogen using freshly distilled solvents. CH_2Cl_2 was distilled from CaH_2 and DMF from ninhydrin, and kept over molecular sieves. Solvents and reagents were deoxygenated when necessary by purging with nitrogen. Water used for lyophilization of final dendrimers was nanopure grade, purified through Barnstead NANOPure II Filter with Barnstead MegOhm-CM Sybron meter. All reagents were used as supplied without prior purification unless otherwise stated, and obtained from Sigma–Aldrich Chemical Co. Ltd. Reactions were monitored by analytical thin layer chromatography using silica gel 60 F254 precoated plates (E. Merck) and compounds were visualized by 254 nm light, a mixture of iodine/silica gel and/or mixture of ceric ammonium molybdate solution (100 mL H_2SO_4, 900 mL H_2O, 25 g $(NH_4)_6Mo_7O_{24}H_2O$, 10 g $Ce(SO_4)_2$) and subsequent development by gentle warming with a heat-gun. Purifications were performed by flash column chromatography using silica gel from Silicycle (60 Å, 40–63 µm) with the indicated eluent.

NMR, IR spectroscopies and MS spectrometry

^{1}H NMR and ^{13}C NMR spectra were recorded at 300 or 600 MHz and 75 or 150 MHz, respectively, on a Bruker spectrometer (300 MHz) and Varian spectrometer (600 MHz). All NMR spectra were measured at 25 °C in indicated deuterated solvents. Proton and carbon chemical shifts (δ) are reported in ppm and coupling constants (*J*) are reported in Hertz (Hz). The resonance multiplicity in the ^{1}H NMR spectra are described as “s” (singlet), “d” (doublet), “t” (triplet), and “m” (multiplet) and broad resonances are indicated by “br”. Residual protic solvent of $CDCl_3$ (^{1}H, δ 7.27 ppm; ^{13}C, δ 77.0 ppm (central resonance of the triplet)), D_2O (^{1}H, δ 4.79 ppm and 30.89 ppm for CH_3 of acetone for ^{13}C spectra of de-*O*-acetylated compounds), MeOD (^{1}H, δ 3.31 ppm and ^{13}C, δ 49.0 ppm. 2D Homonuclear correlation ^{1}H-^{1}H COSY together with 2D heteronuclear correlation ^{1}H-^{13}C HSQC experiments were used to confirm NMR peak assignments.

Fourier transform infrared (FTIR) spectra were obtained with Thermo-scientific, Nicolet model 6700 equipped with ATR. The absorptions are given in wavenumbers (cm^{-1}). The intensity of the bands is described as s (strong), m (medium) or w (weak). Melting points were measured on an Electrothermal MEL-TEMP apparatus and are uncorrected.

Accurate mass measurements (HRMS) were performed on a LC–MSD-ToF instrument from Agilent Technologies in positive electrospray (ES) mode. Low-resolution mass spectra were performed on the same apparatus or on a LCQ Advantage ion trap instrument from Thermo Fisher Scientific in positive electrospray mode (Mass Spectrometry Laboratory (Université de Montréal), or Plateforme analytique pour molécules organiques (Université du Québec à Montréal), Québec, Canada). Either protonated molecular ions $[M + nH]^{n+}$ or adducts $[M + nX]^{n+}$ (X = Na, K, NH_4) were used for empirical formula confirmation.

General procedures

Procedure A: multiple CuAAc couplings on polypropargylated cores

To a solution of polypropargylated core (1.00 equiv) and complementary azido synthon (1.25 equiv/propargyl) in a THF/H_2O mixture (1:1) were added sodium ascorbate (0.30 equiv/propargyl) and $CuSO_4 \cdot 5H_2O$ (0.30 equiv/propargyl). The reaction mixture was stirred at 50 °C for 3 h then at room temperature for an additional 16 h period. EtOAc (10 mL) was added and the resulting solution was poured in a separatory funnel containing 25 mL of EtOAc and 30 mL of a saturated aqueous solution of NH_4Cl. Organics were washed with (2 × 25 mL) of saturated NH_4Cl_{aq}, water (2 × 20 mL) and brine (10 mL). The organic phase was then dried over $MgSO_4$ and concentrated under reduced pressure. Column chromatography on silica (DCM/MeOH 100:0 to 90:10) afforded the desired glycocluster.

Procedure B: Zemplén de-*O*-acetylation procedure for insoluble derivatives

The acetylated compound was dissolved in anhydrous MeOH and a solution of sodium methoxide (1 M in MeOH, 5 µL every 20 minutes until precipitation) was added. An additional 100 µL was then injected and the heterogeneous reaction mixture was stirred at room temperature for 24 h. The solvent was then removed with a Pasteur pipette and a mixture of anhydrous MeOH/DCM (4:1, 5 mL) was added to the residual white foam. A vigorous agitation was maintained for an additional 15 min period. After removal of the solvents with a Pasteur pipette, the residue was dissolved in H_2O (3 mL), and the pH was adjusted to 6–7 with addition of ion-exchange resin (Amberlite IR 120 H^+). After filtration, the solvent was removed under vacuum with rotary evaporator, lyophilized to yield the fully deprotected glycocluster.

Synthesis of peracetylated trivalent derivative (4): Derivative **4** was synthesized according to **Procedure A** with tripropargylated core **2** (15.4 mg, 47.9 µmol, 1.00 equiv), mannoside **3** (75.0 mg, 180 µmol, 3.75 equiv), sodium ascorbate (8.5 mg, 43 µmol, 0.90 equiv) and $CuSO_4 \cdot 5H_2O$ (10.8 mg, 43.1 µmol, 0.90 equiv) in a THF/H_2O mixture (4 mL, 1:1). Column chromatography on silica (DCM/MeOH 98:2 to 94:6) afforded the desired compound **4** (42.0 mg, 26.8 µmol, 56%) as a white solid.

R_f = 0.16 (94:6 DCM/MeOH); **m.p.** = 103-106°C (*not corrected*). **^{1}H NMR (300 MHz, $CDCl_3$)** δ (ppm) 8.21 (m, 6H, CH_{ar} + N*H*), 7.74 (s, 3H, C$H_{triazole}$), 5.23−5.17 (m, 9H, H_2, H_3, H_4), 4.81 (s_{app}, 3H, H_1), 4.67−4.60 (m, 12H, HNC$H_2C_{triazole}$ + $N_{triazole}$CH_2), 4.21−3.89 (m, 12H, OCH_2 + H_6), 3.62−3.60 (m, 3H, H_5), 2.11, 2.08, 2.01, 1.96 (4s, 36H, COCH_3). **^{13}C{^{1}H} NMR (75 MHz, $CDCl_3$)** δ (ppm) 170.7, 170.1, 170.0, 169.7 (*C*OCH$_3$), 165.9 (*C*ONH), 145.0 ($C_{triazole}$), 134.6 (C_{arom}), 128.5 (CH_{arom}), 123.4 ($CH_{triazole}$), 97.3 (C_1), 69.2 (C_2), 68.9 (C_3), 68.8 (C_5), 66.1 (C_6), 65.7 (C_4), 62.2 (O*C*H$_2$), 49.7 ($CH_2N_{triazole}$), 35.5 (HN*C*H$_2C_{triazole}$), 20.8, 20.7, 20.6, 20.6 (CO*C*H$_3$). **HRMS** ($^+$TOF-HRMS) m/z: calculated for $C_{66}H_{84}N_{12}O_{33}$ $[M+H]^+$: 1573.5337, found: 1573.5327 (Δ = −0.88 ppm); $[M+Na]^+$: 1595.5156, found: 1595.5151 (Δ = −0.29 ppm).

Synthesis of trivalent derivative (5): A solution of sodium methoxide (1 M in MeOH, 50 µL) was added to a solution of acetylated precursor **4** (30.0 mg, 19.1 µmol) in MeOH (2.5 mL). The reaction mixture was stirred at room temperature for 24 h. The pH was adjusted to 6–7 with addition of ion-exchange resin (Amberlite IR 120 H^+). After filtration, the solvent was removed under vacuum with rotary evaporator and the residue was lyophilized to furnish desired de-*O*-acetylated compound **5** (19.0 mg, 17.9 µmol, 94%) as a white solid.

^{1}H NMR (300 MHz, D_2O) δ (ppm) 8.27 (s, 3H, CH_{ar}), 8.01 (s, 3H, $CH_{triazole}$), 4.74 (m, 3H, H_1), 4.63−4.59 (m, 12H, $HNCH_2C_{triazole}$+ $N_{triazole}CH_2$), 4.05−4.01 (m, 3H, $CHHCH_2N$), 3.88−3.79 (m, 6H, $CHHCH_2N$ + H_2), 3.63−3.47 (m, 12H, H_6 + H_4 + H_3), 2.90−2.86 (m, 3H, H_5). **^{13}C{^{1}H} NMR (75 MHz, D_2O)** δ (ppm) 168.4 (*C*ONH), 144.6 ($C_{triazole}$), 134.5 (C_{arom}), 129.3 (CH_{arom}), 124.7 ($CH_{triazole}$), 99.5 (C_1), 72.8 (C_5), 70.5 (C_3), 69.9 (C_2), 66.4 (C_4), 65.5 (OCH_2), 60.7 (C_6), 50.1 ($CH_2N_{triazole}$), 35.1 ($HNCH_2C_{triazole}$). **HRMS** ($^+$TOF-HRMS) m/z: calculated for $C_{42}H_{60}N_{12}O_{21}$ $[M+Na]^+$: 1091.3888, found: 1091.3888 (Δ = −0.11 ppm).

Synthesis of peracetylated trivalent derivative (8): To a solution of triazido core **6** (50.0 mg, 109 µmol, 1.00 equiv) and mannoside **7** (158 mg, 409 µmol, 3.75 equiv) in a THF/H_2O mixture (6 mL, 1:1) were added sodium ascorbate (19.4 mg, 98.1 µmol, 0.90 equiv) and $CuSO_4{\cdot}5H_2O$ (24.5 mg, 98.1 µmol, 0.90 equiv). The reaction mixture was stirred at 50 °C for 3 h then at room temperature for an additional 16 h period. EtOAc (10 mL) was added and the resulting solution was poured in a separatory funnel containing 35 mL of EtOAc and 30 mL of a saturated aqueous solution of NH_4Cl. Organics were washed with (2 × 25 mL) of saturated NH_4Cl_{aq}, water (2 × 20 mL) and brine (10 mL). The organic phase was then dried over $MgSO_4$ and concentrated under reduced pressure. Column chromatography on silica (DCM/MeOH 98:2 to 94:6) afforded the desired compound **8** (138 mg, 86.0 µmol, 79%) as a viscous oil.

R_f = 0.34 (95:5 DCM/MeOH). **^{1}H NMR (600 MHz, $CDCl_3$)** δ (ppm) 8.27 (s, 3H, CH_{ar}), 7.79 (s, 3H, $CH_{triazole}$), 7.72 (t, *J* = 5.3 Hz, 3H, N*H*), 5.29−5.19 (m, 9H, H_2, H_3, H_4), 4.92 (s_{app}, 3H, H_1), 4.77−4.62 (2×d, *J* = 12.4 Hz, 6H, OCH_2), 4.54 (t, *J* = 6.4 Hz, 6H, $N_{triazole}CH_2$), 4.28 (dd, *J* = 12.4 Hz, *J* = 5.4 Hz, 3H, H_{6b}), 4.11−4.03 (m, 6H, H_5 + H_{6a}), 3.55 (m, 6H, $NHCH_2$), 2.28 (m, 6H, $CH_2CH_2CH_2$), 2.12, 2.10, 2.02, 1.96 (4s, 36H, $COCH_3$).**^{13}C{^{1}H} NMR (150 MHz, $CDCl_3$)** δ (ppm) 170.8, 170.1, 170.0, 169.7 ($COCH_3$), 166.1 (*C*ONH), 143.5 ($C_{triazole}$), 134.9 (C_{arom}), 128.5 (CH_{arom}), 123.9 ($CH_{triazole}$), 96.7 (C_1), 69.3 (C_2), 69.0 (C_3), 68.7 (C_5), 65.9 (C_6), 62.3 (C_4), 60.7 (OCH_2), 48.3 ($CH_2N_{triazole}$), 37.5 ($NHCH_2$), 29.9 ($CH_2CH_2CH_2$), 20.9, 20.8, 20.7, 20.7 ($COCH_3$). **MS** ($^+$TOF-MS) m/z: calculated for $C_{69}H_{90}N_{12}O_{33}$ $[M+H]^+$: 1615.6, found: 1615.6.

Synthesis of trivalent derivative (9): Derivative **9** was synthesized according to **Procedure B** with compound **8** (120.0 mg, 74.3 μmol) previously dissolved in anhydrous MeOH (4 mL). Deprotected hexamer **9** was obtained as a white solid (78.0 mg, 70.6 μmol) in a 95% yield.

^{1}H NMR (300 MHz, D_2O) δ (ppm) 8.03 (s, 3H, CH_{ar}), 8.00 (s, 3H, C$H_{triazole}$), 4.85 (m, 3H, H_1), 4.68−4.49 (m, 12H, OCH_2C$_{triazole}$+ N$_{triazole}$CH_2), 3.80−3.55 (m, 18H, H_2 + H_6 + H_4 + H_3 + H_5), 3.40 (m, 6H, OCNHCH_2), 2.24 (m, 6H, $CH_2CH_2CH_2$).**^{13}C{^{1}H} NMR (75 MHz, $CDCl_3$)** δ (ppm) 168.6 (*C*ONH), 144.2 ($C_{triazole}$), 135.0 (C_{arom}), 129.3 (CH_{arom}), 125.8 ($CH_{triazole}$), 100.0 (C_1), 73.6 (C_5), 71.1 (C_3), 70.6 (C_2), 67.3 (C_4), 61.5 (C_6), 60.7 (OCH_2), 49.1 ($CH_2N_{triazole}$), 38.1 (OCHNCH_2), 29.3 ($CH_2CH_2CH_2$). **HRMS** ($^+$TOF-HRMS) m/z: calculated for $C_{45}H_{66}N_{12}O_{21}$ $[M+H]^+$: 1111.4538, found: 1111.4533 (Δ = −0.52 ppm); $[M+Na]^+$: 1133.4358, found: 1133.4347 (Δ = −0.93 ppm).

Synthesis of peracetylated nonavalent derivative (11): Derivative **11** was synthesized according to **Procedure A** with nonapropargylated core **10** (20.0 mg, 23.2 μmol, 1.00 equiv), mannoside **3** (108.9 mg, 261.0 μmol, 11.25 equiv), sodium ascorbate (12.4 mg, 62.7 μmol, 2.70 equiv), and $CuSO_4 \cdot 5H_2O$ (15.7 mg, 62.7 μmol, 2.70 equiv) in a THF/H_2O mixture (3 mL, 1:1). Column chromatography on silica (DCM/MeOH 98:2 to 94:6) afforded the desired compound **11** (88.0 mg, 19.1 μmol, 83%) as a colorless oil.

R_f = 0.19 (95:5 DCM/MeOH). **^{1}H NMR (300 MHz, $CDCl_3$)** δ (ppm) 8.18 (br s, 3H, CH_{ar}), 7.77 (s, 9H, C$H_{triazole}$), 7.17 (m, 3H, NH), 5.28−5.15 (m, 27H, H_2, H_3, H_4), 4.80 (br s, 9H, H_1), 4.64−4.53 (m, 36H, OCH_2C$_{triazole}$ + N$_{triazole}$CH_2), 4.20−3.98 (m, 27H, OCH_2CH$_2$ + H_{6a}), 3.90−3.85 (m, 27H, H_{6b} + NHC$_q$CH_2O), 3.59 (m, 9H, H_5), 2.09, 2.05, 2.01, 1.96 (4s, 108H, COCH_3).**^{13}C{^{1}H} NMR (75 MHz, $CDCl_3$)** δ (ppm) 170.6, 169.9, 169.6, 169.7 (*C*OCH$_3$), 166.3 (*C*ONH), 144.8 ($C_{triazole}$), 135.5 (C_{arom}), 128.5 (CH_{arom}), 124.0 ($CH_{triazole}$), 97.3 (C_1), 69.0 (C_2), 68.8 (C_3), 68.7 (C_5), 68.4 (NHC$_q$*C*H$_2$O), 66.1 (C_6), 65.5 (C_4), 64.4 (O*C*H$_2$C$_{triazole}$), 62.2 (O*C*H$_2$CH$_2$), 60.5 (C_q), 49.5 (*C*H$_2$N$_{triazole}$), 20.8, 20.7, 20.6, 20.6 (CO*C*H$_3$). **HRMS** ($^+$ESI-MS) m/z: calculated for $C_{192}H_{258}N_{30}O_{102}$ $[M+2H]^{2+}$: 2308.8035, found: 2308.7995 (Δ = −1.73 ppm).

Synthesis of de-*O*-acetylated nonavalent derivative (12): A solution of sodium methoxide (1 M in MeOH, 150 μL) was added to a solution of acetylated precursor **11** (70.0 mg, 15.2 μmol) in MeOH (2.5 mL). The reaction mixture was stirred at room temperature for 24 h. The pH was adjusted to 7 with addition of ion-exchange resin (Amberlite IR 120 H^+). After filtration, the solvent was removed under vacuum with rotary evaporator and the residue was lyophilized to furnish desired de-*O*-acetylated compound **12** (47.0 mg, 15.2 μmol, 99%) as a white solid.

^{1}H NMR (300 MHz, D_2O) δ (ppm) 8.02 (m, 12H, CH_{ar} + C$H_{triazole}$), 4.74 (m, 9H, H_1), 4.62−4.56 (m, 36H, OCH_2C$_{triazole}$ + N$_{triazole}$CH_2), 4.05−4.01 (m, 9H, OCHHCH$_2$N), 3.89−3.78 (m, 36H, OCHHCH$_2$N + H_2 + NHC$_q$CH_2O), 3.71−3.53 (m, 36H, H_6 + H_4 + H_3), 3.02 (m, 9H, H_5). **^{13}C{^{1}H} NMR (75 MHz, D_2O)** δ (ppm)

169.0 (CONH), 144.7 ($C_{triazole}$), 135.8 (C_{arom}), 129.7 (CH$_{arom}$), 126.1 (CH$_{triazole}$), 100.2 (C_1), 73.4 (C_5), 71.1 (C_3), 70.5 (C_2), 67.9 (NHC$_q$$CH_2$O), 67.0 ($C_4$), 66.1 (O$CH_2CH_2$N), 64.2 (O$CH_2C_{triazole}$), 61.4 ($C_q$), 61.3 ($C_6$), 50.7 ($CH_2N_{triazole}$). **HRMS** ($^+$TOF-HRMS) m/z: calculated for $C_{120}H1_{86}N_{30}O_{66}$ $[M+2H]^{2+}$: 1552.6132, found: 1552.6119 (Δ = −0.08 ppm).

Synthesis of nonapropargylated derivative (14): To a solution of phloroglucinol (**13**, 10.0 mg, 79.3 µmol, 1.00 equiv) in anhydrous DMF (3 mL) was added under nitrogen anhydrous K_2CO_3 (previously heated at 250 °C under vaccum, 39.5 mg, 285 µmol, 3.60 equiv). After 10 min of vigorous stirring, tripropargylated synthon**14** (93.0 mg, 285 µmol, 3.60 equiv) was added into the solution under inert atmosphere and the reaction mixture was allowed to stir at 65 °C for 39 h. In the end, the dark-brown heterogeneous reaction was poured in 30 mL of EtOAc and organics were washed with a saturated aqueous solution of NH_4Cl (2 × 30 mL) then water (2 × 20 mL) and brine (10 mL). The organic phase was then dried over $MgSO_4$ and concentrated under reduced pressure. Column chromatography on silica (EtOAc/hexane 40:60 to 50:50) afforded the desired compound **15** (32.0 mg, 33.8 µmol, 43%) as a colorless oil.
R_f = 0.27 (1:1 EtOAc/Hexane). **^{1}H NMR (300 MHz, $CDCl_3$)** δ (ppm) 6.85 (s, 3H, NH), 6.17 (s, 3H, CH_{ar}), 4.36 (s, 6H, OCH_2CONH), 4.16 (m, 18H, OCH_2C≡CH), 3.87 (br s, 18H, HNC$_q$CH_2O), 2.48 (m, 9H, OCH$_2$C≡CH). **^{13}C{^{1}H} NMR (75 MHz, $CDCl_3$)** δ (ppm) 167.3 (CONH), 159.0 (C_{ar}OCH$_2$), 95.8 (CH$_{ar}$), 79.4 (OCH$_2$$C$≡CH), 74.9 (OCH$_2$C≡$C$H), 68.3 (HNC$_q$$CH_2$O), 67.5 (O$CH_2$CONH), 59.2 ($C_q$), 58.6 (O$CH_2$C≡CH). **HRMS** ($^+$TOF-HRMS) m/z: calculated for $C_{51}H_{57}N_3O_{15}$ $[M+H]^+$: 952.3862, found: 952.3843 (Δ = -2.10 ppm); $[M+Na]^+$: 974.3682, found: 974.3662 (Δ = −2.05 ppm).

Synthesis of peracetylated derivative (16): 16 was synthesized according to **Procedure A** with nonapropargylated core **15** (20.0 mg, 21.0 µmol, 1.00 equiv), mannoside **3** (98.6 mg, 236.0 µmol, 11.25 equiv), sodium ascorbate (11.2 mg, 56.7 µmol, 2.70 equiv), and $CuSO_4 \cdot 5H_2O$ (14.2 mg, 56.7 µmol, 2.70 equiv) in a THF/H_2O mixture (3 mL, 1:1). Column chromatography on silica (DCM/MeOH 98:2 to 94:6) afforded the desired compound **16** (86.0 mg, 18.3 µmol, 87%) as a colorless oil.
R_f = 0.24 (93:7 DCM/MeOH). **^{1}H NMR (300 MHz, $CDCl_3$)** δ (ppm) 7.70 (br s, 9H, C$H_{triazole}$), 7.05 (br s, 3H, NH), 6.22 (br s, 3H, CH_{ar}), 5.26−5.17 (m, 27H, H_2, H_3, H_4), 4.79 (br s, 9H, H_1), 4.60 (br s, 36H, OCH_2C$_{triazole}$ + N$_{triazole}$CH_2), 4.18 (br s, 6H, OCH_2CONH), 4.16−3.91 (m, 27H, OCH_2CH$_2$ + H_{6a}), 3.90−3.84 (m, 27H, H_{6b} + NHC$_q$CH_2O), 3.60 (m, 9H, H_5), 2.11, 2.07, 2.02, 1.96 (4s, 108H, COCH_3). **^{13}C{^{1}H} NMR (75 MHz, $CDCl_3$)** δ (ppm) 170.5, 169.8, 169.8, 169.5, (COCH$_3$), 167.5 (CONH), 158.9 (C_{ar}OCH$_2$), 144.6 ($C_{triazole}$), 123.9 (CH$_{triazole}$), 97.3 (C_1), 95.4 (CH$_{ar}$), 69.0 (C_2), 68.8 (C_3), 68.7 (C_5), 68.5 (NHC$_q$$CH_2$O), 67.2 (O$CH_2$CONH), 66.1 ($C_6$), 65.5 ($C_4$), 64.3 (O$CH_2C_{triazole}$), 62.0 (O$CH_2CH_2$), 59.6 ($C_q$), 49.5 ($CH_2N_{triazole}$),

20.7, 20.6, 20.6, 20.6 (COCH_3). **HRMS** (+TOF-HRMS) m/z: calculated for $C_{195}H_{264}N_{30}O_{105}$ $[M+3H]^{3+}$: 1569.5486, found: 1569.5458 (Δ = -1.82 ppm); $[M+2H]^{2+}$: 2353.8193, found: 2353.8088 (Δ = −4.48 ppm).

Synthesis of de-*O*-acetylated nonavalent derivative (17): Derivative **17** was synthesized according to **Procedure B** with compound **16** (45.0 mg, 9.56 µmol) previously dissolved in anhydrous MeOH (3 mL). Deprotected nonamer **17** was obtained as a white solid (27.0 mg, 8.45 µmol) in a 90% yield.

^{1}H NMR (300 MHz, D_2O) δ (ppm) 7.97 (m, 9H, $CH_{triazole}$), 6.15 (s, 3H, CH_{ar}), 4.77 (s, 9H, H_1), 4.61−4.46 (m, 42H, $OCH_2C_{triazole}$ + $N_{triazole}CH_2$ + OCH_2CONH)), 4.06−4.01 (m, 9H, OCH*H*CH_2N), 3.90−3.56 (m, 72H, C*H*HCH_2N + H_2 + NHC_qCH_2O + H_6 + H_4 + H_3), 3.06 (m, 9H, H_5). **^{13}C{^{1}H} NMR (75 MHz, D_2O)** δ (ppm) 170.5 (*C*ONH), 159.7 ($C_{ar}OCH_2$), 144.7 ($C_{triazole}$), 126.0 ($CH_{triazole}$), 100.2 (C_1), 96.1 (CH_{ar}), 73.4 (C_5), 71.1 (C_3), 70.6 (C_2), 68.4 (NHC_qCH_2O), 67.5 (OCH_2CONH), 67.0 (C_4), 66.1 (OCH_2CH_2N), 64.2 (O$CH_2C_{triazole}$), 61.3 (C_6), 60.5 (C_q), 50.7 ($CH_2N_{triazole}$). **HRMS** (+TOF-HRMS) m/z: calculated for $C_{123}H_{192}N_{30}O_{69}$ $[M+3H]^{3+}$: 1065.4219, found: 1065.4221 (Δ = 0.23 ppm).

Synthesis of bromoacylated dendron (18): 18 was synthesized according to **Procedure A** with tripropargylated synthon **14** (140.0 mg, 393.0 µmol, 1.00 equiv), mannoside **3** (616 mg, 1.48 mmol, 3.75 equiv), sodium ascorbate (70.0 mg, 354 µmol, 0.90 equiv), and $CuSO_4{\cdot}5H_2O$ (88.4 mg, 354 µmol, 0.90 equiv) in a THF/H_2O mixture (6 mL, 1:1). Column chromatography on silica (DCM/MeOH 99:1 to 96:4) afforded the desired compound **18** (594 mg, 369.4 µmol, 94%) as a white solid.

R_f = 0.47 (94:6 DCM/MeOH). **m.p.** = 68-72°C (*not corrected*). **^{1}H NMR (300 MHz, $CDCl_3$)** δ (ppm) 7.68 (br s, 3H, $CH_{triazole}$), 6.89 (br s, 1H, N*H*), 5.24−5.18 (m, 9H, H_2, H_3, H_4), 4.80 (d, *J* = 1.3 Hz, 1H, H_1), 4.61−4.58 (br s, 12H, $OCH_2C_{triazole}$ + $N_{triazole}CH_2$), 4.17−4.00 (m, 11H, OCH_2CH_2 + H_{6a} + BrCH_2CONH), 3.94−3.78 (m, 9H, H_{6b} + NHC_qCH_2O), 3.60 (m, 3H, H_5), 2.12, 2.08, 2.03, 1.98 (4s, 36H, COCH_3). **^{13}C{^{1}H} NMR (75 MHz, $CDCl_3$)** δ (ppm) 170.5, 169.9, 169.9, 169.5, (*C*OCH_3), 165.6 (*C*ONH), 145.0 ($C_{triazole}$), 123.7 ($CH_{triazole}$), 97.4 (C_1), 69.1 (C_2), 68.9 (C_3), 68.8 (C_5), 68.4 (NHC_qCH_2O), 66.2 (C_6), 65.6 (C_4), 64.6 (O$CH_2C_{triazole}$), 62.1 (OCH_2CH_2), 60.2 (C_q), 49.6 ($CH_2N_{triazole}$), 29.7 (CH_2Br), 20.8, 20.7, 20.6, 20.6 (COCH_3).IR (cm^{-1}) 2956, 2937, 2361, 2337, 1751, 1734, 1540, 1370, 1218, 1045, 759. **HRMS** (+TOF-HRMS) m/z: calculated for $C_{63}H_{87}BrN_{10}O_{34}$ $[M+2H]^{2+}$: 804.2358, found: 804.2356 (Δ = -0.18 ppm); $[M+H]^+$: 1607.4642, found: 1607.4620 (Δ = -1.36 ppm); $[M+Na]^+$: 1629.4462, found: 1629.4448 (Δ = −0.84 ppm).

Synthesis of azidoacylated dendron (19): To a stirring solution of brominated trivalent dendron**18** (121.0 mg, 75.2 µmol, 1.00 equiv) in dry DMF (1.5 mL) was added under a nitrogen atmosphere sodium azide (7.3 mg, 112 µmol, 1.50 equiv). After stirring overnight at room temperature, the solvent was removed under vaccum. EtOAc (20 mL) was added and the resulting solution was poured in a separatory funnel

containing 20 mL of EtOAc and 30 mL of a saturated aqueous solution of NH_4Cl. Organics were washed with (2 × 30 mL) of saturated NH_4Cl_{aq}, water (2 × 30 mL) and brine (20 mL). The organic phase was then dried over $MgSO_4$ and concentrated under reduced pressure to furnish the desired compound **19** (110 mg, 69.9 µmol, 93%) as a white solid.

R_f = 0.47 (94:6 DCM/MeOH). **m.p.** = 62-65°C (*not corrected*). **^{1}H NMR (300 MHz, $CDCl_3$)** δ (ppm) 7.68 (br s, 3H, $CH_{triazole}$), 6.69 (br s, 1H, N*H*), 5.27−5.18 (m, 9H, H_2, H_3, H_4), 4.80 (d, J = 1.3 Hz, 1H, H_1), 4.61−4.58 (br s, 12H, $OCH_2C_{triazole}$ + $N_{triazole}CH_2$), 4.23−4.00 (m, 11H, OCH_2CH_2 + H_{6a} + N_3CH_2CONH), 3.90−3.81 (m, 9H, H_{6b} + NHC_qCH_2O), 3.60 (m, 3H, H_5), 2.12, 2.08, 2.03, 1.98 (4s, 36H, $COCH_3$).**^{13}C{^{1}H} NMR (75 MHz, $CDCl_3$)** δ (ppm) 170.4, 169.9, 169.8, 169.5, ($COCH_3$), 166.7 (*C*ONH), 144.9 ($C_{triazole}$), 123.7 ($CH_{triazole}$), 97.4 (C_1), 69.0 (C_2), 68.8 (C_3), 68.8 (C_5), 68.4 (NHC_qCH_2O), 66.1 (C_6), 65.6 (C_4), 64.5 ($OCH_2C_{triazole}$), 62.1 (OCH_2CH_2), 59.9 (C_q), 52.5 (CH_2N_3), 49.5 ($CH_2N_{triazole}$), 20.7, 20.7, 20.6, 20.6 ($COCH_3$).IR (cm^{-1}) 2934, 2361, 2338, 2107 (N_3), 1751, 1734, 1540, 1373, 1218, 1045, 761. **HRMS** ($^+$TOF-HRMS) m/z: calculated for $C_{63}H_{87}N_{13}O_{34}$ $[M+H]^+$: 1570.5551, found: 1570.5543(Δ = -0.51 ppm); $[M+Na]^+$: 1592.5371, found: 1592.5366(Δ = −0.31 ppm).

Synthesis of peracetylated nonavalent derivative (20): Derivative **20** was synthesized according to **Procedure A** with tripropargylated core **2** (3.9 mg, 12.1 µmol, 1.00 equiv), trimannosylated dendron **19** (75.0 mg, 47.8 µmol, 3.90 equiv), sodium ascorbate (6.5 mg, 33 µmol, 2.70 equiv), and $CuSO_4·5H_2O$ (8.2 mg, 33 µmol, 0.90 equiv) in a THF/H_2O mixture (4 mL, 1:1). Column chromatography on silica (DCM/MeOH 98:2 to 90:10) afforded the desired compound **20** (52.0 mg, 10.2 µmol, 84%) as a colorless oil.

R_f = 0.25 (92:8 DCM/MeOH). **^{1}H NMR (300 MHz, $CDCl_3$)** δ (ppm) 8.37 (m, 3H, CH_{ar}), 8.00 (br s, 3H, N*H*), 7.78 (s, 3H, $CH_{int\text{-}triazole}$), 7.69 (s, 9H, $CH_{ext\text{-}triazole}$), 6.94 (br s, 3H, NH_{int}), 5.24−5.17 (m, 27H, H_2, H_3, H_4), 4.98 (br s, 6H, $N_{triazole}CH_2$CONH), 4.80 (s_{app}, 9H, H_1), 4.64−4.52 (m, 42H, $OCH_2C_{triazole}$ + $N_{triazole}CH_2$ + $NHCH_2C_{triazole}$), 4.19−3.87 (m, 54H, OCH_2 + H_6 + NHC_qCH_2O), 3.63−3.62 (m, 9H, H_5), 2.11, 2.08, 2.01, 1.96 (4s, 108H, $COCH_3$). **^{13}C{^{1}H} NMR (75 MHz, $CDCl_3$)** δ (ppm) 170.5, 170.0, 169.9, 169.5 ($COCH_3$), 165.8 (*C*ONH), 165.1 (*C*ONH), 144.8 ($C_{ext\text{-}triazole}$), 144.7 ($C_{int\text{-}triazole}$), 134.7 (C_{arom}), 128.7 (CH_{arom}), 124.1 ($CH_{int\text{-}triazole}$), 123.4 ($CH_{ext\text{-}triazole}$), 97.4 (C_1), 69.0 (C_2), 68.9 (C_3), 68.7 (C_5), 68.3 (NHC_qCH_2O), 66.1 (C_6), 65.5 (C_4), 64.5 ($OCH_2C_{triazole}$), 62.1 (OCH_2), 60.3 (C_q), 52.4 ($N_{triazole}CH_2$CONH), 49.5 ($CH_2N_{triazole}$), 35.5 ($HNCH_2C_{triazole}$), 20.8, 20.7, 20.6, 20.6 ($COCH_3$). **HRMS** ($^+$TOF-HRMS) m/z: calculated for $C_{207}H_{276}N_{42}O_{105}$ $[M+2H]^{2+}$: 2515.8847, found: 2515.8845 (Δ = -0.08 ppm); $[M+3H]^{3+}$: 1677.5922, found: 1677.5936 (Δ = 0.79 ppm).

Synthesis of de-*O*-acetylated nonavalent derivative (21): Derivative **21** was synthesized according to **Procedure B** with **20** (40.0 mg, 7.95 µmol) previously dissolved in anhydrous MeOH (3 mL). After

filtration, the solvent was removed under vacuum with rotary evaporator and the residue was lyophilized to furnish desired de-*O*-acetylated nonamer **21** as a white solid (28.0 mg, 7.95 µmol) in a 99% yield.

^{1}H NMR (600 MHz, D_2O) δ (ppm) 8.27 (m, 3H, CH_{ar}), 7.96 (m, 12H, $CH_{triazole}$), 5.15 (br s, 6H, $N_{triazole}CH_2CONH$), 4.72 (s, 9H, H_1), 4.66 (s, 6H $OCNHCH_2C_{triazole}$), 4.57−4.51 (m, 36H, $OCH_2C_{triazole}$ + $N_{triazole}CH_2$), 4.06−4.01 (m, 9H, $OCHHCH_2N$), 3.83−3.80 (m, 18H, $OCHHCH_2N$ + H_2), 3.69−3.54 (m, 54H, NHC_qCH_2O + H_6 + H_4 + H_3), 3.01 (m, 9H, H_5). **^{13}C{^{1}H} NMR (150 MHz, D_2O)** δ (ppm) 168.4 (*C*ONH), 167.6 (*C*ONH), 145.2 ($C_{ext\text{-}triazole}$), 144.5 ($C_{int\text{-}triazole}$), 135.0 (C_{arom}), 129.8 (CH_{arom}), 126.1 ($CH_{int\text{-}triazole}$), 126.0 ($CH_{ext\text{-}triazole}$), 100.2 (C_1), 73.4 (C_5), 71.1 (C_3), 70.6 (C_2), 67.8 (NHC_qCH_2O), 67.0 ($OCH_2CH_2N_{triazole}$), 66.1 (C_4), 64.1 ($OCH_2C_{triazole}$), 61.3 (C_6), 60.9 (C_q), 52.4 ($N_{triazole}CH_2CONH$), 50.7 ($CH_2N_{triazole}$), 35.7 ($OCHNCH_2C_{triazole}$). **HRMS** ($^{+}$ESI-HRMS) m/z: calculated for $C_{135}H_{204}N_{42}O_{69}$ $[M+3H]^{3+}$: 1173.4655, found: 1173.4671 (Δ = 1.44 ppm); $[M+3Na]^{3+}$: 1195.4474, found: 1195.4490 (Δ = 1.31 ppm).

Synthesis of peracetylated 27-mer derivative (22): Derivative **22** was synthesized according to **Procedure A** with nonapropargylated core **10** (4.6 mg, 5.38 µmol, 1.00 equiv), trimannosylated dendron **19** (95.0 mg, 60.5 µmol, 11.25 equiv), sodium ascorbate (2.9 mg, 15 µmol, 2.70 equiv), and $CuSO_4{\cdot}5H_2O$ (3.6 mg, 15 µmol, 0.90 equiv) in a THF/H_2O mixture (3 mL, 1:1). Column chromatography on silica (DCM/MeOH 98:2 to 90:10) afforded the desired compound **22** (50.0 mg, 3.33 µmol, 63%) as a yellowish oil.

R_f = 0.72 (90:10 DCM/MeOH). **^{1}H NMR (600 MHz, $CDCl_3$)** δ (ppm) 8.27 (m, 3H, CH_{ar}), 7.79 (s, 9H, $CH_{int\text{-}triazole}$), 7.75 (s, 27H, $CH_{ext\text{-}triazole}$), 7.34−7.31 (m, 12H, N*H*), 5.23−5.18 (m, 81H, H_2, H_3, H_4), 5.05 (br s, 18H, $N_{triazole}CH_2CONH$), 4.81 (s_{app}, 27H, H_1), 4.62−4.53 (m, 126H, $OCH_2C_{triazole}$ + $N_{triazole}CH_2$), 4.20−3.64 (m, 207H, OCH_2 + H_6 + NHC_qCH_2O + H_5), 2.11, 2.08, 2.01, 1.96 (4s, 324H, $COCH_3$). **^{13}C{^{1}H} NMR (150 MHz, $CDCl_3$)** δ (ppm) 170.6, 170.5, 170.0, 169.9, 169.9, 169.7, 169.6 ($COCH_3$), 168.4 (*C*ONH), 165.4 (*C*ONH), 144.9 + 144.8 ($C_{ext\text{-}triazole}$), 144.5 ($C_{int\text{-}triazole}$), 135.6 (C_{arom}), 128.6 (CH_{arom}), 124.9 ($CH_{int\text{-}triazole}$), 124.0 ($CH_{ext\text{-}triazole}$), 97.5 (C_1), 69.1 (C_2), 69.0 (C_3), 68.7 (C_5), 68.4 (NHC_qCH_2O), 66.2 (C_6), 65.6 (C_4), 64.5 ($OCH_2C_{triazole}$), 62.1 (OCH_2), 60.4 (C_q), 52.4 ($N_{triazole}CH_2CONH$), 49.5 ($CH_2N_{triazole}$), 20.8, 20.8, 20.7, 20.7 ($COCH_3$). **MS** ($^{+}$TOF-MS) m/z: calculated for $C_{615}H_{834}N_{120}O_{318}$ $[M+H]^{+}$: 14995.8, found: 14995.9.

Synthesis of de-*O*-acetylated 27-mer derivative (23): Derivative **23** was synthesized according to **Procedure B** with **22** (30.0 mg, 2.00 µmol) previously dissolved in anhydrous MeOH (3 mL). After filtration, the solvent was removed under vacuum with rotary evaporator and the residue was lyophilized to yield the fully deprotected 27-mer **23** as a white solid (17.0 mg, 1.63 µmol) in a 82% yield.

^{1}H NMR (600 MHz, D_2O) δ (ppm) 8.06 (m, 3H, CH_{ar}), 7.97 (s, 27H, $CH_{ext\text{-}triazole}$), 7.96 (s, 9H, $CH_{int\text{-}triazole}$), 5.14 (br s, 18H, $N_{triazole}CH_2CONH$), 4.75 (s, 27H, H_1), 4.59−4.51 (m, 126H, $OCH_2C_{triazole}$ + $N_{triazole}CH_2$), 4.05−4.03 (m, 27H, $OCHHCH_2N$), 3.83−3.80 (m, 72H, $OCHHCH_2N$ + H_2 + $NHC_qCH_2O_{int}$), 3.71−3.57 (m,

162H, NHC$_q$CH_2O$_{ext}$ + H_6 + H_4 + H_3), 3.01 (m, 27H, H_5). **^{13}C{^{1}H} NMR (150 MHz, D_2O)** δ (ppm) 168.8 (CONH$_{int}$), 167.5 (CONH$_{ext}$), 144.7 ($C_{ext\text{-}triazole}$), 144.6 ($C_{int\text{-}triazole}$), 135.7 (C_{arom}), 129.7 (CH$_{arom}$), 127.0 (CH$_{int\text{-}triazole}$), 126.1 (CH$_{ext\text{-}triazole}$), 100.2 (C_1), 73.5 (C_5), 71.1 (C_3), 70.6 (C_2), 68.2 (NHC$_q$$CH_2$O), 68.0 (NHC$_q$$CH_2$O), 67.0 (O$CH_2CH_2N_{triazole}$), 66.1 ($C_4$), 64.2 (O$CH_2C_{triazole}$), 61.3 ($C_6$), 60.9 ($C_q$), 52.9 (N$_{triazole}CH_2$CONH), 50.7 ($CH_2N_{triazole}$), 35.7 (OCHN$CH_2C_{triazole}$). **HRMS** ($^+$TOF-HRMS) m/z: calculated for $C_{399}H_{204}N_{120}O_{210}$ $[M+7H]^{7+}$: 1494.6002, found: 1494.5951 (Δ = −3.43 ppm).

NMR, IR, and mass spectra

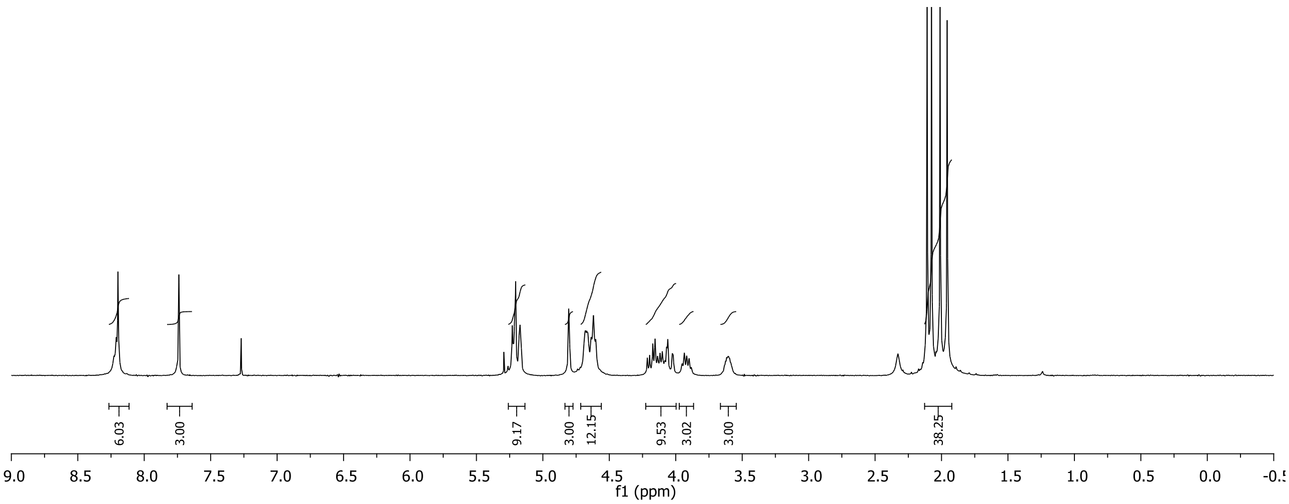

Figure S1: ^{1}H NMR spectrum of compound **4** ($CDCl_3$, 300 MHz).

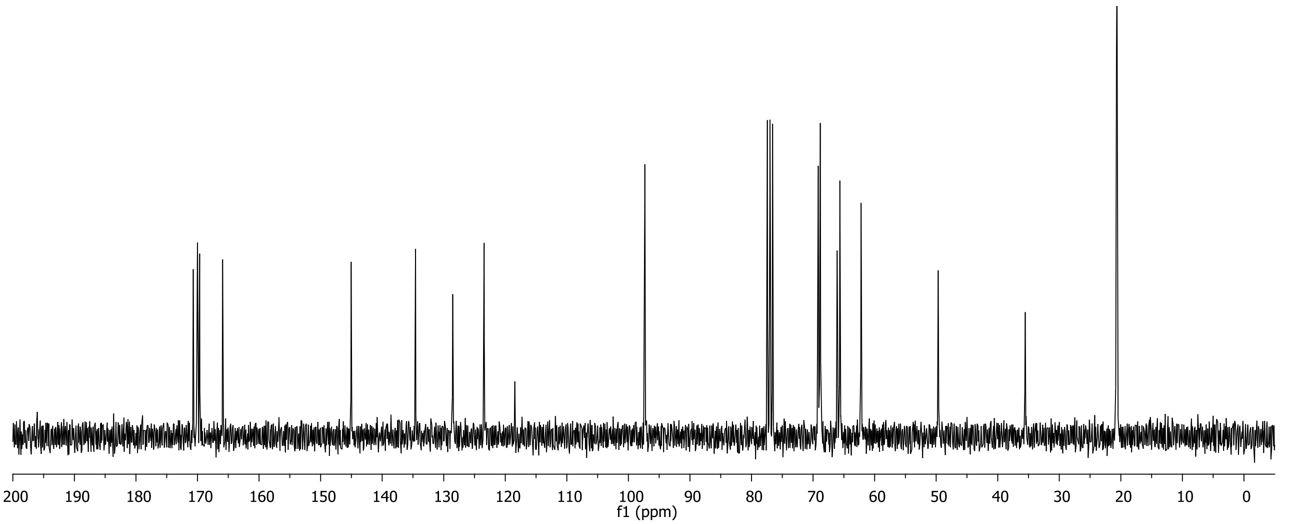

Figure S2: ^{13}C NMR spectrum of compound **4** ($CDCl_3$, 75 MHz).

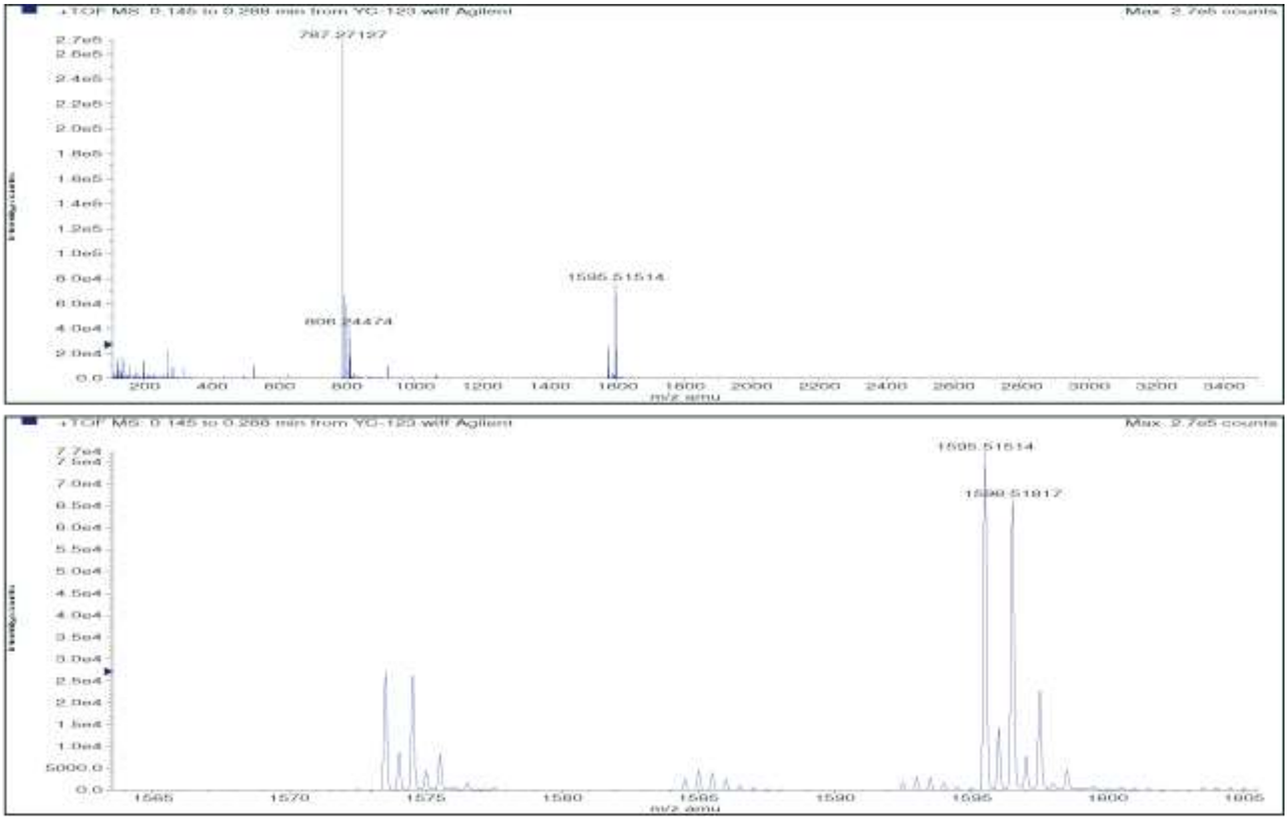

Formula	Compound name	Mass	Peak RT (min)	Peak area	Description
C66H84N12O33	--	1572.52637	0.21	2.87273 E6	--

Species	Abundance (counts)	Ion Mass	Measured Mass	Error (mDa)	Error (ppm)	Ret. Time Error (min)
[M+H]+	27482.99	1573.53365	1573.53226	-1.39237	-0.88	--
[M+Na]+	77859.75	1595.51559	1595.51514	-0.45717	-0.29	--

Figure S3: HRMS analysis ($^{+}$TOF) for compound **4**.

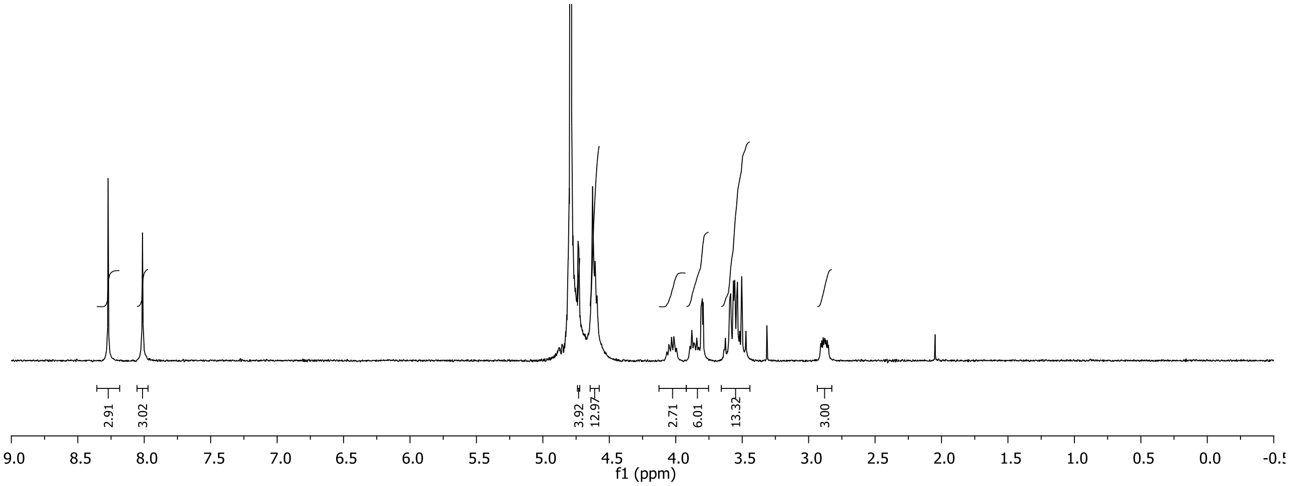

Figure S4: ^{1}H NMR spectrum of compound **5** (D_2O, 300 MHz).

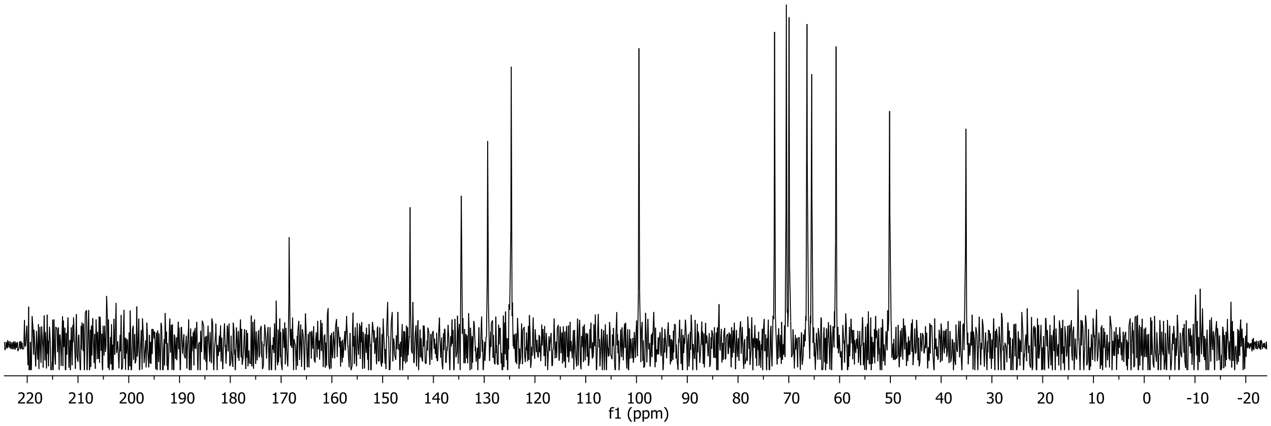

Figure S5: ^{13}C NMR spectrum of compound **5** (D_2O, 75 MHz).

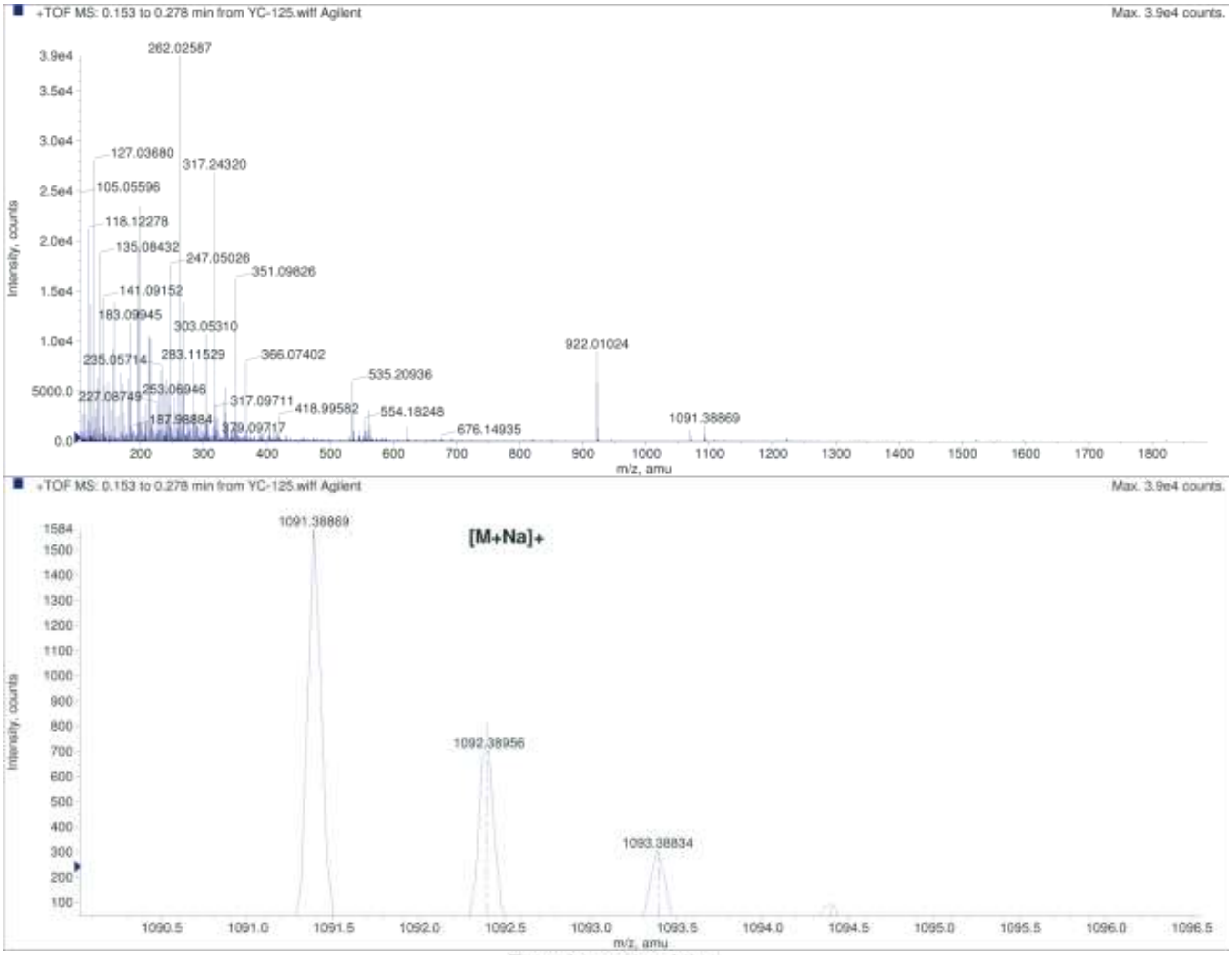

Elemental composition calculator

Target m/z: +1091.3887 amu
Tolerance: +5.0000 ppm
Result type: Elemental
Max num of results: 200
Min DBE: -0.5000 Max DBE: +200.0000
Electron state: OddAndEven
Num of charges: 1
Add water: N/A
Add proton: N/A
File Name: YC-125.wiff

	Elements	Min Number	Max Number
1	C	0	50
2	H	0	80
3	N	0	12
4	O	0	21
5	Na	0	1

	Formula	Calculated m/z (amu)	mDa Error	PPM Error	DBE
1	C42 H60 N12 O21 Na	1091.38881	-0.12905	-0.11824	18.5
2	C44 H59 N12 O21	1091.39122	-2.53431	-2.32210	21.5
3	C47 H60 N10 O19 Na	1091.39284	-4.15176	-3.80410	22.5
4	C50 H59 N8 O20	1091.38401	4.67635	4.28477	25.5

Figure S6: HRMS analysis ($^{+}$TOF) for compound **5**.

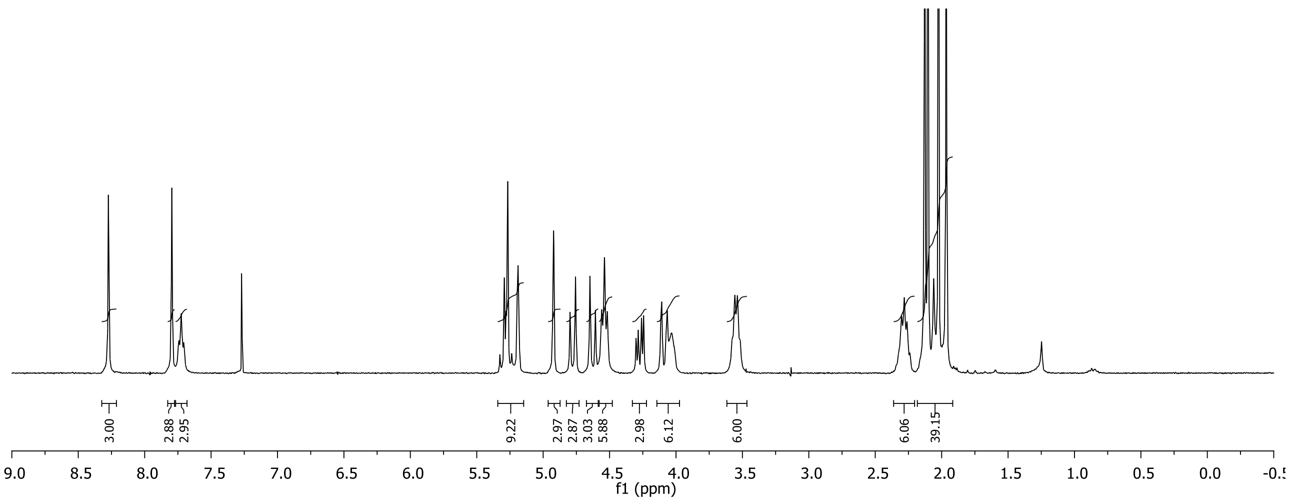

Figure S7: ^{1}H NMR spectrum of compound **8** ($CDCl_3$, 600 MHz).

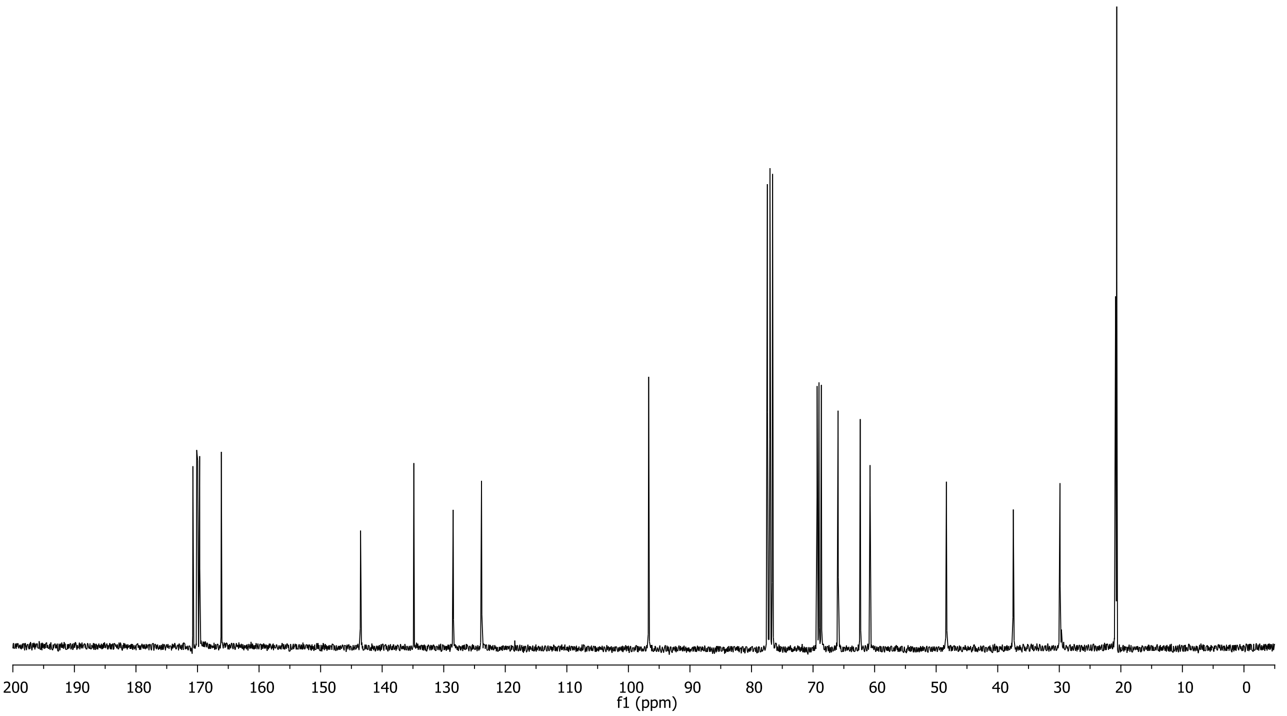

Figure S8: ^{13}C NMR spectrum of compound **8** ($CDCl_3$, 150 MHz).

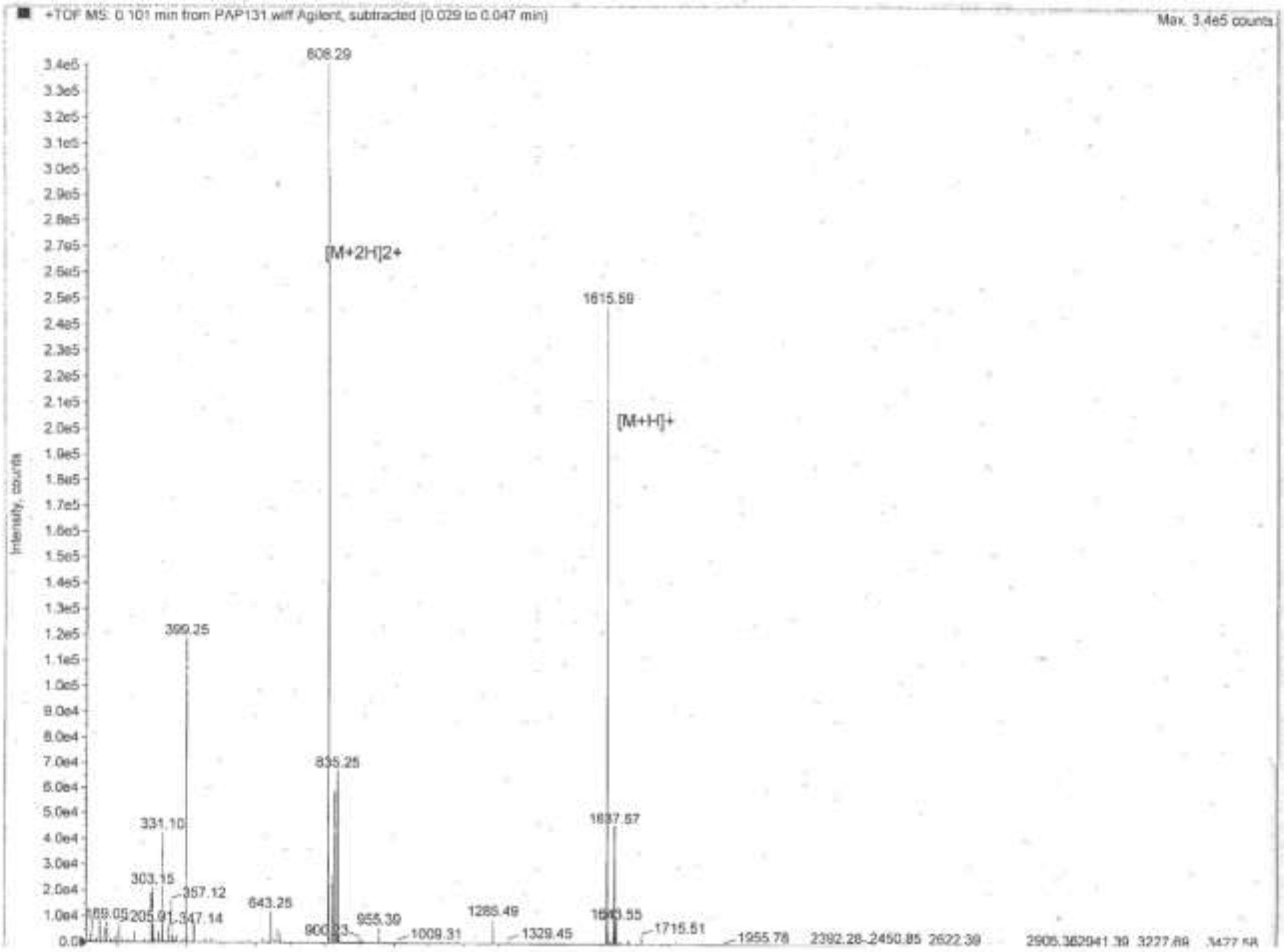

Figure S9: MS analysis ($^{+}$TOF) for compound **8**.

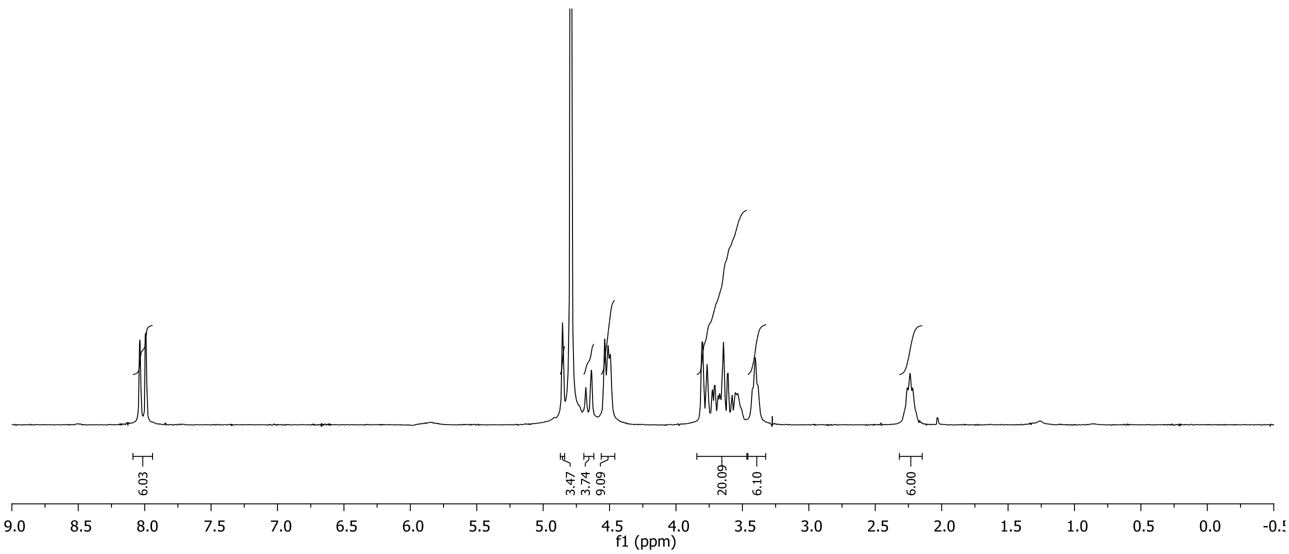

Figure S10: ^{1}H NMR spectrum of compound **9** (D_2O, 300 MHz).

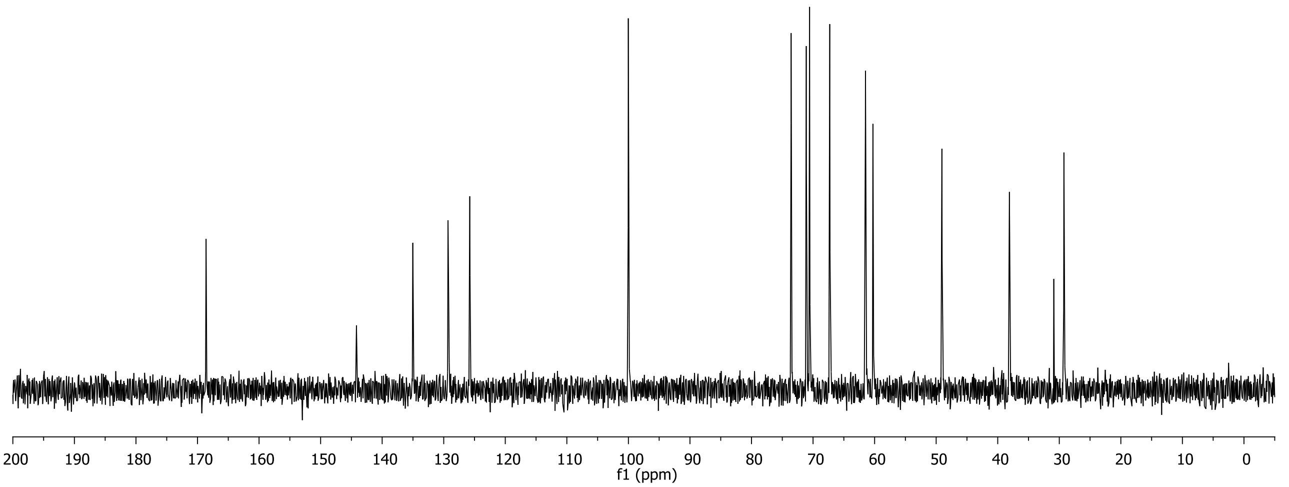

Figure S11: ^{13}C NMR spectrum of compound **9** (D_2O, 75 MHz).

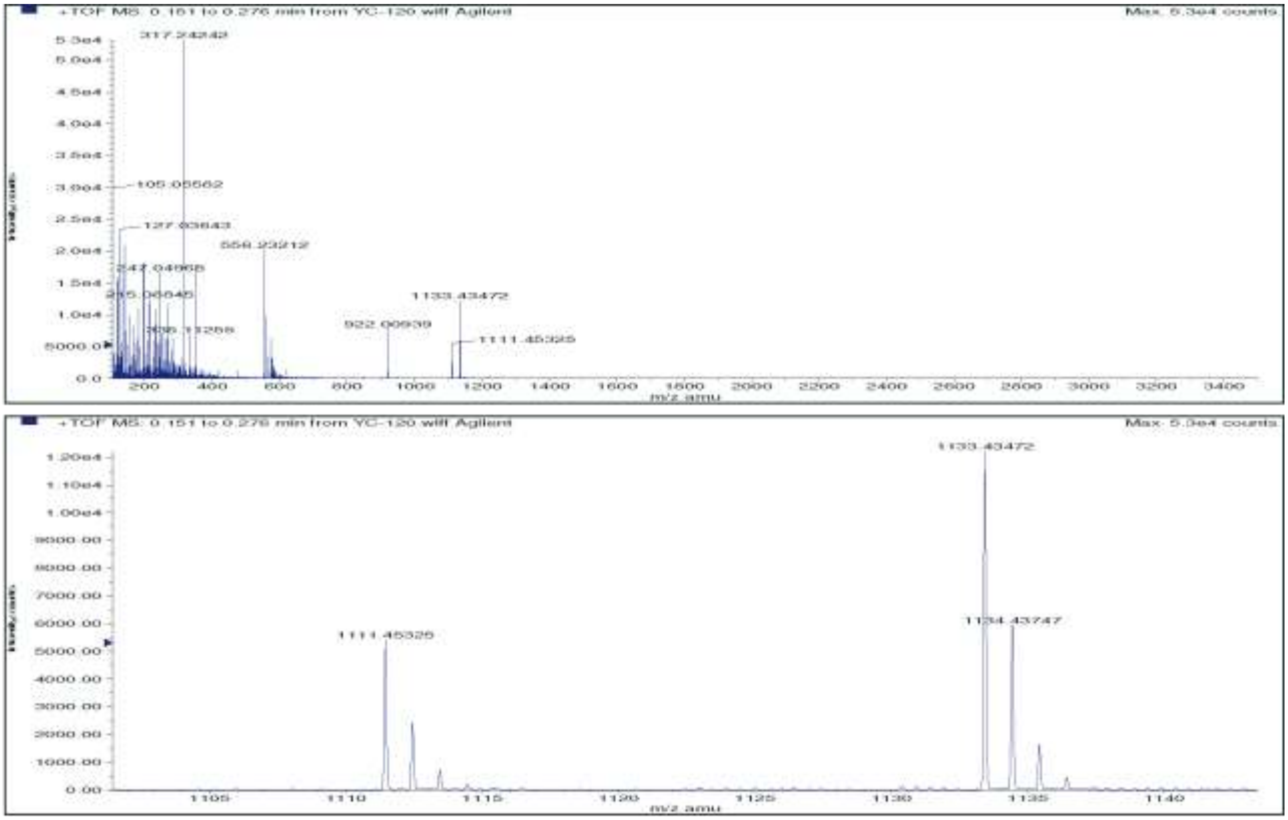

Formula	Compound name	Mass	Peak RT (min)	Peak area	Description
C45H66N12O21	--	1110.44655	0.18	3.89733 E5	--

Species	Abundance (counts)	Ion Mass	Measured Mass	Error (mDa)	Error (ppm)	Ret. Time Error (min)
[M+H]+	5408.33	1111.45382	1111.45325	-0.57258	-0.52	--
[M+Na]+	12232.50	1133.43577	1133.43472	-1.05035	-0.93	--

Figure S12: HRMS analysis ($^{+}$TOF) for compound **9**.

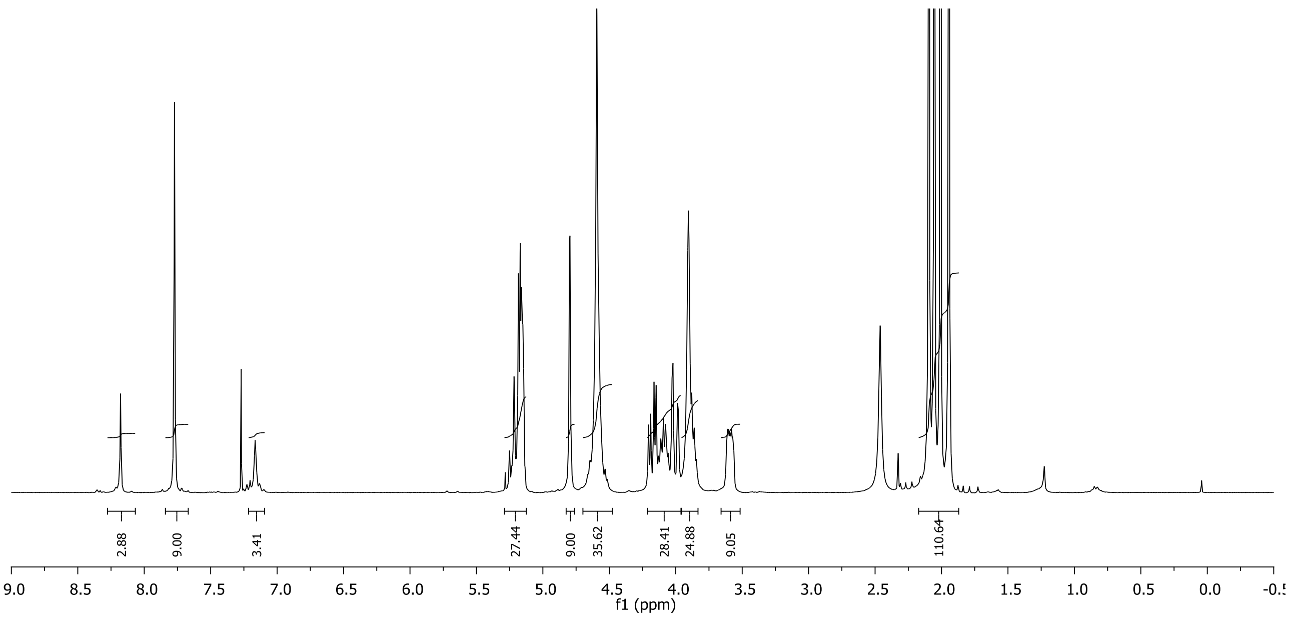

Figure S13: ^{1}H NMR spectrum of compound **11** ($CDCl_3$, 300 MHz).

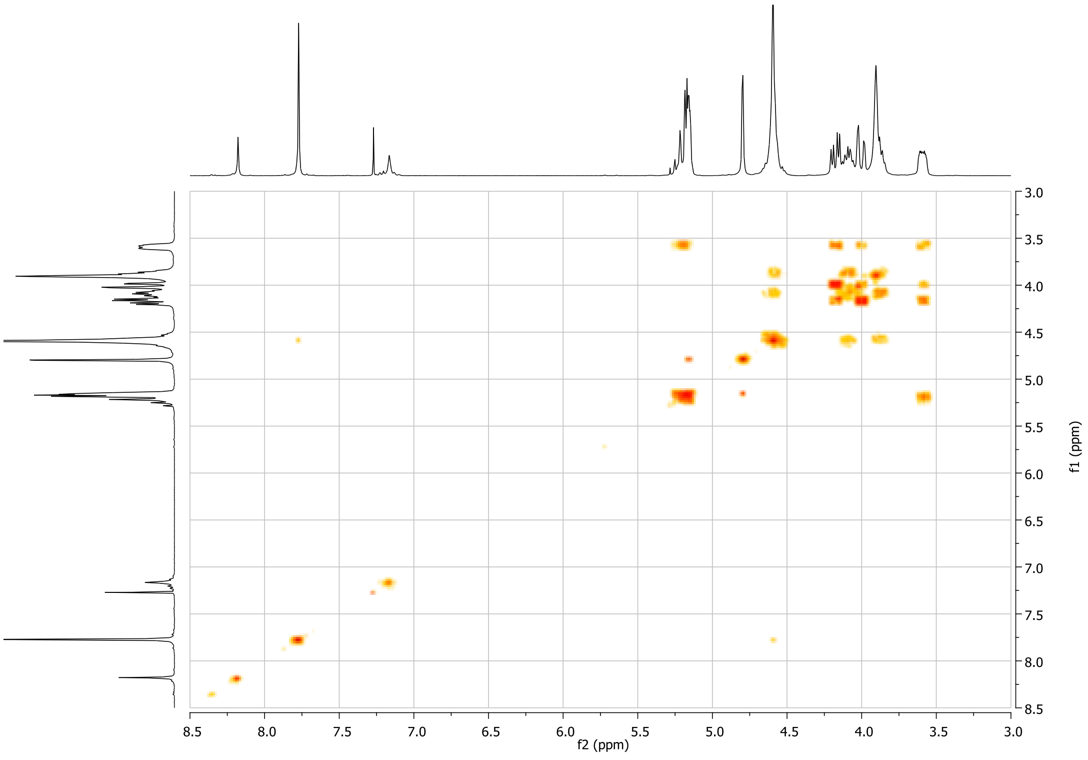

Figure S14: COSY spectrum of compound **11** ($CDCl_3$).

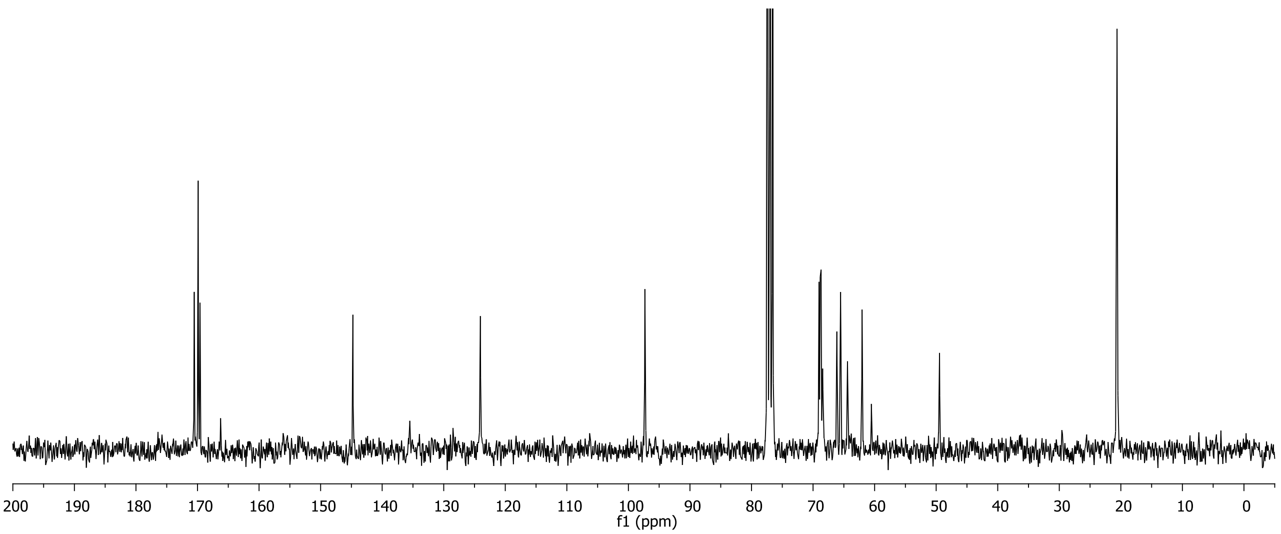

Figure S15: ^{13}C NMR spectrum of compound **11** ($CDCl_3$, 75 MHz).

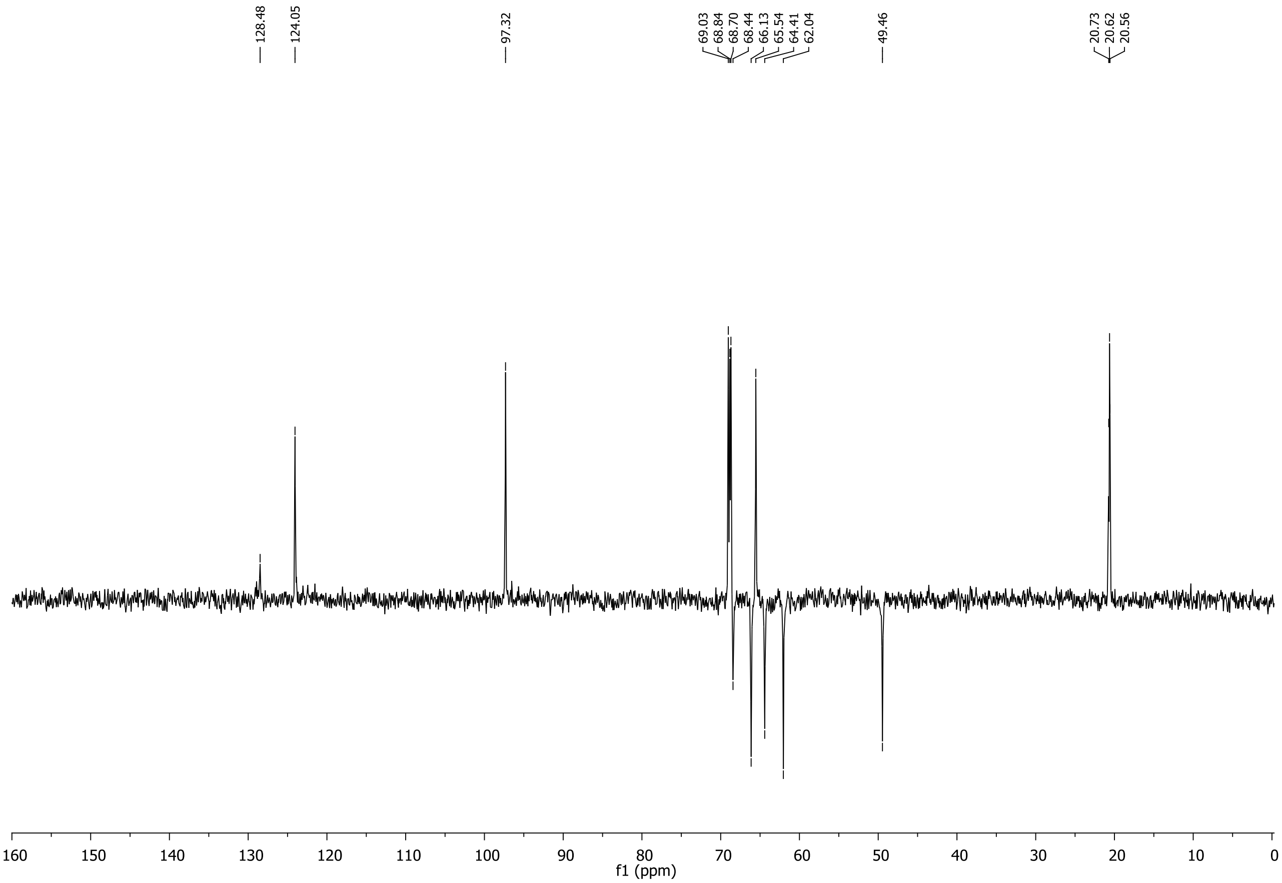

Figure S16: DEPT 135 spectrum for compound **11**.

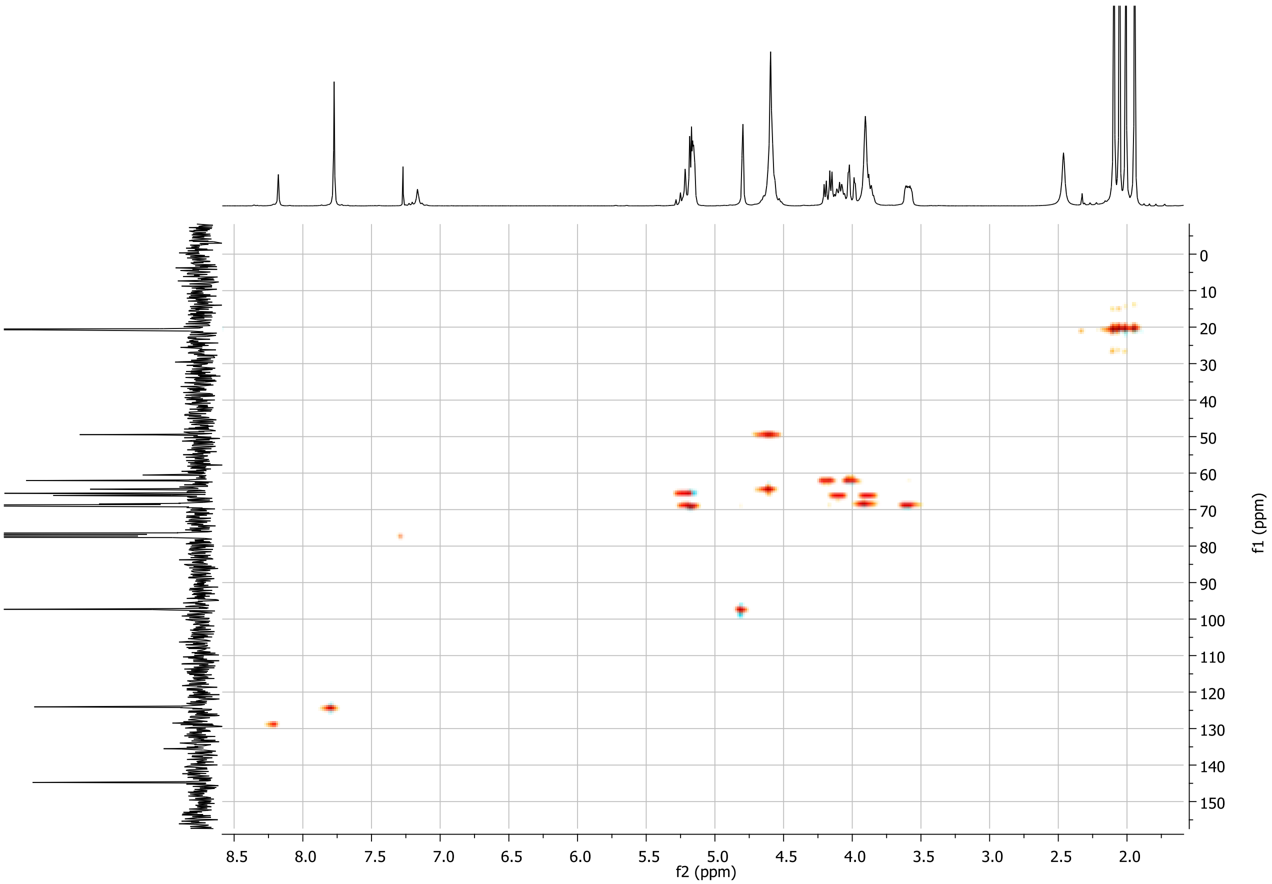

Figure S17: HSQC spectrum for compound **11**.

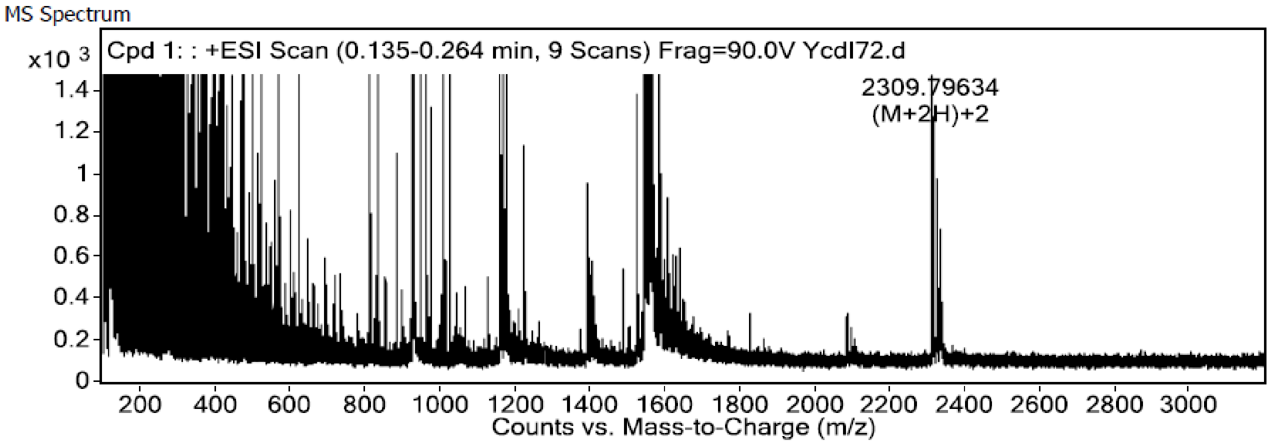

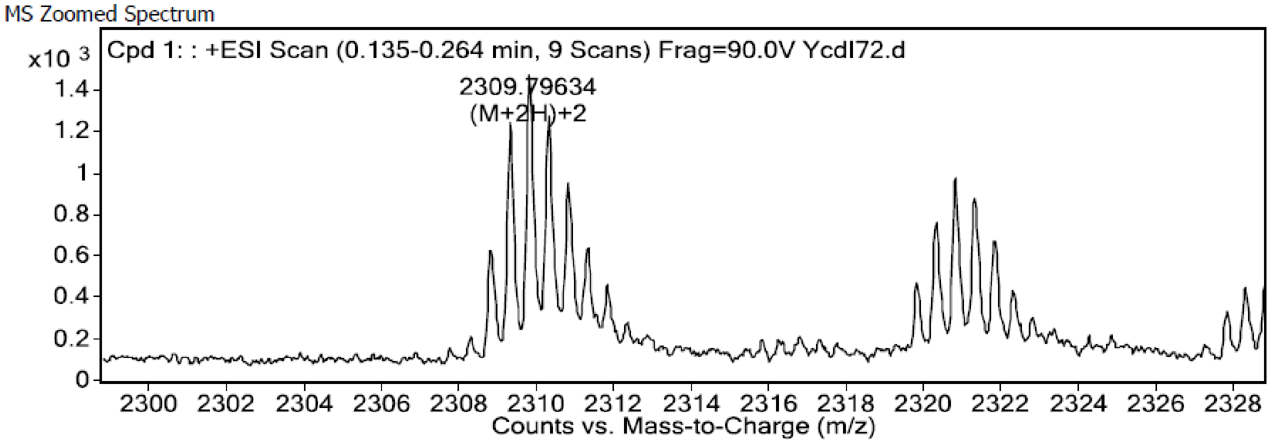

MS Spectrum Peak List

Ion	Ion Formula	Abund	*Expe. m/z*	*Calc. m/z*	Diff(ppm)
		717775.9	134.11783		
(M+2H)+2	C192H260N30O102	574.8	2308.79947	2308.80346	-1.73

Figure S18: HRMS spectrum ($^{+}$ESI) for compound **11**.

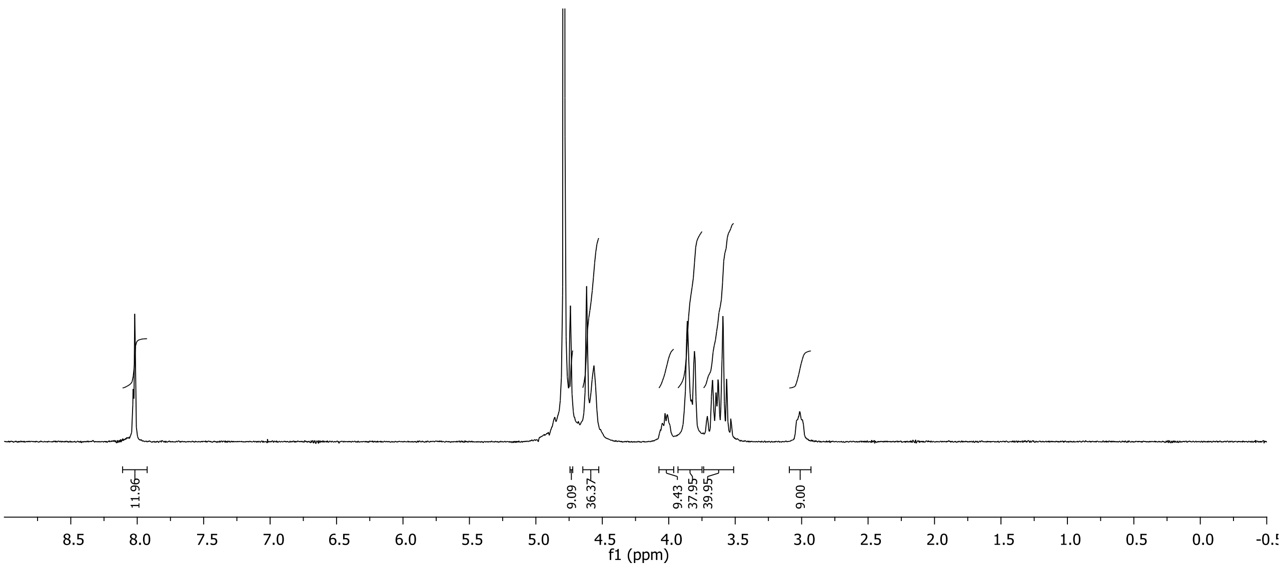

Figure S19: ^{1}H NMR spectrum of compound **12** (D_2O, 300 MHz).

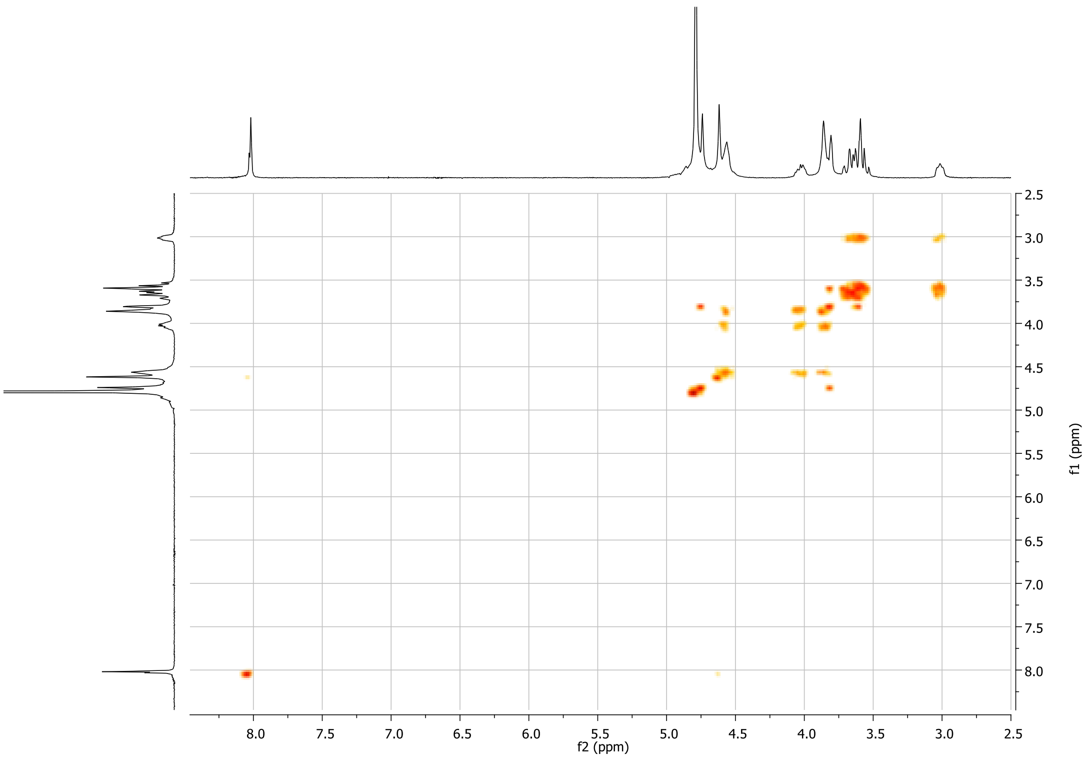

Figure S20: COSY spectrum for compound **12**.

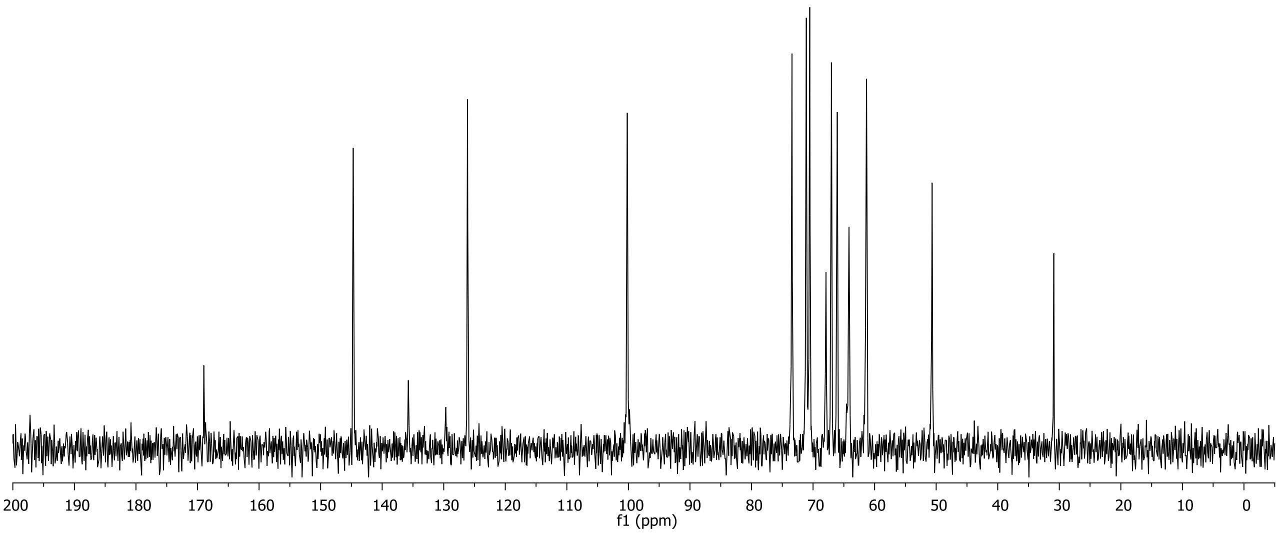

Figure S21: ^{13}C NMR spectrum of compound **12** (D_2O, 75 MHz).

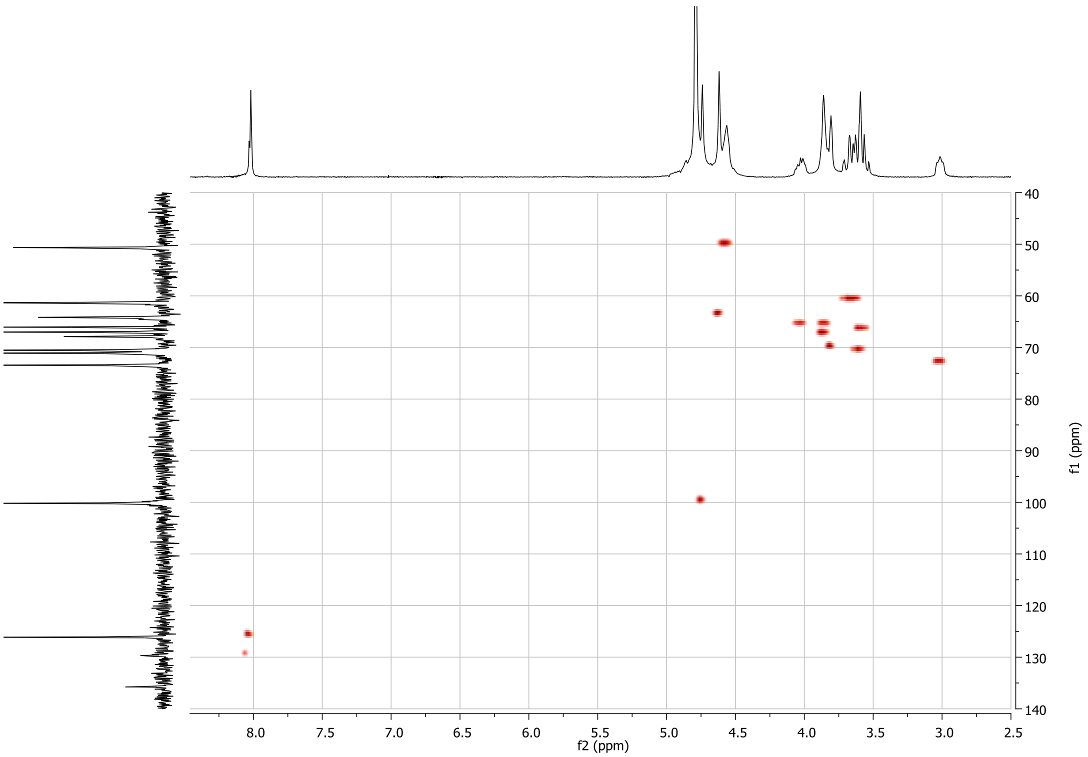

Figure S22: HSQC spectrum for compound **12**.

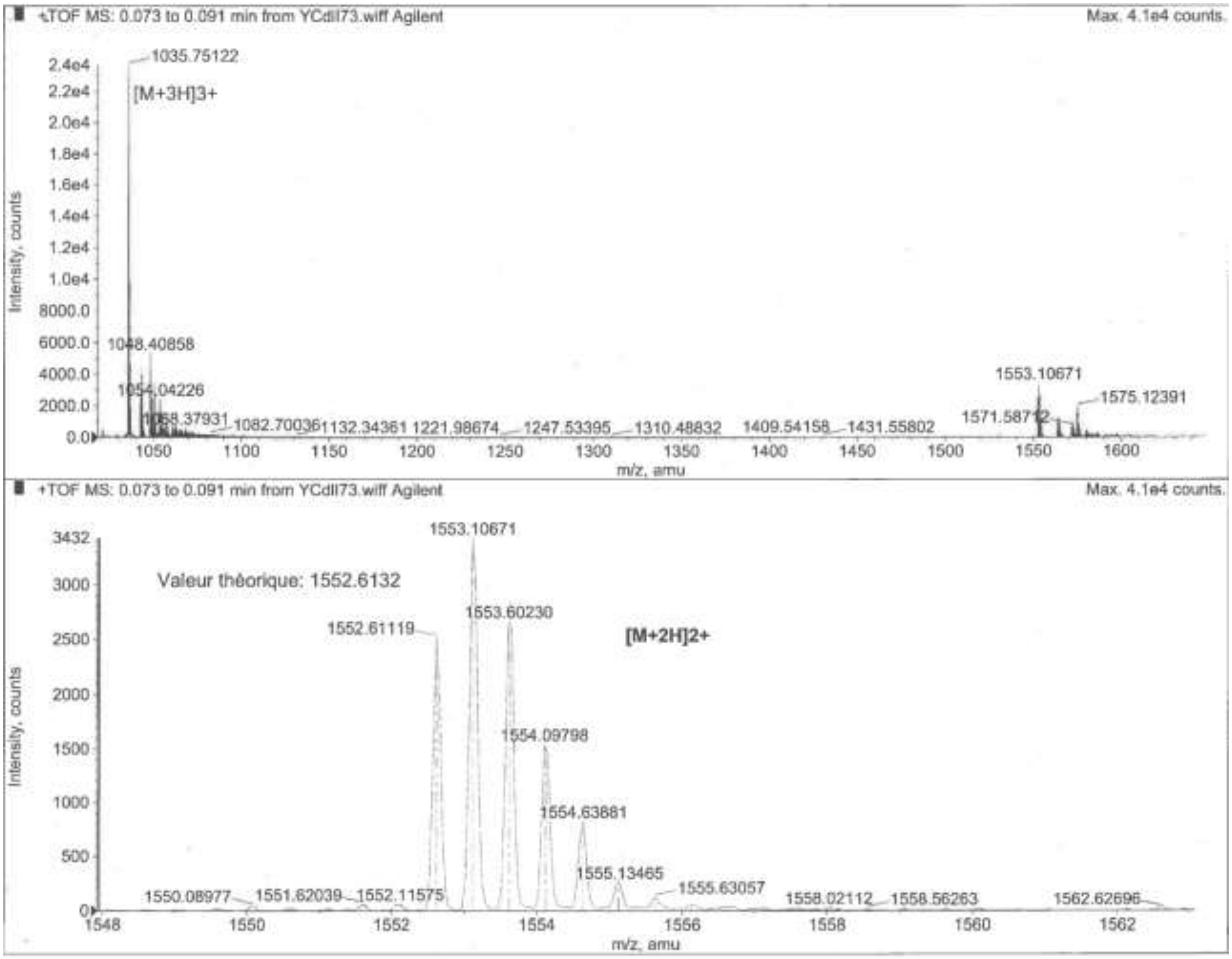

Figure S23: HRMS spectrum ($^{+}$TOF) for compound **12.**

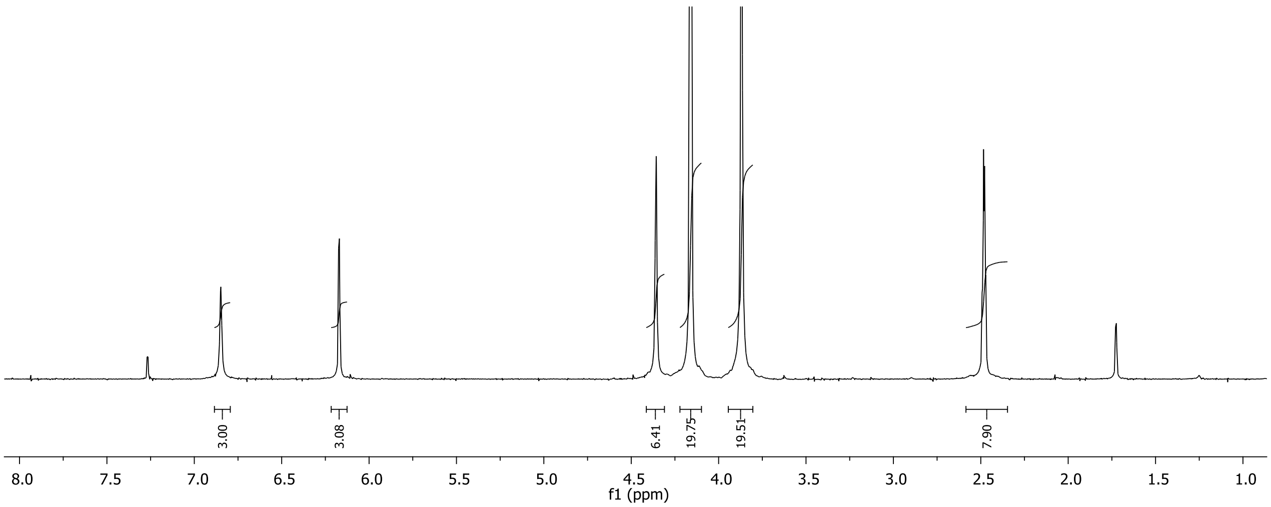

Figure S24: ^{1}H NMR spectrum of compound **15** ($CDCl_3$, 300 MHz).

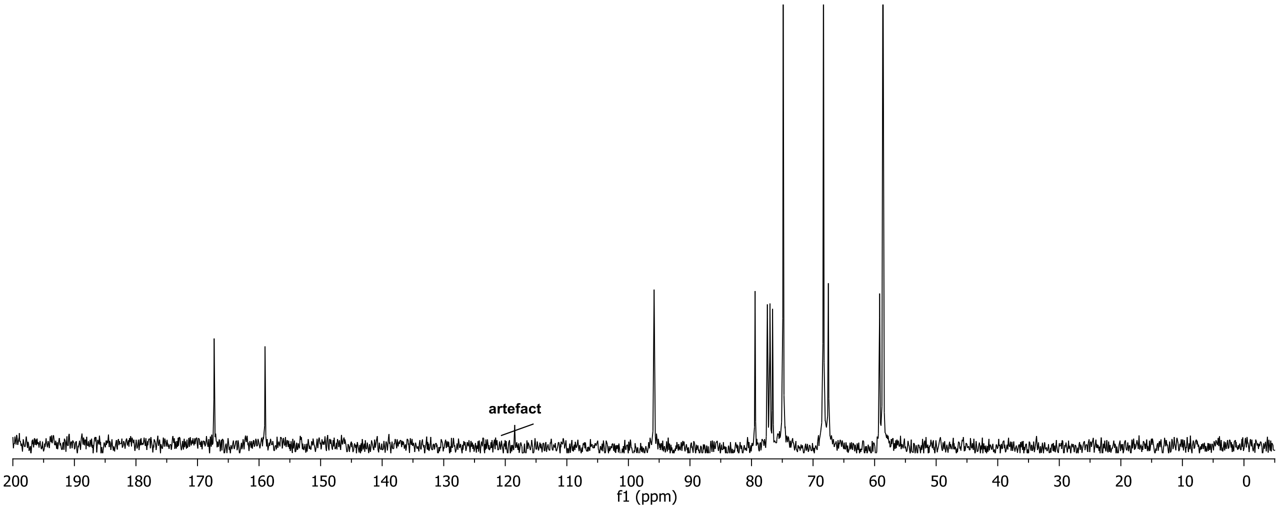

Figure S25: ^{13}C NMR spectrum of compound **15** ($CDCl_3$, 75 MHz).

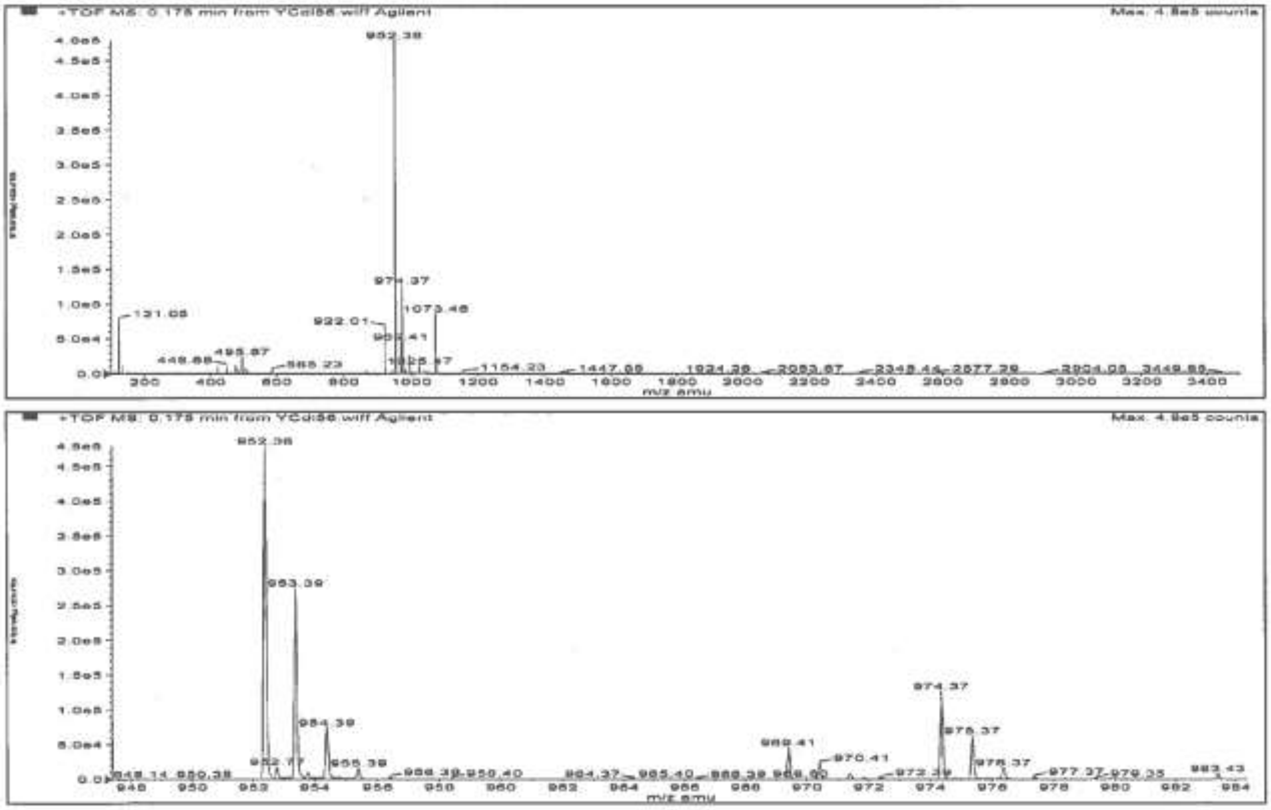

Formula	Compound name	Mass	Peak RT (min)	Peak area	Description
C51H57N3O15	--	951.37897	0.12	4.67661 E7	--

Species	Abundance (counts)	Ion Mass	Measured Mass	Error (mDa)	Error (ppm)	Ret. Time Error (min)
[M+H]+	492252.26	952.38624	952.38425	-1.99939	-2.10	--
[M+Na]+	128015.10	974.36819	974.36619	-1.99496	-2.05	--

Figure S26: HRMS ($^+$TOF) spectra and report for compound **15**.

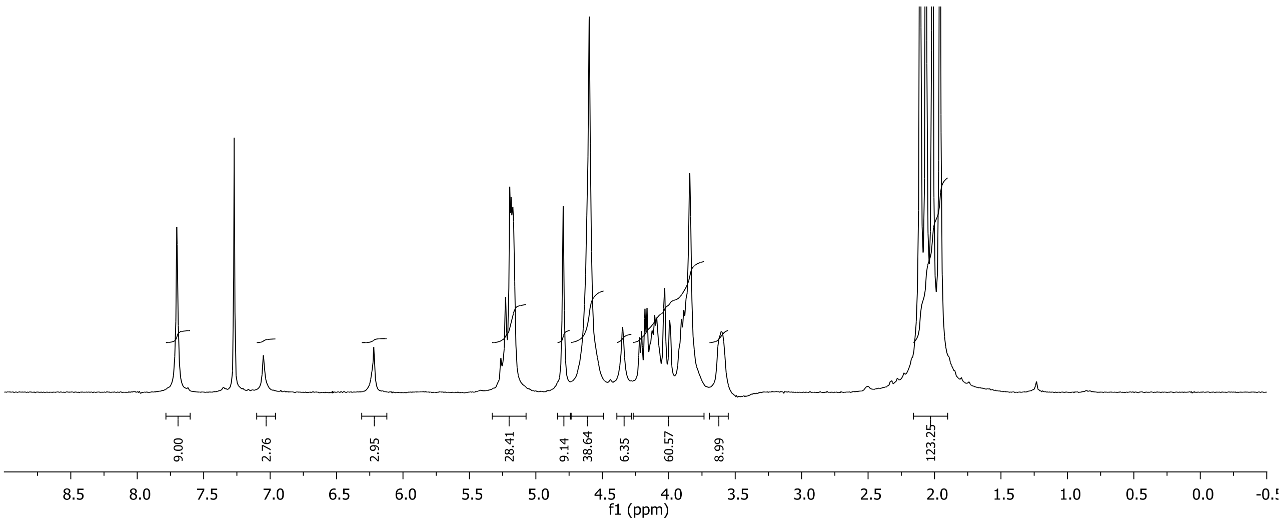

Figure S27: ^{1}H NMR spectrum of compound **16** ($CDCl_3$, 300 MHz).

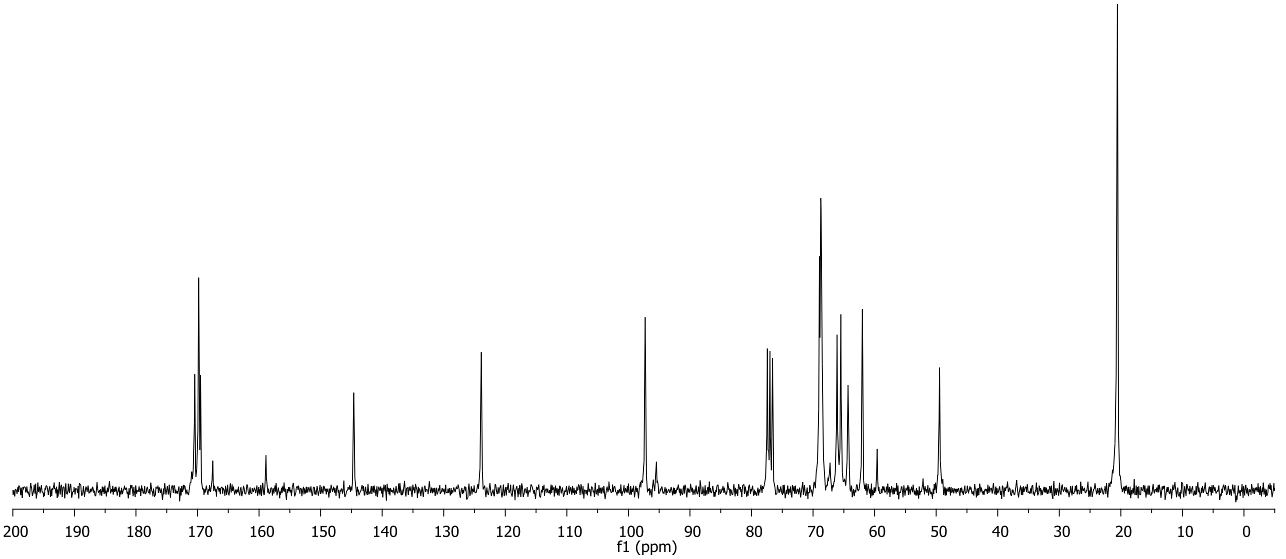

Figure S28: ^{13}C NMR spectrum of compound **16** ($CDCl_3$, 75 MHz).

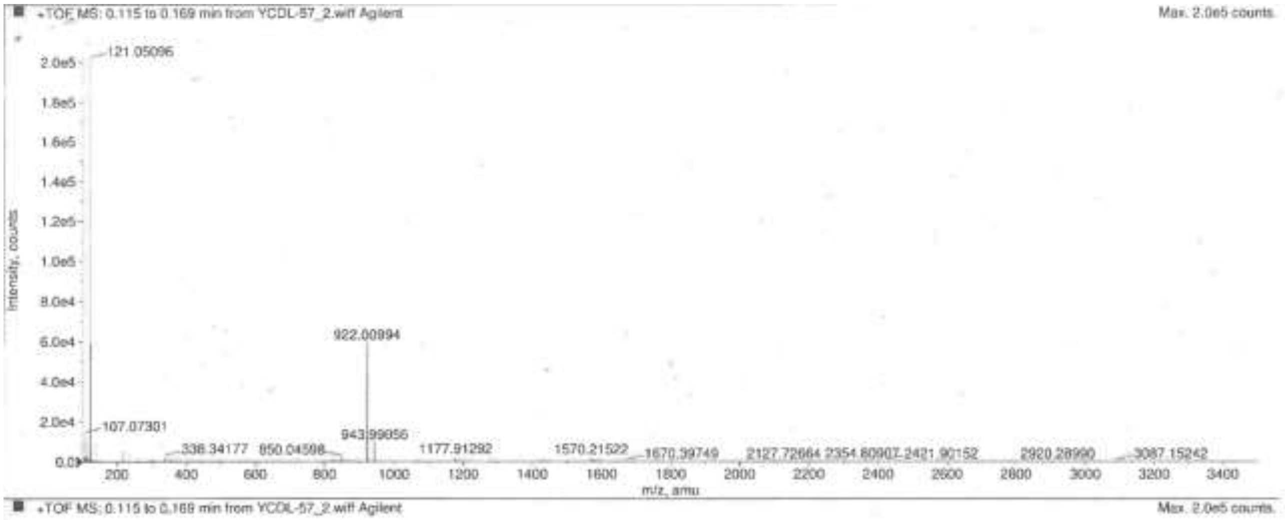

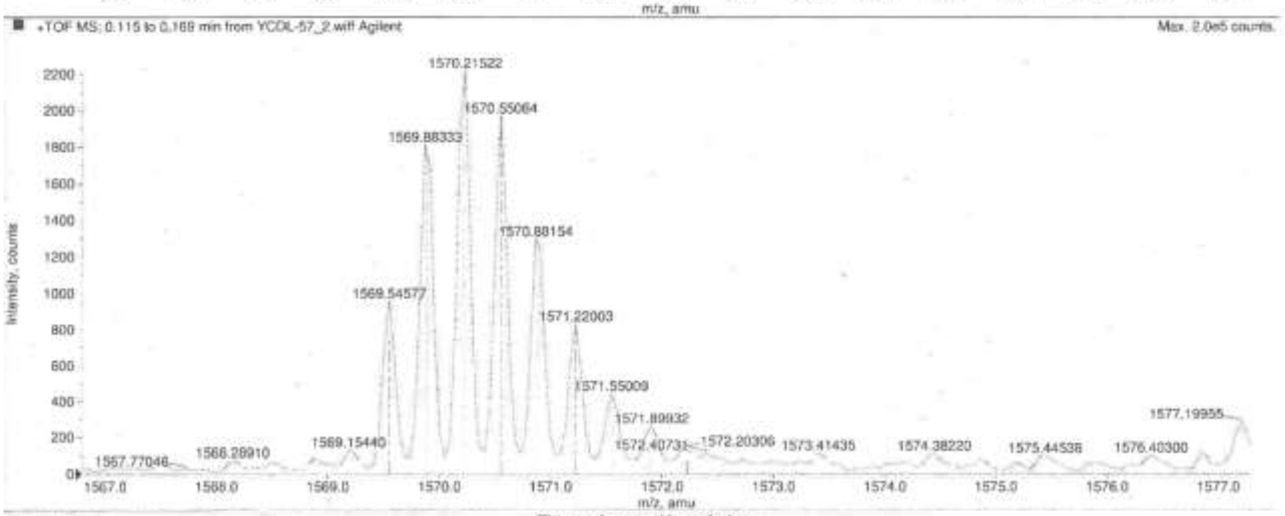

Elemental composition calculator

Target m/z: +1569.5458 amu
Tolerance: +1000.0000 ppm
Result type: Elemental
Max num of results: 500
Min DBE: -0.5000 Max DBE: +3000.0000
Electron state: OddAndEven
Num of charges: 3
Add water: N/A
Add proton: N/A
File Name: YCDL-57_2.wiff

	Elements	Min Number	Max Number
1	C	195	195
2	H	200	400
3	N	30	30
4	O	105	105

	Formula	Calculated m/z (amu)	mDa Error	PPM Error	DBE
1	C195 H267 N30 O105	1569.54863	-8.58737	-3.82374	77.5

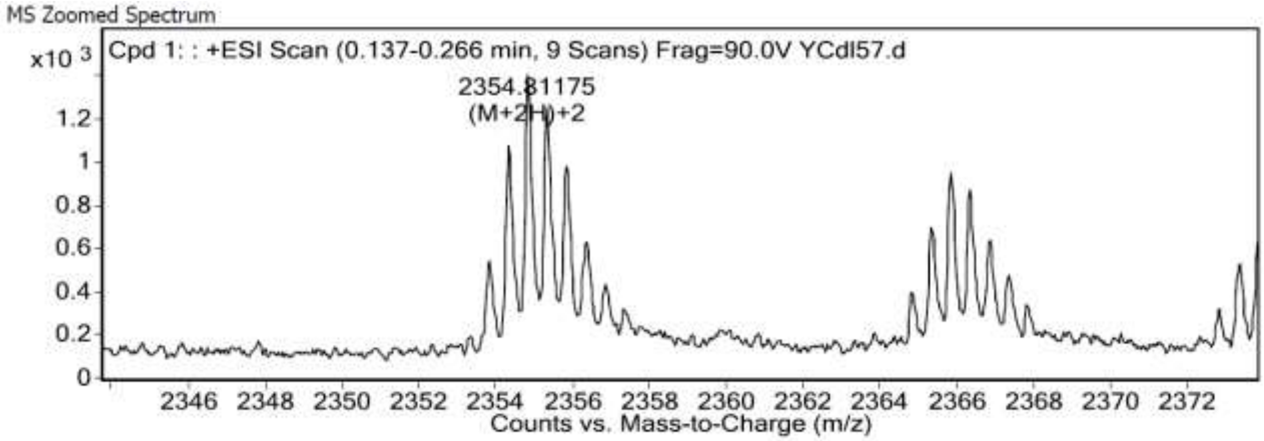

MS Spectrum Peak List

Ion	Ion Formula	Abund	*Expe. m/z*	*Calc. m/z*	Diff(ppm)
		716610.1	134.11769		
(M+2H)+2	C195H266N30O105	477.8	2353.80877	2353.81931	-4.48

Figure S29: HRMS ($^+$TOF (up) and $^+$ESI (bottom)) and reports for compound **16**.

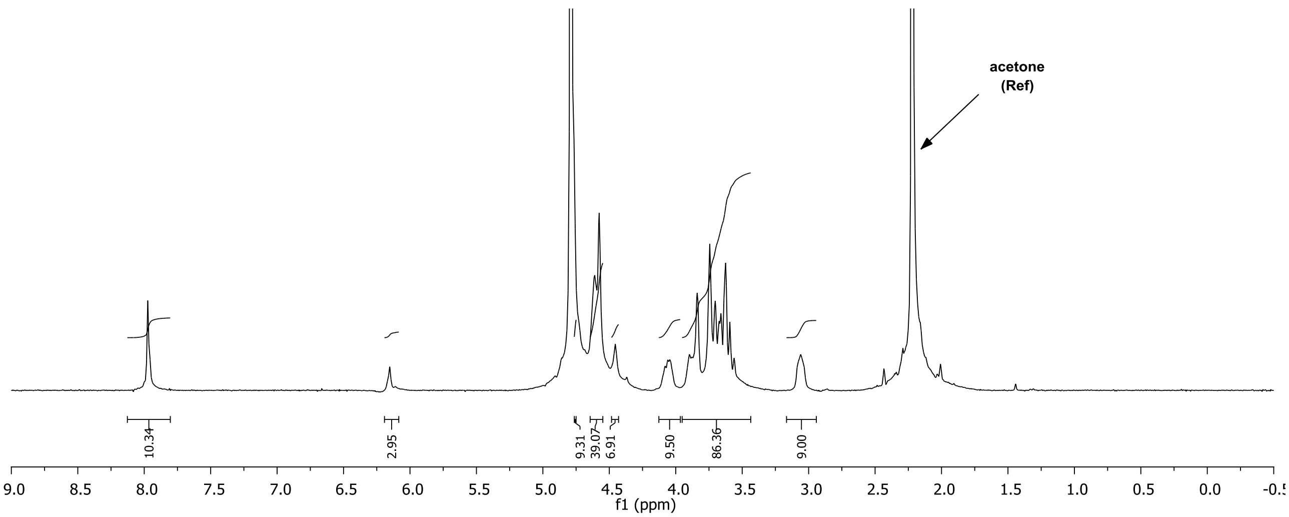

Figure S30: ^{1}H NMR spectrum of compound **17** (D_2O, 300 MHz).

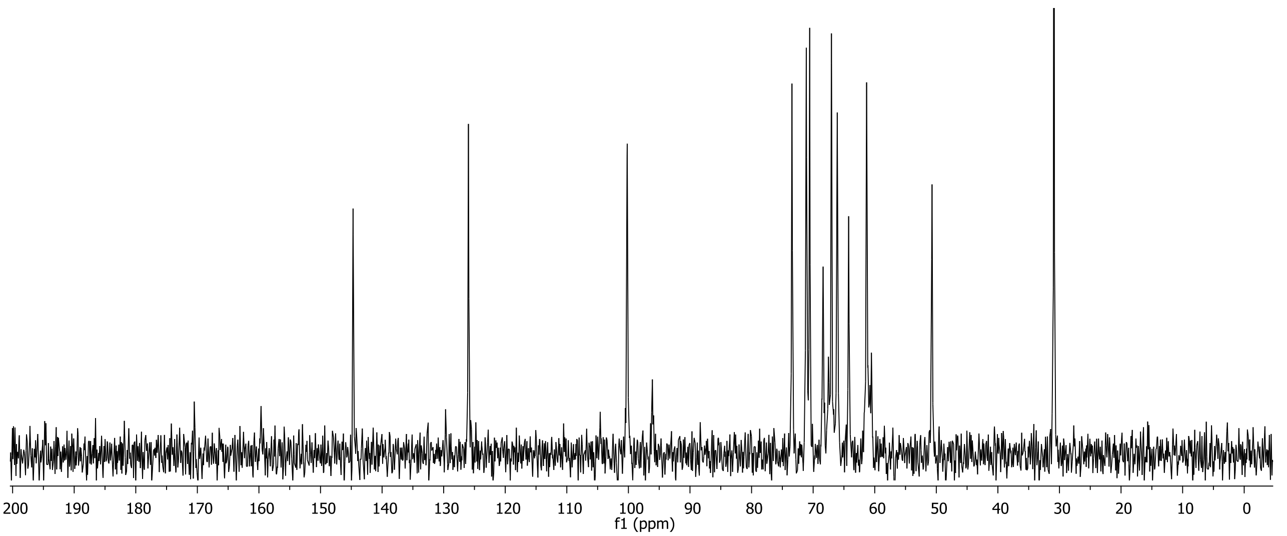

Figure S31: ^{13}C NMR spectrum of compound **17** (D_2O, 75 MHz).

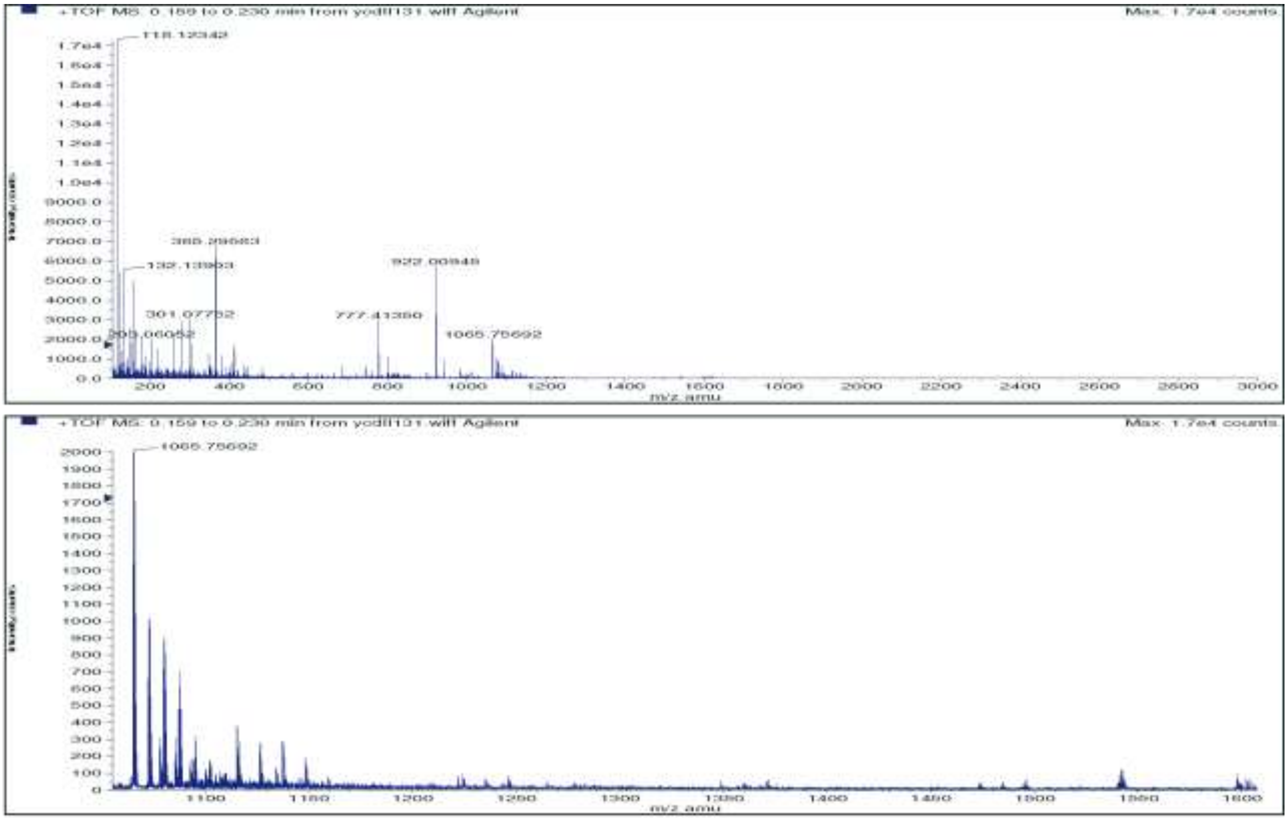

Formula	Compound name	Mass	Peak RT (min)	Peak area	Description
C123H192N30O69	--	3193.24374	0.19	1.42094 E4	--

Species	Abundance (counts)	Ion Mass	Measured Mass	Error (mDa)	Error (ppm)	Ret. Time Error (min)
[M+3H]3+	1361.61	1065.42185	1065.42210	0.24650	0.23	--

Figure S32: HRMS ($^{+}$TOF) spectrum for compound **17**.

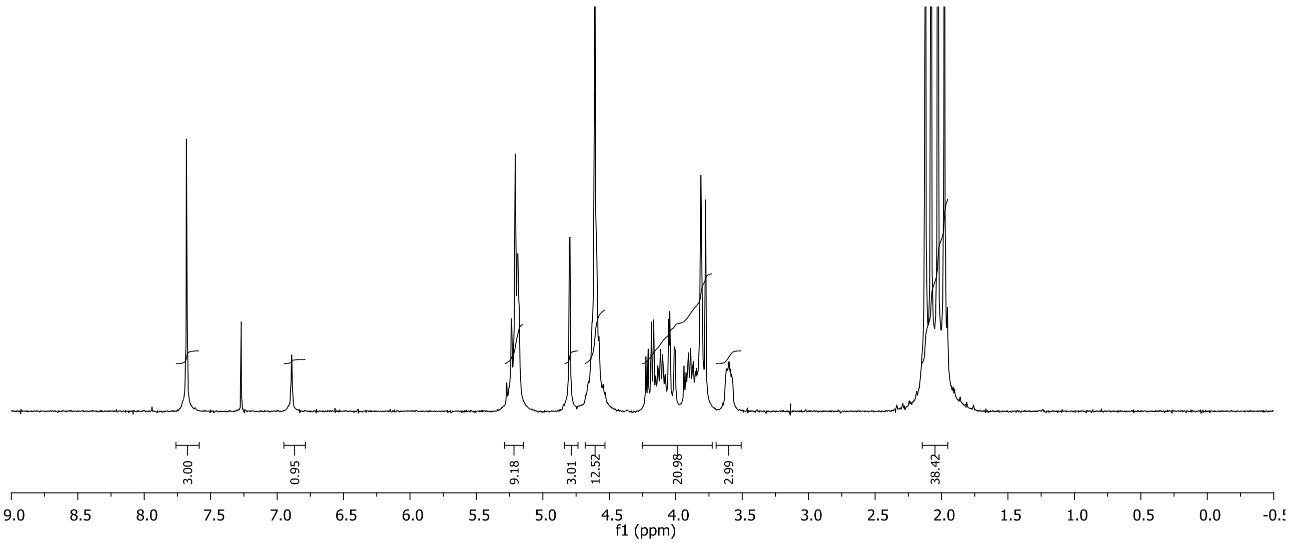

Figure S33: ^{1}H NMR spectrum of compound **18** ($CDCl_3$, 300 MHz).

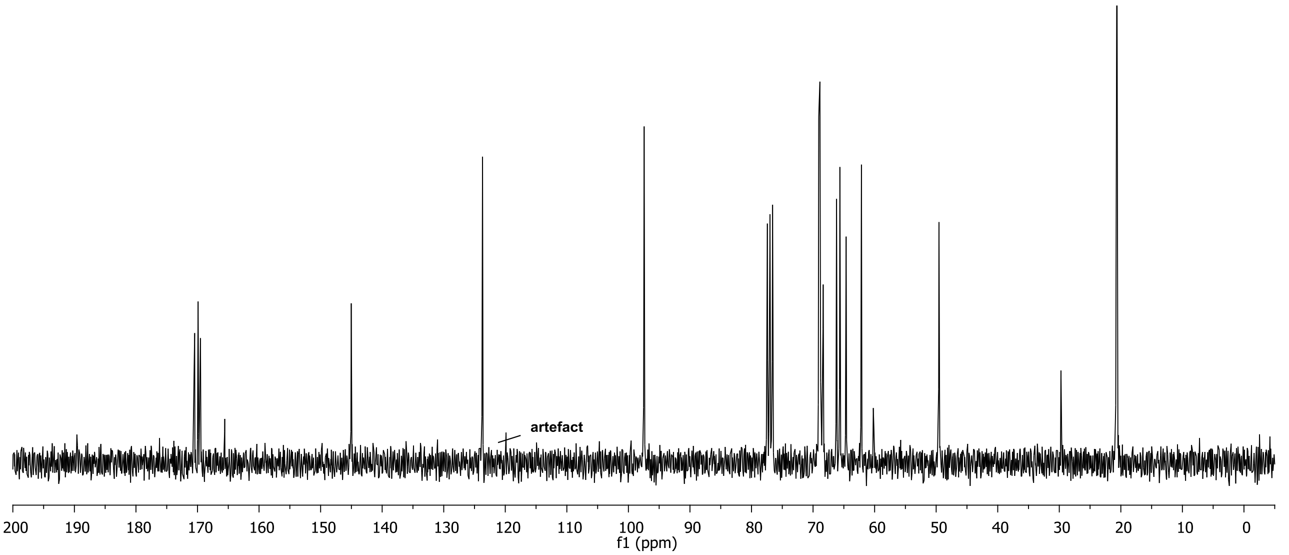

Figure S34: ^{13}C NMR spectrum of compound **18** ($CDCl_3$, 75 MHz).

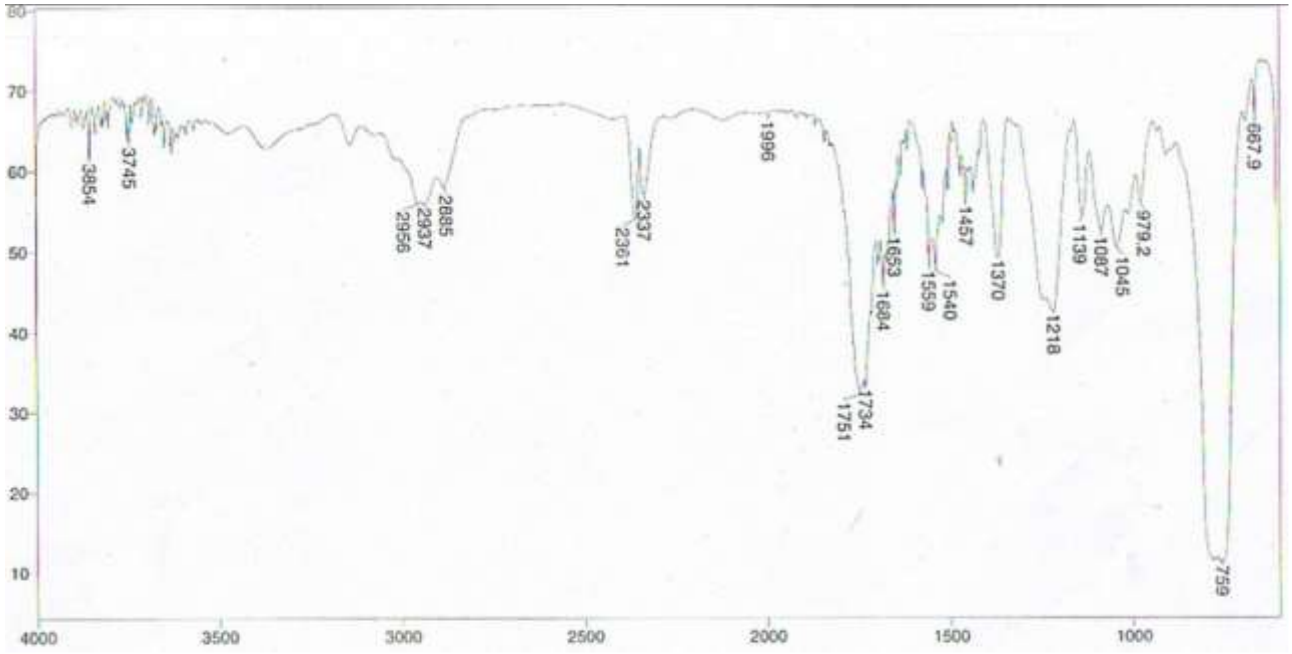

Figure S35: IR spectrum of compound **18**.

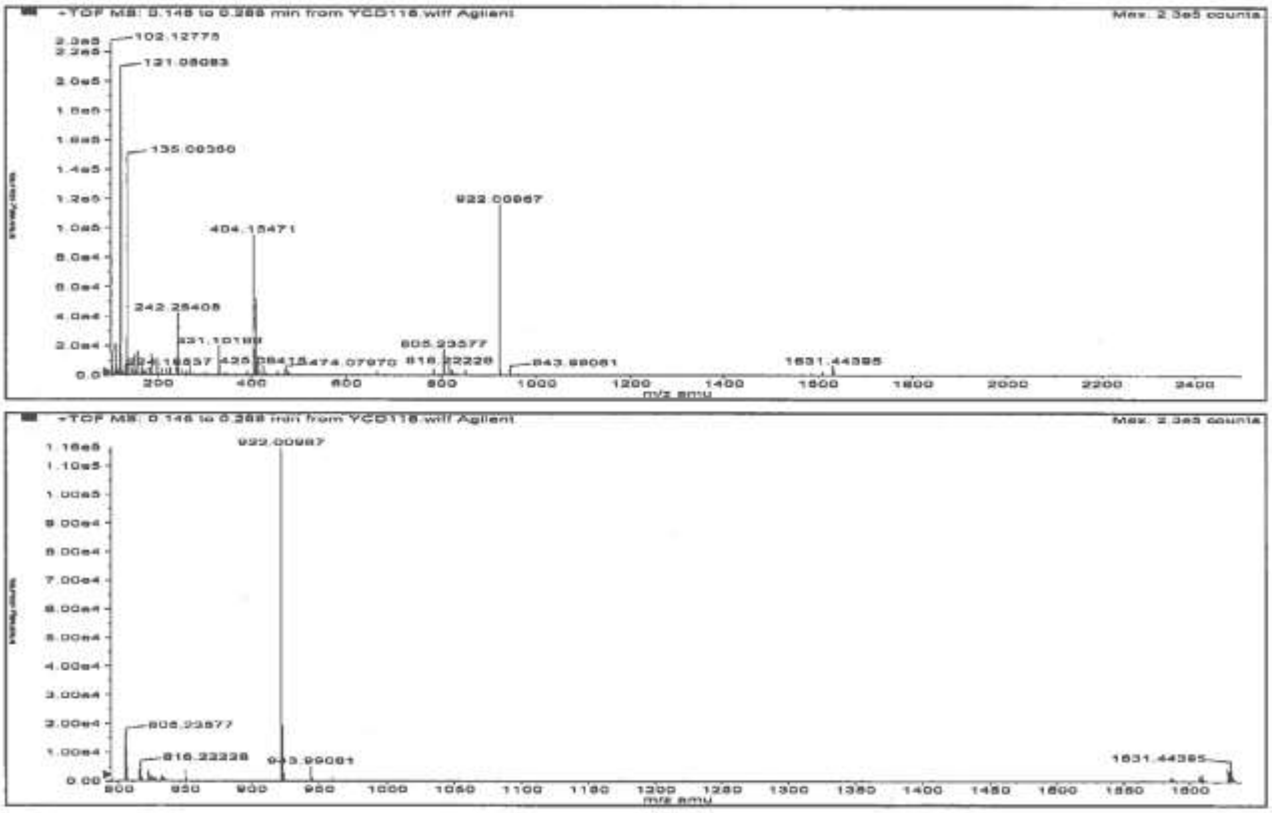

Formula	Compound name	Mass	Peak RT (min)	Peak area	Description
C63H87BrN10O34	--	1606.45695	0.18	5.45742 E5	--

Species	Abundance (counts)	Ion Mass	Measured Mass	Error (mDa)	Error (ppm)	Ret. Time Error (min)
[M+2H]2+	14787.14	804.23575	804.23561	-0.14429	-0.18	--
[M+H]+	2036.89	1607.46423	1607.46204	-2.19006	-1.36	--
[M+Na]+	4716.23	1629.44617	1629.44480	-1.37214	-0.84	--

Figure S36: HRMS ($^{+}$TOF) spectrum and report for compound **18**.

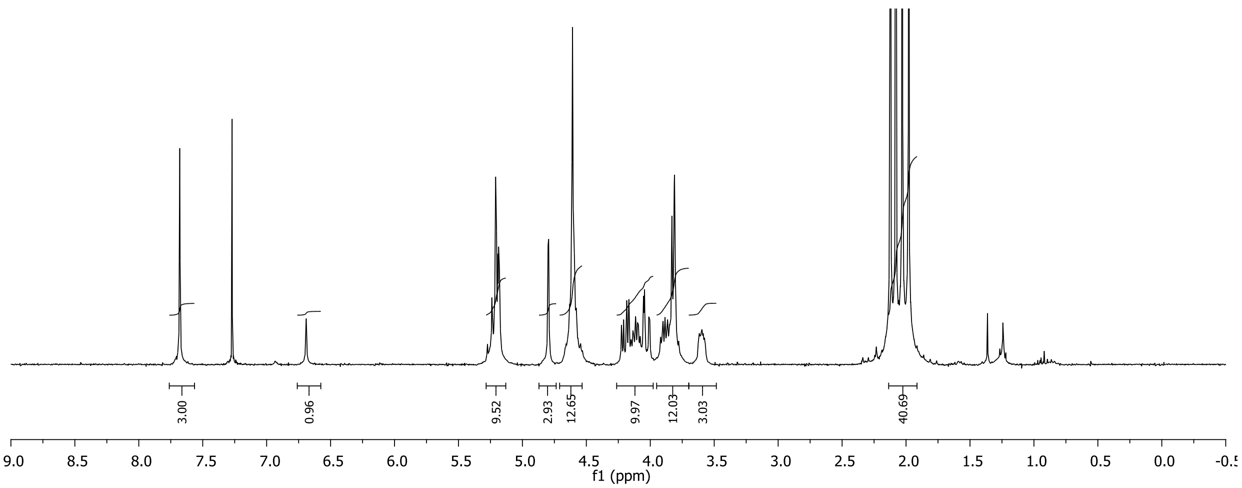

Figure S37: ^{1}H NMR spectrum of compound **19** ($CDCl_3$, 300 MHz).

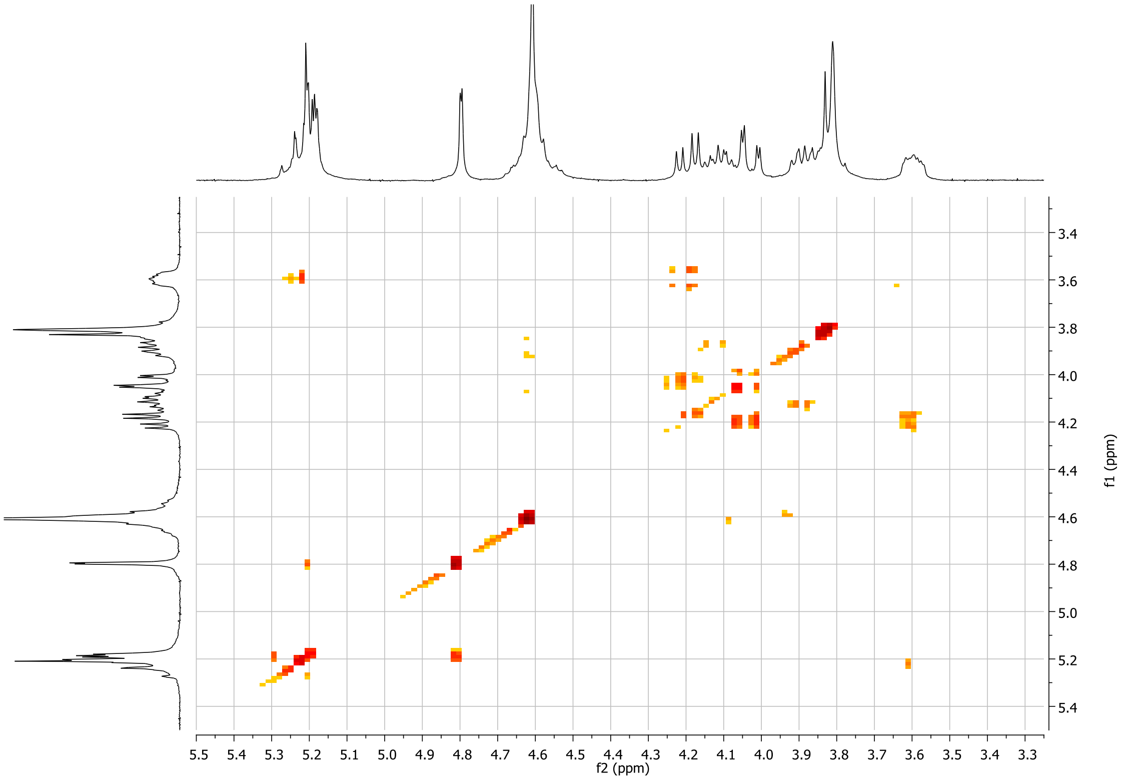

Figure S38: COSY spectrum of compound **19**.

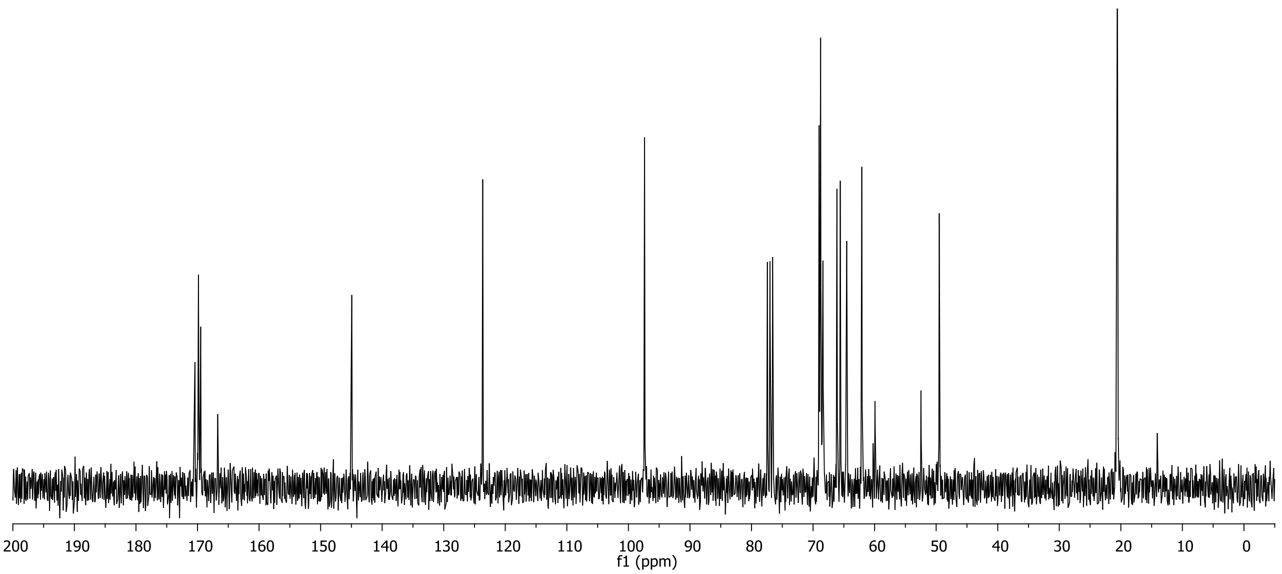

Figure S39: ^{13}C NMR spectrum of compound **19** ($CDCl_3$, 75 MHz).

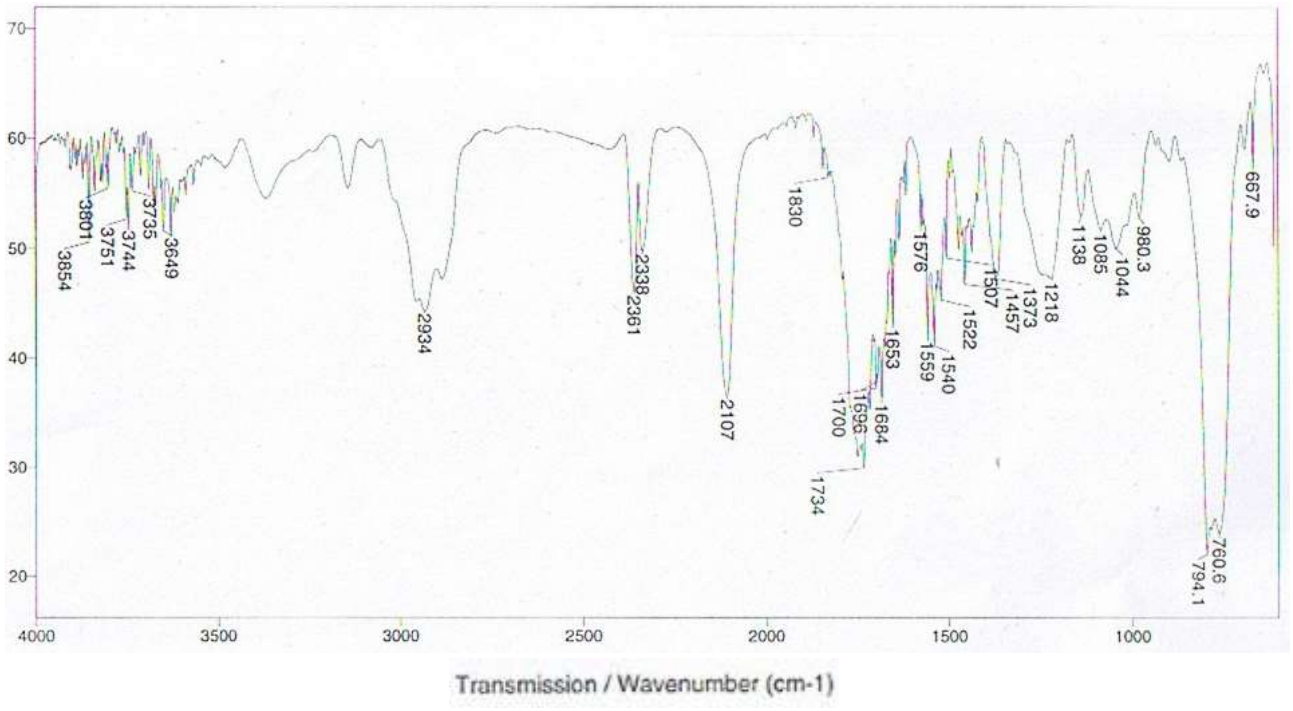

Figure S40: IR spectrum of compound **19**.

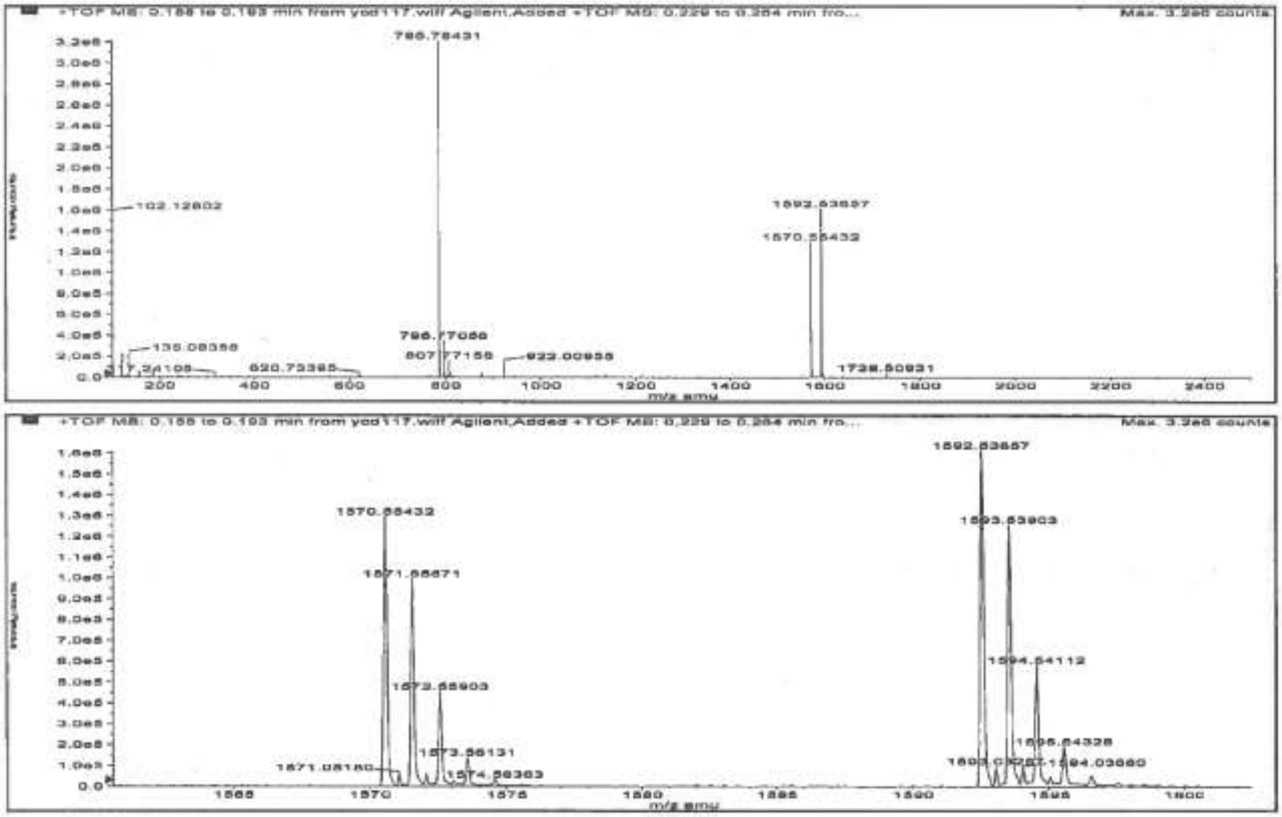

Formula	Compound name	Mass	Peak RT (min)	Peak area	Description
C63H87N13O34	--	1569.54784	0.19	3.68551 E7	--

Species	Abundance (counts)	Ion Mass	Measured Mass	Error (mDa)	Error (ppm)	Ret. Time Error (min)
[M+H]+	1300659.76	1570.55511	1570.55432	-0.79404	-0.51	--
[M+Na]+	1666530.91	1592.53706	1592.53657	-0.49272	-0.31	--

Figure S41: HRMS ($^{+}$TOF) trace and report for compound **19**.

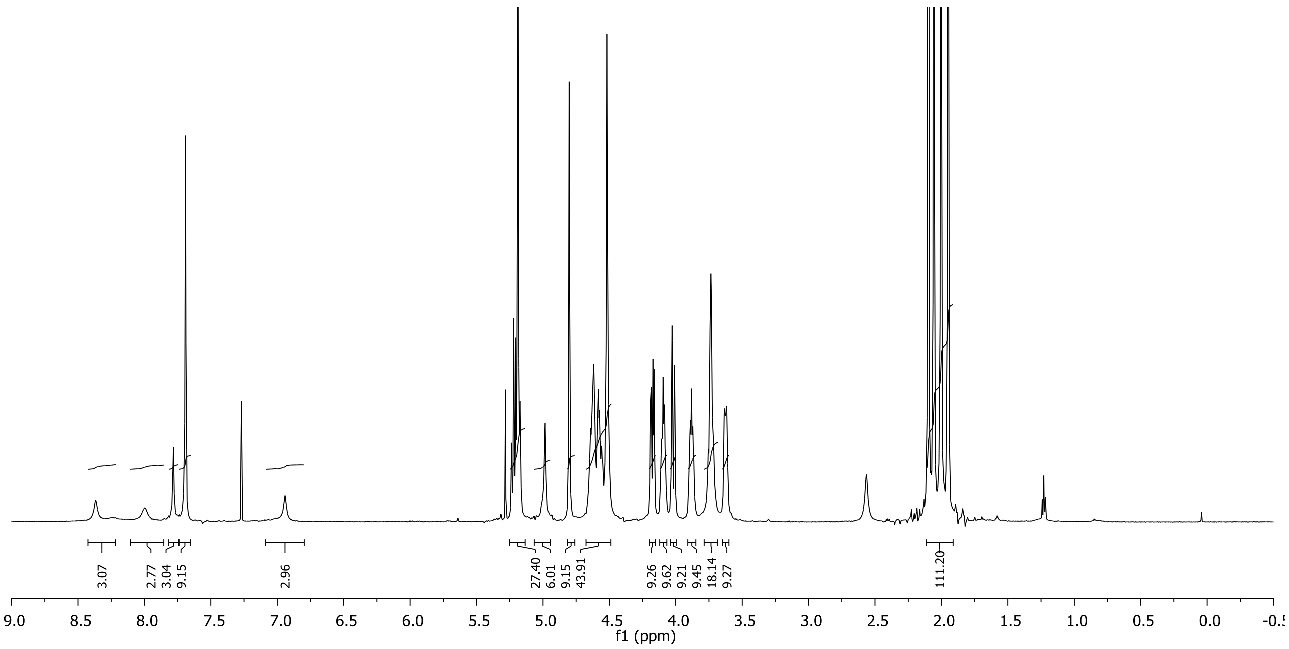

Figure S42: ^{1}H NMR spectrum of compound **20** ($CDCl_3$, 600 MHz).

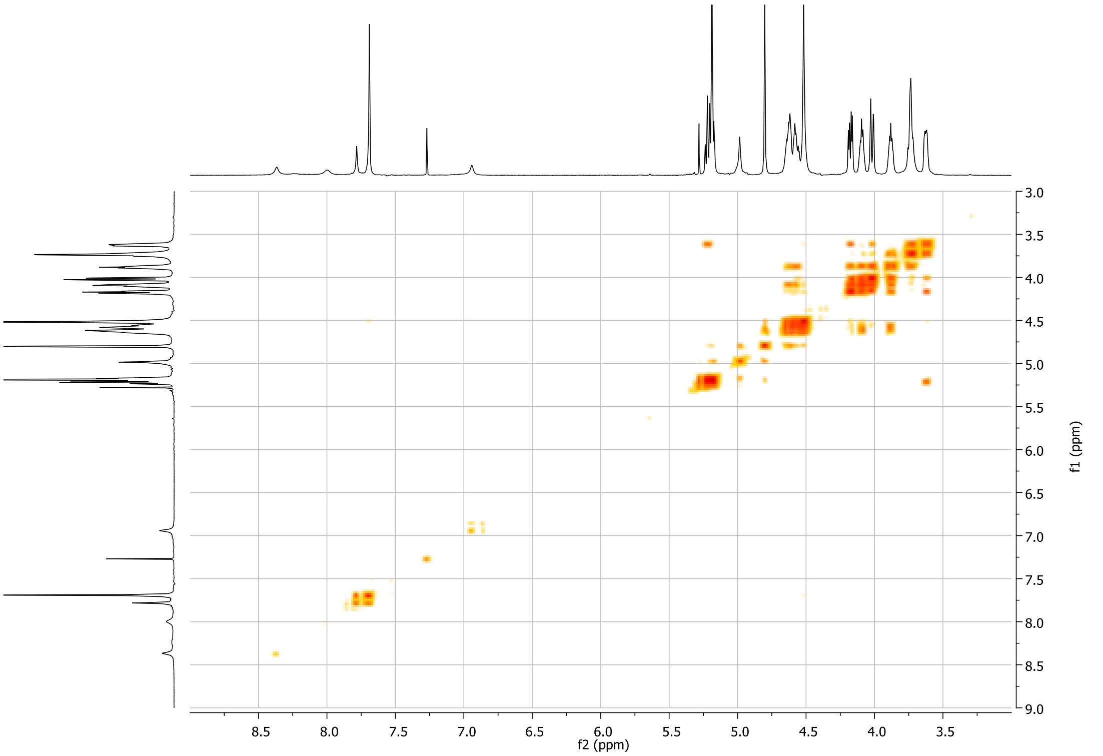

Figure S43: COSY spectrum of compound **20**.

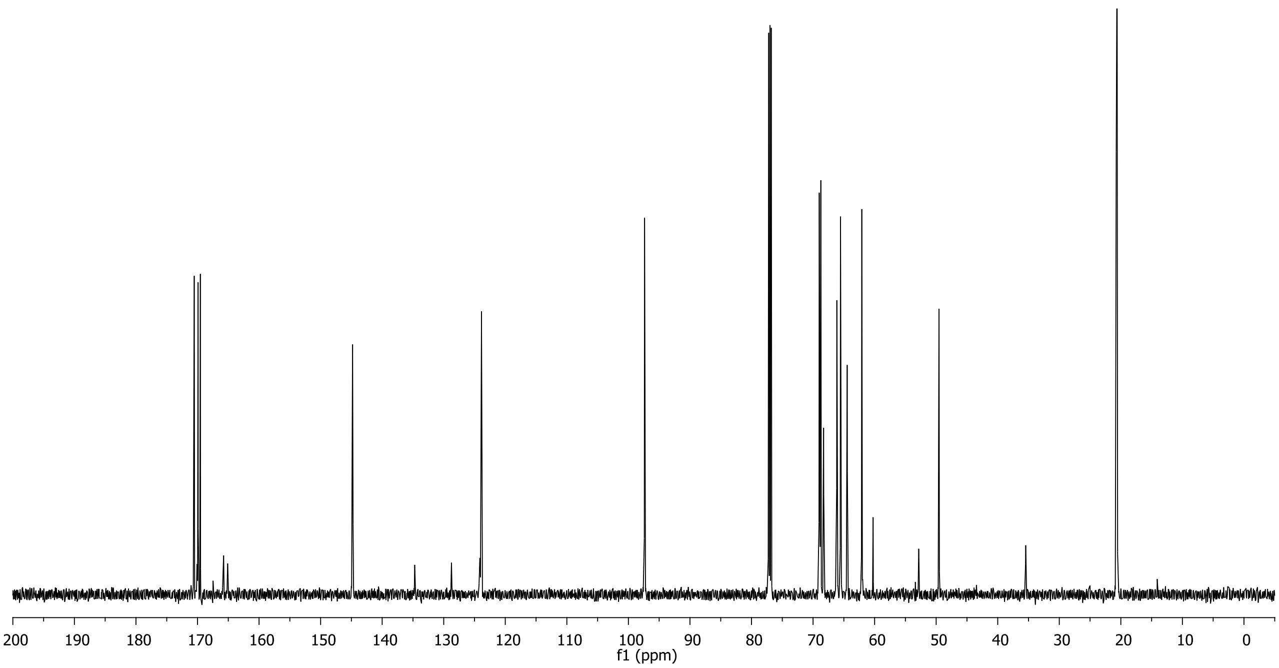

Figure S44: ^{13}C NMR spectrum of compound **20** ($CDCl_3$, 150 MHz).

MS Spectrum

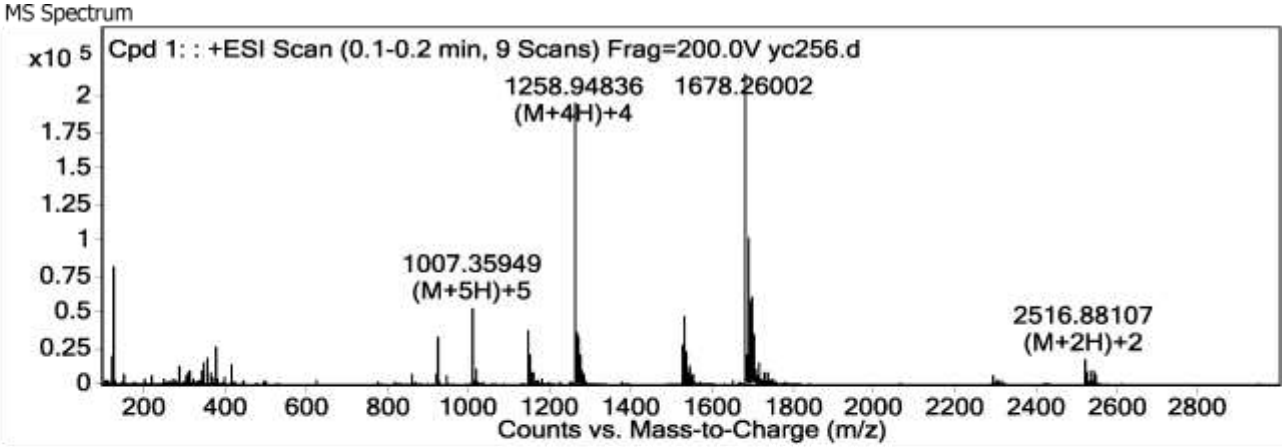

MS Zoomed Spectrum

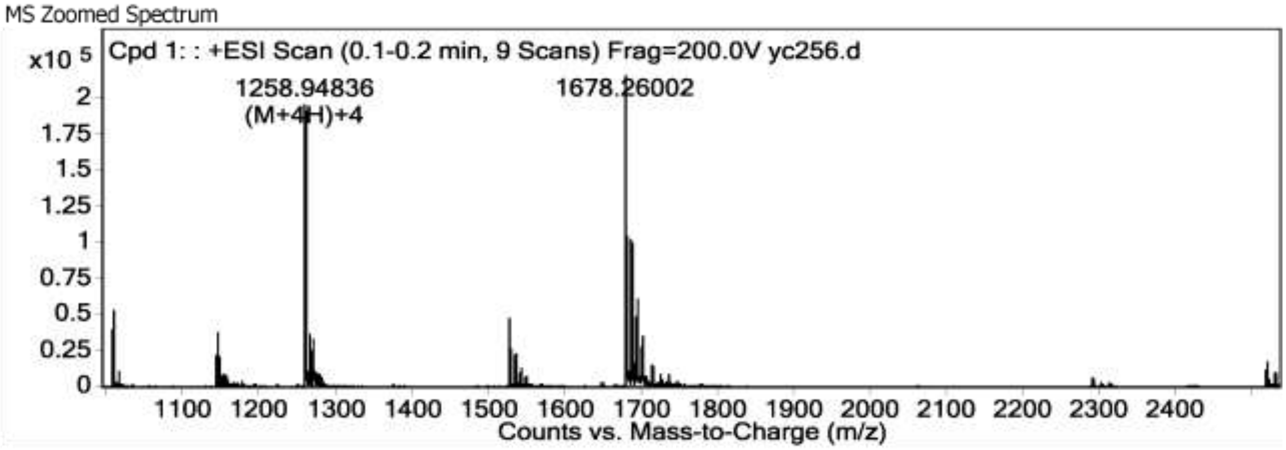

MS Spectrum Peak List

Ion	Ion Formula	Abund	*Expe. m/z*	*Calc. m/z*	Diff(ppm)
(M+5H)+5	C207H281N42O105	16249.9	1006.96133	1006.95825	3.06
(M+4H)+4	C207H280N42O105	56590.4	1258.44891	1258.44599	2.32
(M+3H)+3	C207H279N42O105	60597.4	1677.59355	1677.59223	0.79
(M+2H)+2	C207H278N42O105	4401.5	2515.88451	2515.8847	-0.08

Figure S45: HRMS ($^+$ESI) spectrum and report for compound **20**.

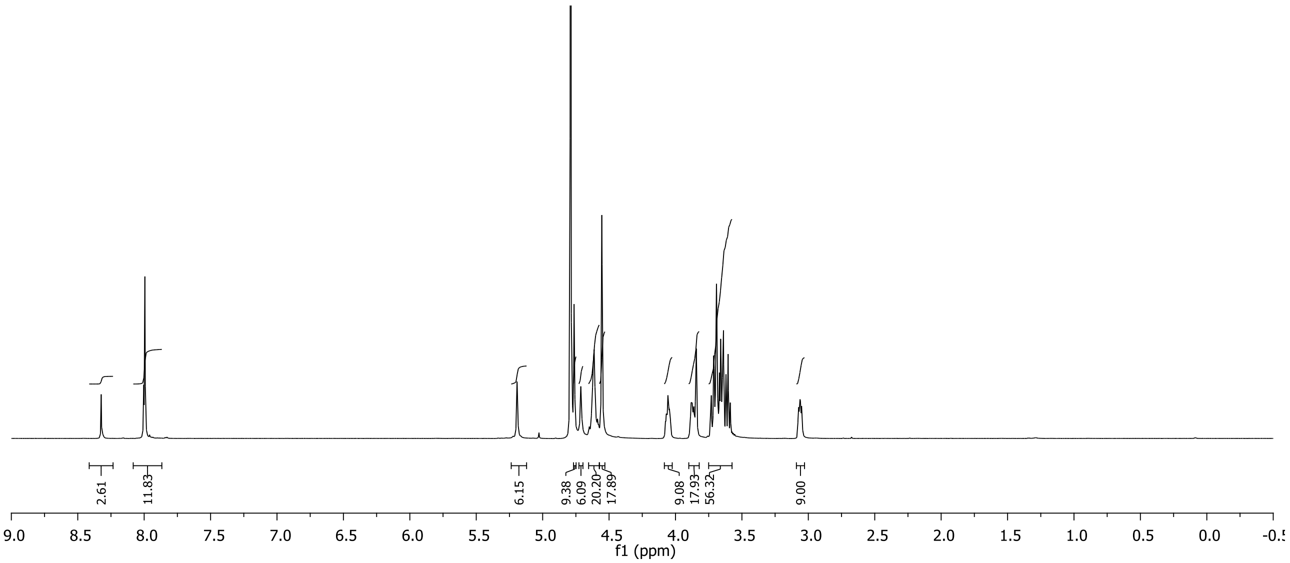

Figure S46: ^{1}H NMR spectrum of compound **21** (D_2O, 600 MHz).

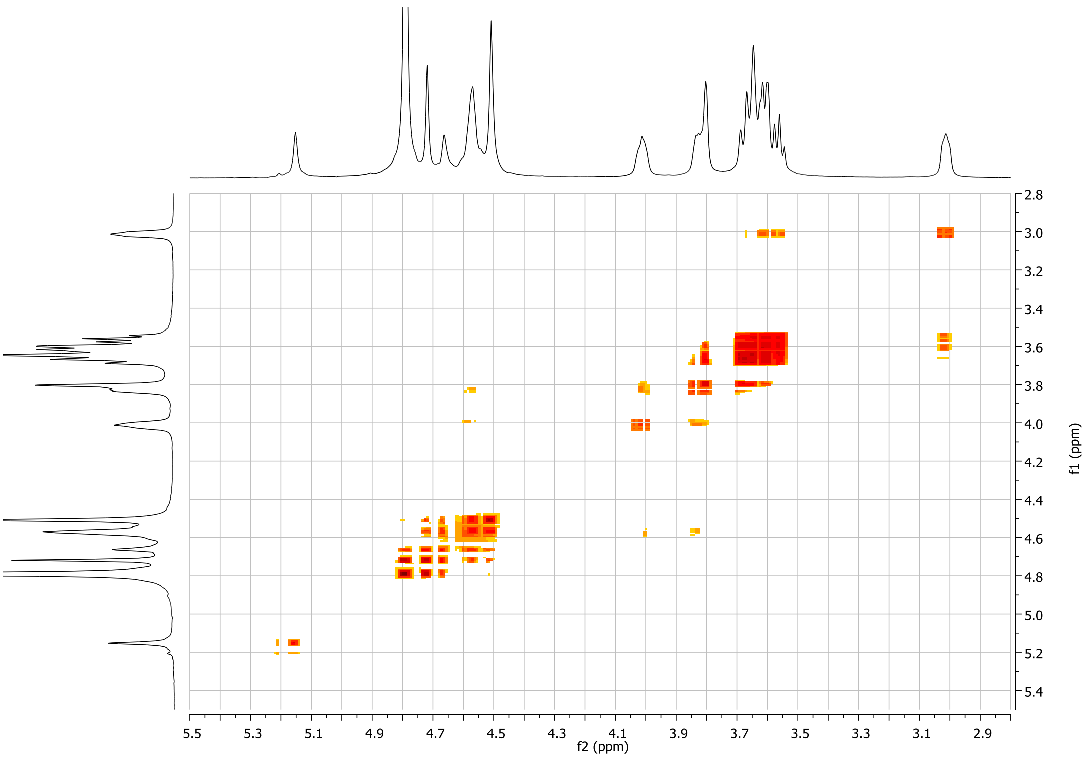

Figure S47: COSY spectrum (zoom) of compound **21**.

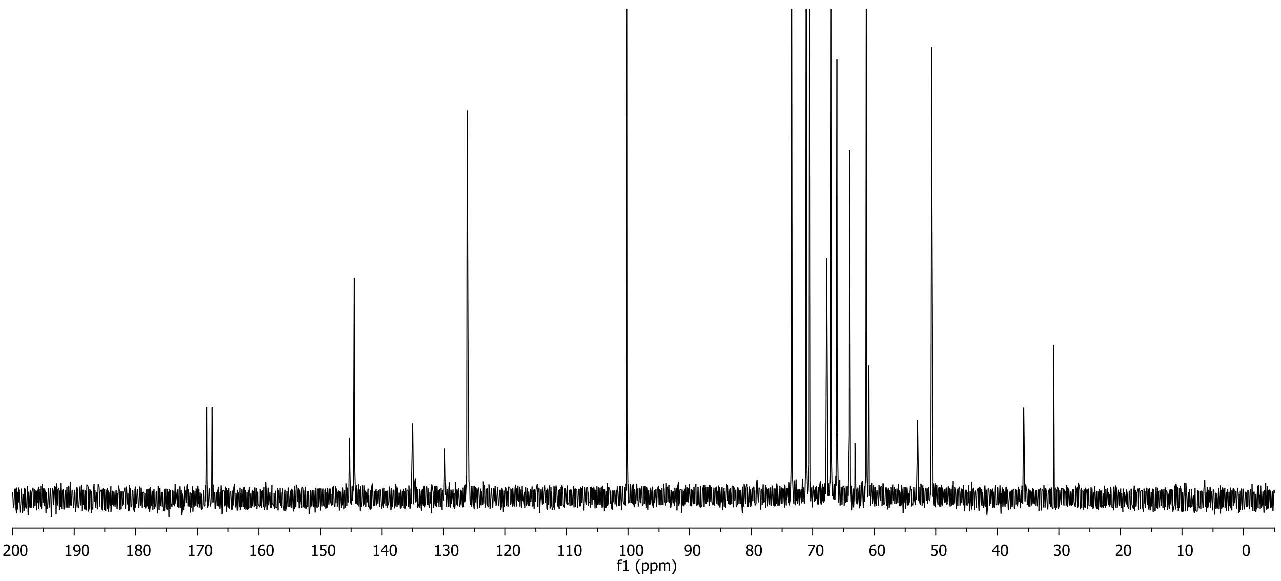

Figure S48: ^{13}C NMR spectrum of compound **21** (D_2O, 150 MHz).

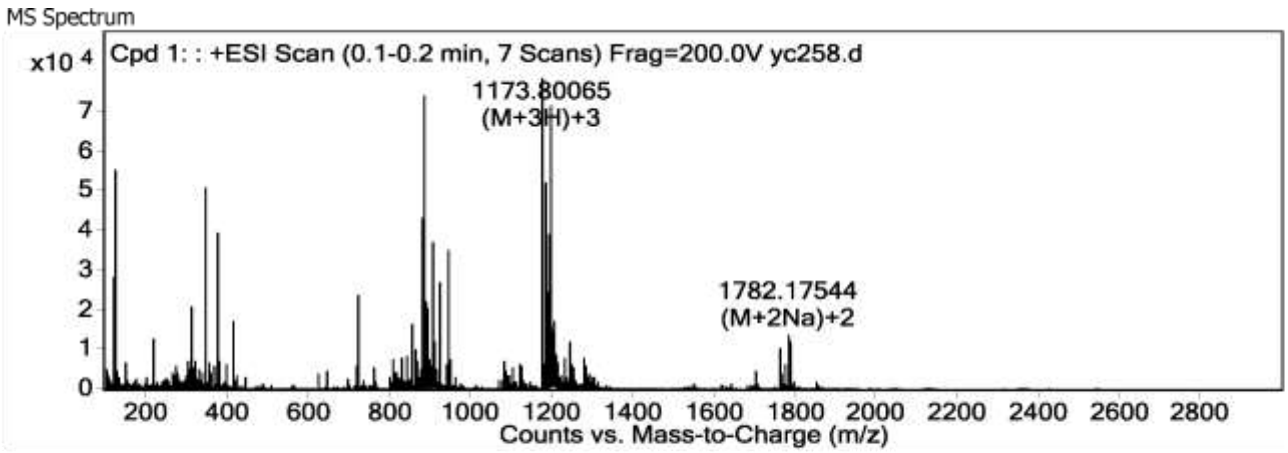

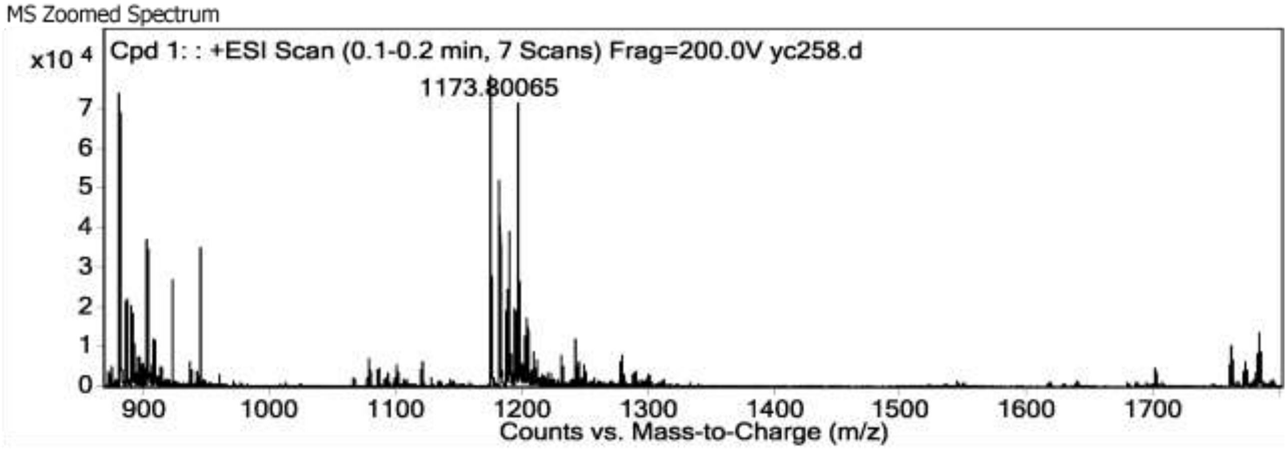

MS Spectrum Peak List

Ion	Ion Formula	Abund	*Expe. m/z*	*Calc. m/z*	Diff(ppm)
(M+4H)+4	C135H208N42O69	43132.3	880.3535	880.35091	2.95
(M+4Na)+4	C135H204N42Na4O69	21932.4	902.33583	902.33285	3.3
(M+3H)+3	C135H207N42O69	45279.6	1173.46714	1173.46545	1.44
(M+3Na)+3	C135H204N42Na3O69	42774.8	1195.44896	1195.4474	1.31
(M+2H)+2	C135H206N42O69	5484.3	1759.69044	1759.69454	-2.33
(M+2Na)+2	C135H204N42Na2O69	8555.1	1781.67369	1781.67648	-1.57

Figure S49: HRMS ($^{+}$ESI) spectrum of compound **21**.

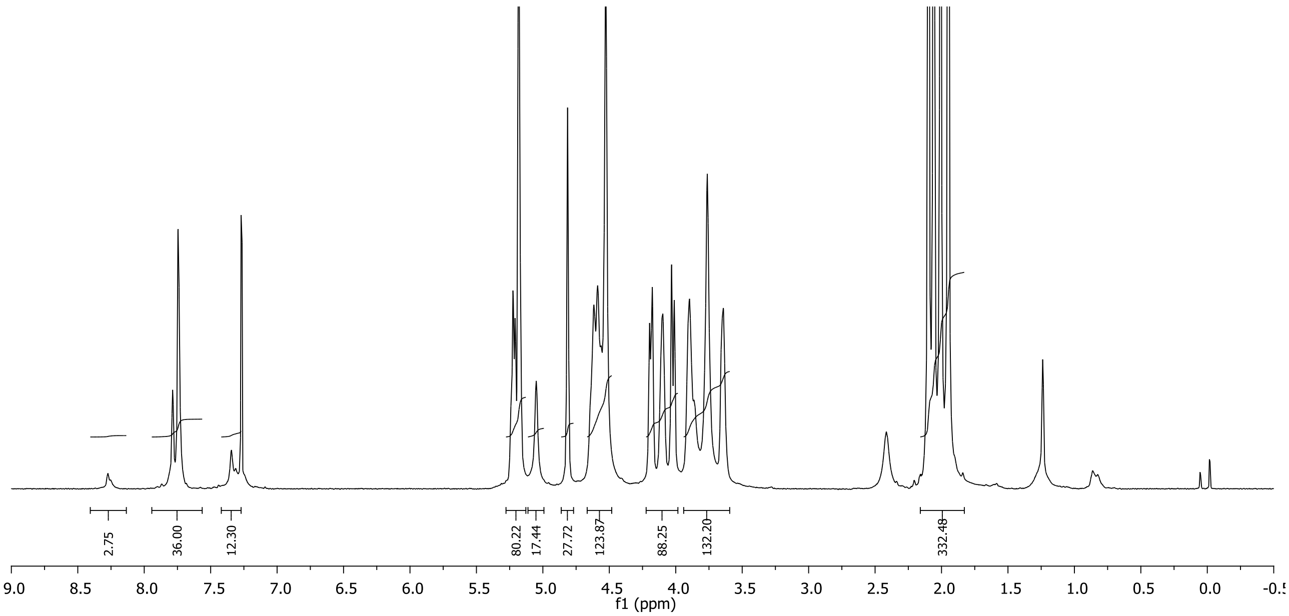

Figure S50: ^{1}H NMR spectrum of compound **22** ($CDCl_3$, 600 MHz).

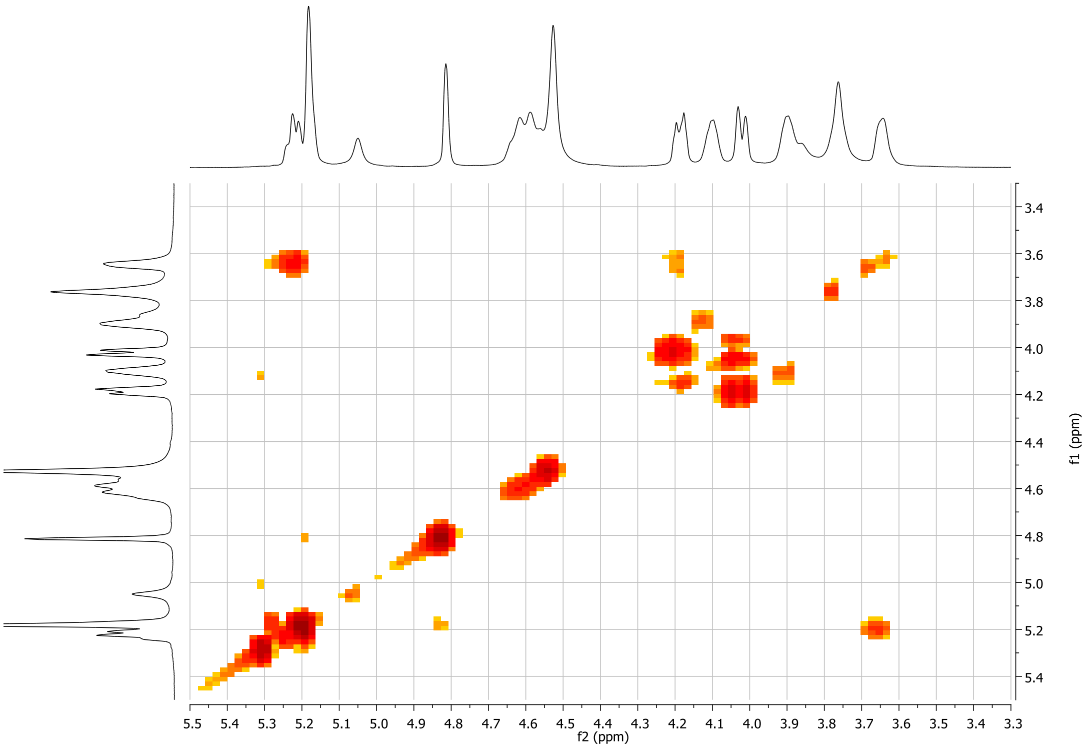

Figure S51: COSY spectrum of compound **22**.

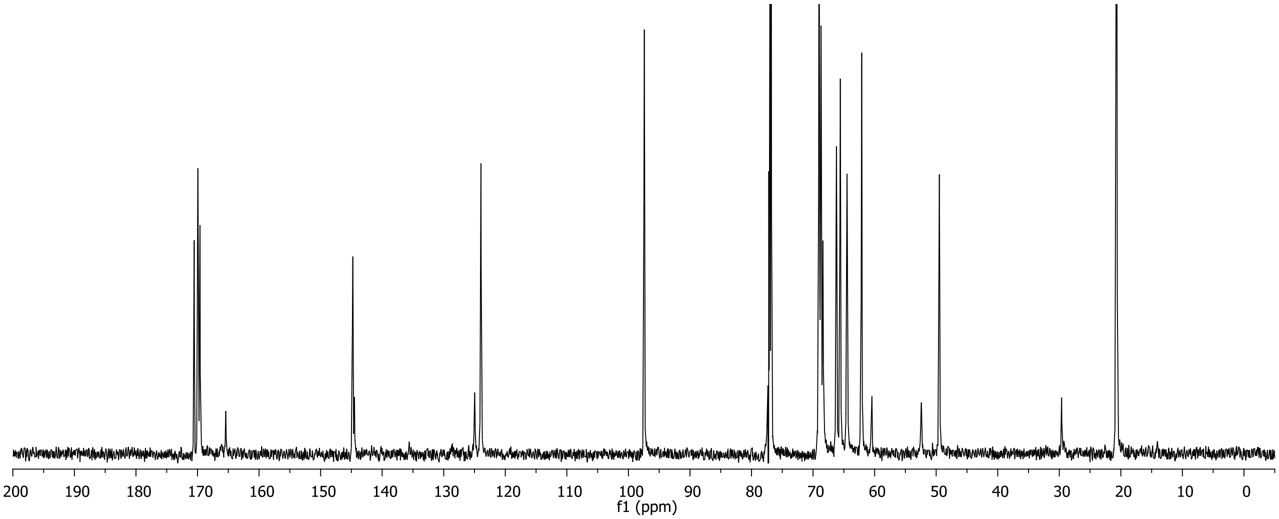

Figure S52: ^{13}C NMR spectrum of compound **22** ($CDCl_3$, 150 MHz).

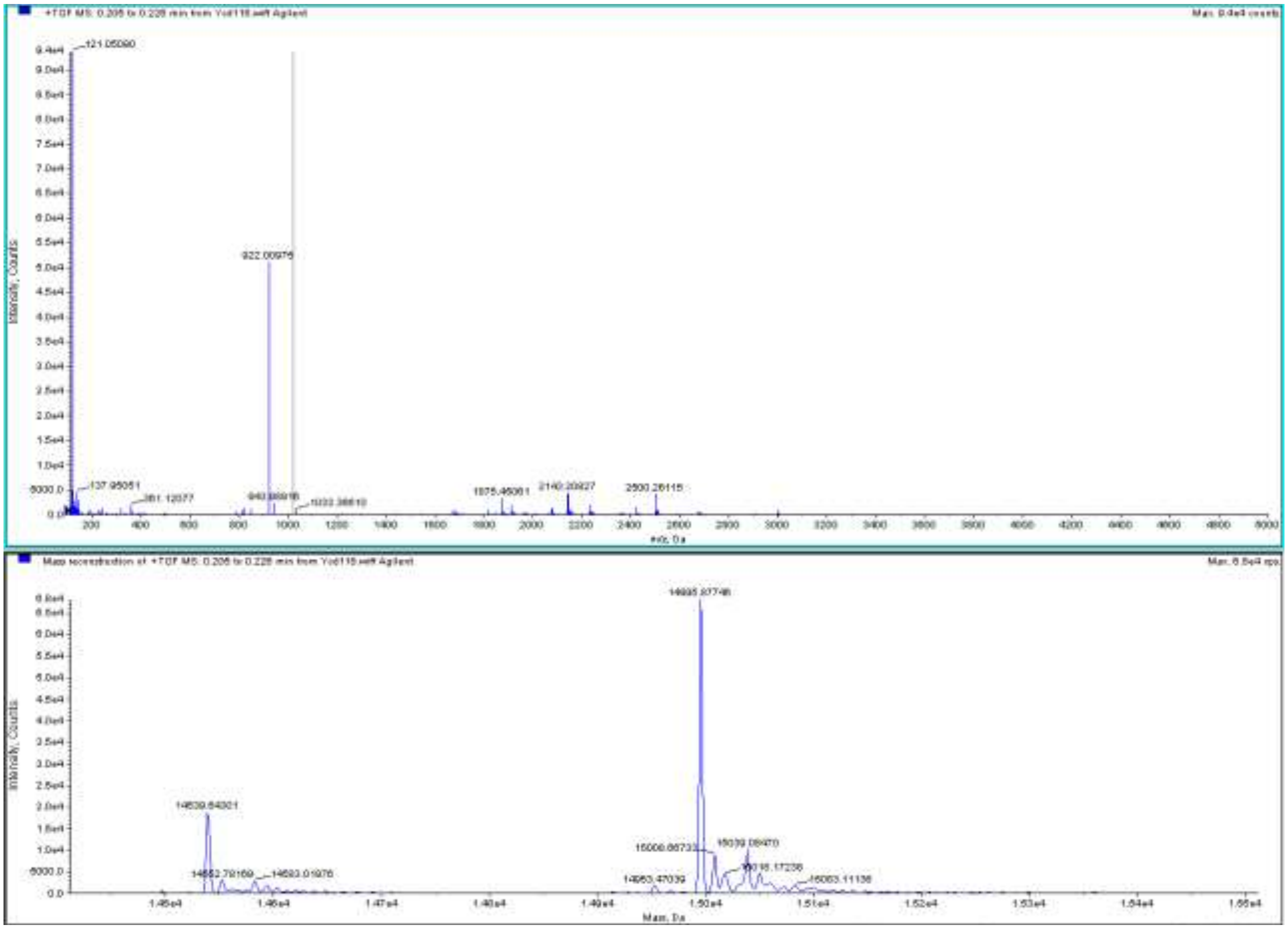

Figure S53: MS ($^+$TOF) spectrum of compound **22**.

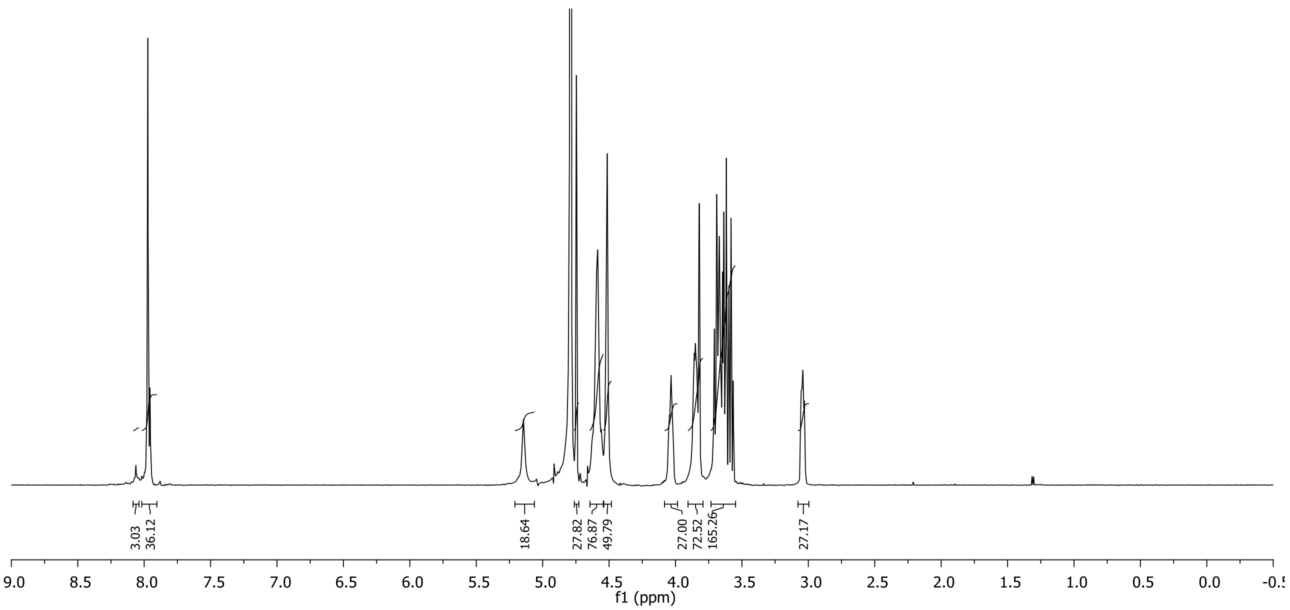

Figure S54: ^{1}H NMR spectrum of compound **23** (D_2O, 600 MHz).

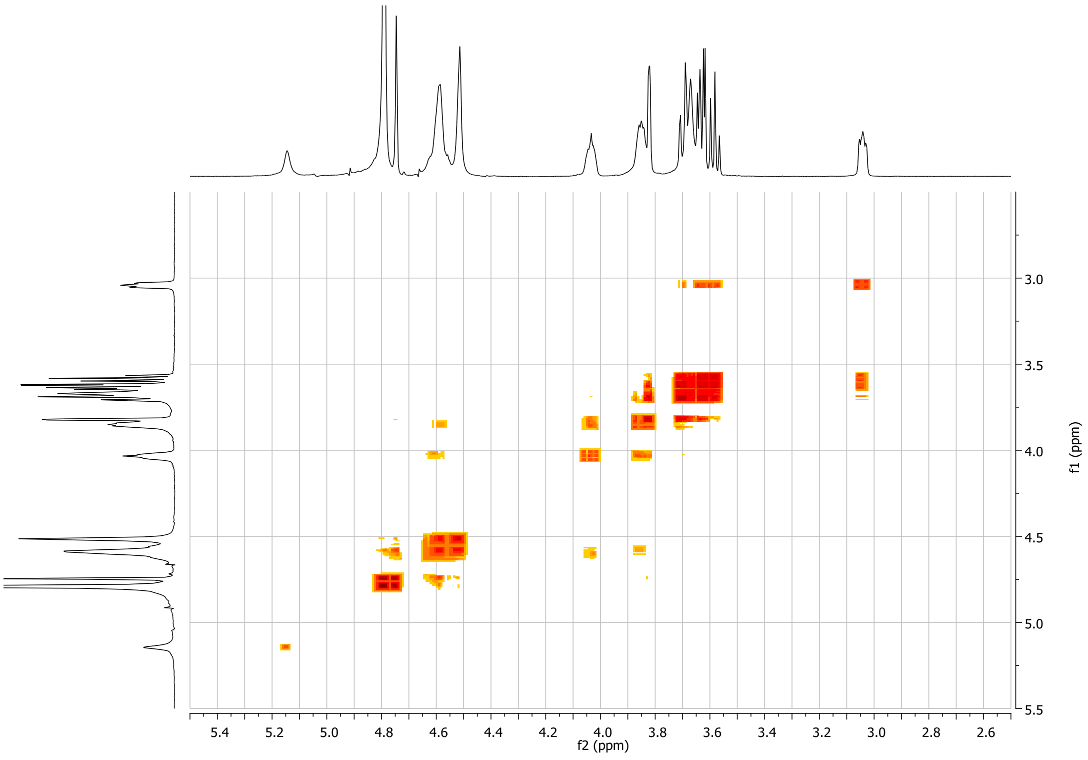

Figure S55: COSY spectrum of compound **23**.

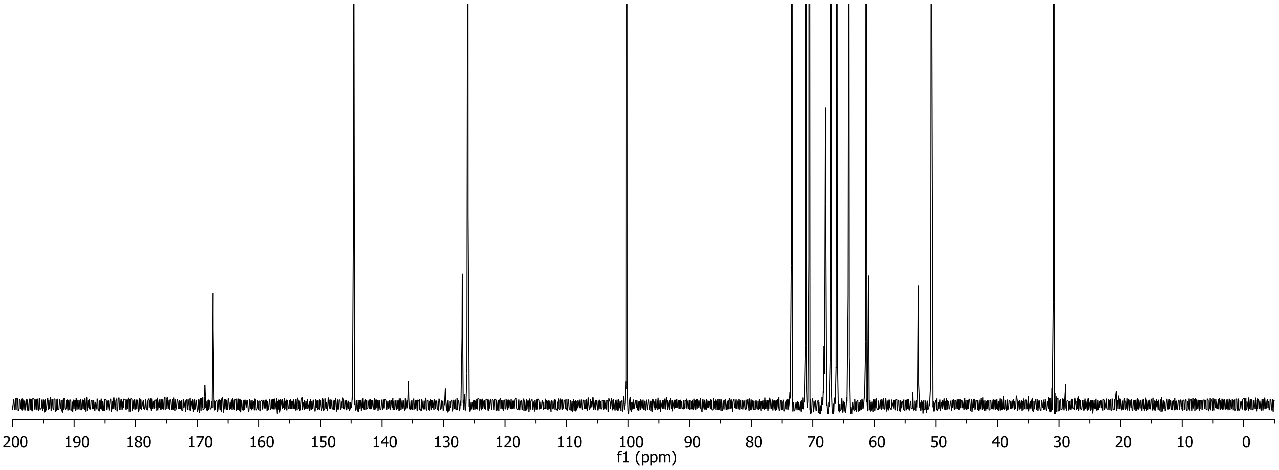

Figure S56: ^{13}C NMR spectrum of compound **23** (D_2O, 150 MHz).

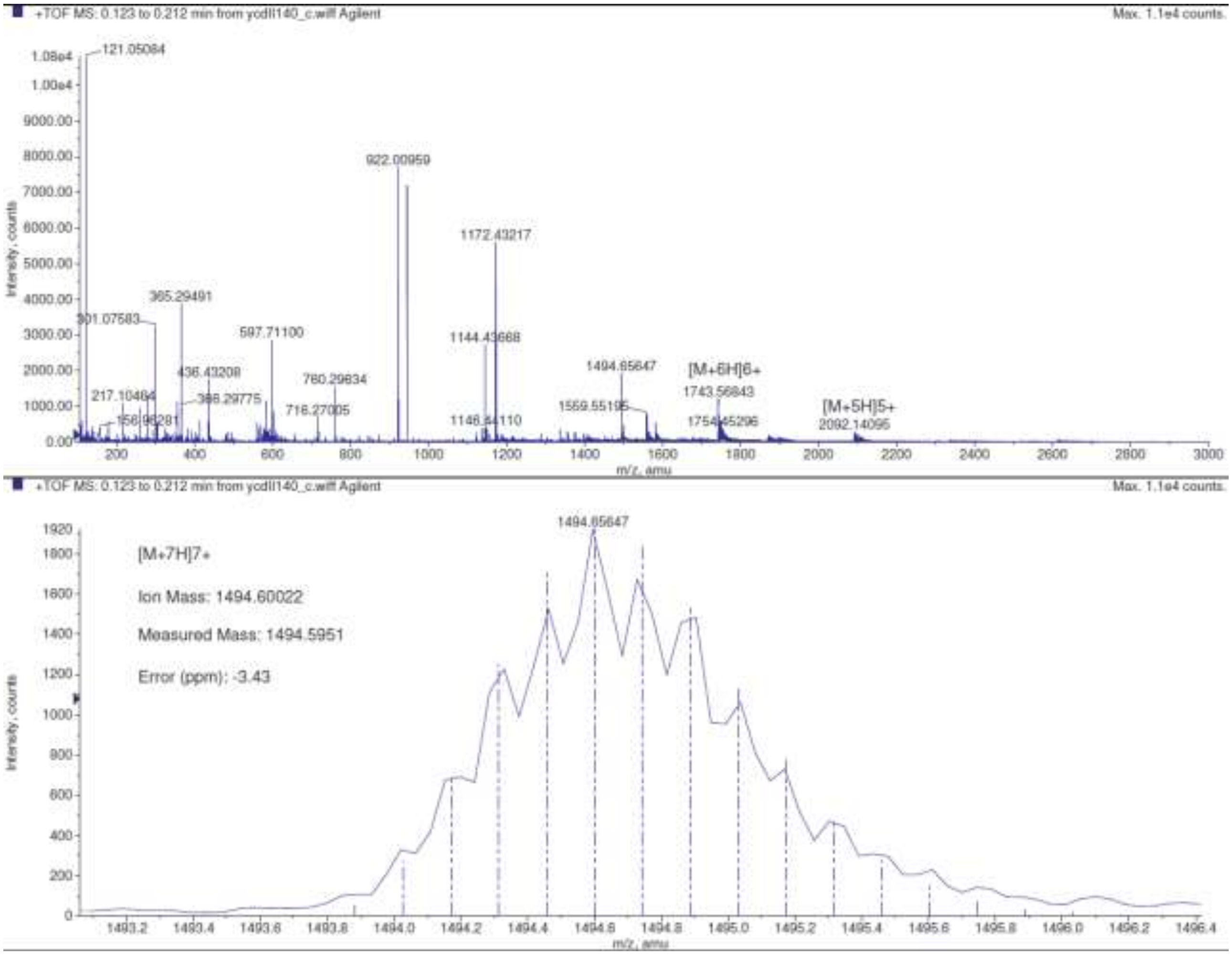

Figure S57: HRMS ($^{+}$TOF) spectrum of compound **23** (+ zoom).

NMR diffusion experiments

The measurement of the diffusion rate (D) allows calculating the solvodynamic diameter of a molecule.[1]

The dendrimers are considered as spherical molecular objects, and characterized by an apparent diffusion coefficient D. The application of the Stokes-Einstein equation gives an estimate of the diameter of the molecule.

Stokes–Einstein equation:

$$D = K_B T / 6\pi\eta r_s$$

D: Diffusion rate ($m^2 \cdot s^{-1}$); K_B: Boltzmann's constant ($k_B = 1.38 \times 10^{-23}$ $m^2 \cdot kg \cdot s^{-2} \cdot K^{-1}$); T: Temperature (K) ($T = 298.15$ K); η: solvent viscosity in Pa s; r_s: Solvodynamic radius of the species.

[1] Diaz, M. D.; Berger, S. *Carbohydr. Res.* **2000**, *329*, 1−5.
